# Supplementary material for: Deciphering the genomes of motility-deficient mutants of Vibrio alginolyticus 138-2
Source: PeerJ. 2024 Mar 18;12:e17126. doi: 10.7717/peerj.17126 (PMC10956519; doi:10.7717/peerj.17126)
Supplement: Supplemental Information 1 — Ref. 1. T. Unemoro and M. Hayashi, Biochim. Biophys,Acta 1969; 171: 89-102 2. Okunishi et al., J Bacteriol. 1996; 178: 2409–2415 3. Kawagishi et al., J Bacteriol. 1995; 177: 5158–516 4. Nishioka et al., J Biochem. 1998;123:1169-73 5. Kusumoto et al., J Biochem. 2006;139:113-21 6. Homma et al., Microbiology 1996;142:2777-83 7. Kojima et al., J Mol Biol 1997;265:310-8 8. Yorimitsu et al., J Bacteriol. 1999;181:5103-6 [file peerj-12-17126-s001.docx]

| **Strain name** | **Parent strain** | **Mutagen** | **Flagella type** | **Putative responsible gene(s) for Pof** | **Mutant categories**  **for Pof** | **reference** | **Total　Number of mutations** |
| --- | --- | --- | --- | --- | --- | --- | --- |
| **138-2**  **138-2** | **-** | **-** | **Pof^+^, Laf^+^** | **-** | **normal** | **1** | **parental strain** |
| **VIO5** | **VIK4** | **EMS** | **Pof^+^, Laf^-^** | **-** | **normal** | **2** | **4** |
| **YM4** | **138-2** | **UV** | **Pof^+^, Laf^-^** | **-** | **normal** | **3** | **14** |
| **YM19** | **YM18** | **UV** | **Pof^-,^ Laf^+^** | **flhA** | **Fla^-^** | **3** | **13** |
| **YM51** | **YM5** | **UV** | **Pof^-^, Laf^-^** | **rpoN** | **Fla^-^** | **4** | **15** |
| **KK148** | **NMB201** | **EMS** | **Pof^+^, Laf^-^** | **flhG** | **Pof^m^** | **5** | **23** |
| **NMB75** | **YM4** | **EMS** | **Pof^+^, Laf^-^** | **cheR** | **Che^-^** | **6** | **23** |
| **NMB82** | **YM4** | **EMS** | **Pof^+^, Laf^-^** | **cheA** | **Che^-^** | **6** | **27** |
| **NMB88** | **YM4** | **EMS** | **Pof^+^, Laf^-^** | **fliM** | **Che^-^ (CCW fixed)** | **6** | **26** |
| **NMB91** | **YM4** | **EMS** | **Pof^+^, Laf^-^** | **zomB** | **Che^-^** | **6** | **27** |
| **NMB93** | **YM4** | **EMS** | **Pof^+^, Laf^-^** | **cheY** | **Che^-^** | **6** | **25** |
| **NMB95** | **YM4** | **EMS** | **Pof^+^, Laf^-^** | **fliM** | **Che^-^ (CCW fixed)** | **6** | **38** |
| **NMB98** | **YM4** | **EMS** | **Pof^+^, Laf^-^** | **zomB** | **Che^-^** | **6** | **28** |
| **NMB99** | **YM4** | **EMS** | **Pof^+^, Laf^-^** | **fliM** | **Che^-^ (CCW fixed)** | **6** | **40** |
| **NMB102** | **YM4** | **EMS** | **Pof^+^, Laf^-^** | **fliM** | **Che^-^ (CW fixed)** | **6** | **40** |
| **NMB103** | **YM4** | **EMS** | **Pof^-^, Laf^-^** | **flgL** | **Fla^-^** | **4, 6** | **26** |
| **NMB105** | **YM4** | **EMS** | **Pof^+^, Laf^-^** | **cheA** | **Che^-^** | **6** | **46** |
| **NMB106** | **YM4** | **EMS** | **Pof^+^, Laf^-^** | **fliM** | **Che^-^ (CCW fixed)** | **6** | **76** |
| **NMB111** | **YM4** | **EMS** | **Pof^+^, Laf^-^** | **flhG** | **Che^-^** | **6** | **32** |
| **NMB116** | **YM4** | **EMS** | **Pof^-^, Laf^-^** | **flgL** | **Pof^-^** | **4, 6** | **38** |
| **NMB136** | **VIO5** | **EMS** | **Pof^+^, Laf^-^** | **cheY** | **Che^-^ (CW fixed)** | **7** | **31** |
|  |  |  |  |  |  |  |  |
| **NMB155** | **VIO5** | **EMS** | **Pof^+^, Laf^-^** | **fliM** | **Pof^m^** | **8** | **18** |
|  |  |  |  |  |  |  |  |
