# Supplementary material for: Deciphering the genomes of motility-deficient mutants of Vibrio alginolyticus 138-2
Source: PeerJ. 2024 Mar 18;12:e17126. doi: 10.7717/peerj.17126 (PMC10956519; doi:10.7717/peerj.17126)
Supplement: Supplemental Information 2 [file peerj-12-17126-s002.docx]

| **PrimerName** | **Sequence(5'->3')** |
| --- | --- |
| contig-1L | CTATTACACAACGCAACAACAAGTG |
| contig-1R | AATAATGCTTGGTAGTGCGTCA |
| contig-2L | AGACAACTGCTCCAGAAAGAAAATC |
| contig-2R | GTGGTCGTGTTTTCCTGTTTCT |
| contig-3L | CATACATCACCTCCCCTTTACTCTC |
| contig-3R | CTTCAGGCACTTTATCGTTTTCAG |
| contig-4L | TAAGCCAAACGACACTTTCTGTAG |
| contig-4R | GACAGCAAGAGATAGAGCCAATACT |
| contig-5L | GTTACTTTACCCTCTGAATGCTCCT |
| contig-5R | CCTACGCTGTTGTCTCTTATTGG |
| contig-6L | GGTATTTCGGGTCAGTTTTCTGT |
| contig-6R | ATTGAGCCAGTCGGTAAGTAGTC |
| contig-7L | CGTTGTAGGATGTTGGTAGATTCAT |
| contig-7R | AGTGCCTATTGAGTTGCTTGAGTT |
| contig-8L | GTCTGTAAGTGAAGATGGCAAGTTA |
| contig-8R | GAAGAAGAGTTTTGGGTAGGGTTAG |
| contig-9L | TAAGGCGGAGTTAGTGTGTGAA |
| contig-9R | GCCATAACGAATAAAGAAGTCCAG |
| contig-10L | TGTAAAAGGCAAACTGTGTAGTGAG |
| contig-10R | AGAAGTGAGGTTCCGTTGTGA |
| contig-11L | GGATTCGTGTGATGTTGCTAAA |
| contig-11R | ATAAACACCAATACGGCGAAAG |
| contig-12L | GTAAAGAGGACCAAGGAAAGGATAC |
| contig-12R | ACTGGACTACCGACATAAAGATAACC |
| contig-13L | ATAAATGCCAAGACCTACAGCAAC |
| contig-13R | ACGATGTAGACTCAAGCGAGATG |
| contig-14L | GAGAAAAAGATGGAAGAGAACAACC |
| contig-14R | GTTCGTTGTGAAGCAGGTAAAA |
| contig-15L | GCAAAGTAAAACAAGACACACACCT |
| contig-15R | AGGATACGGAAGAGGGAAGTAAGT |
| contig-16L | GGTAAGCCCTCAAGAAACAAAATAC |
| contig-16R | TCTGGGCTGTATTTATTAGGCTTG |
| contig-17L | GAGATTGCTAAGGATGATTGAAGG |
| contig-17R | CGCAGAATAAAACCAACCCTATCTA |
| contig-18L | GGACTGACAACACCAAAAAGC |
| contig-18R | GTAGGTTTCGTTTTTGCTCTTCTTC |
| contig-19L | GCTGCTTTACCTTTAGTCGTTGTT |
| contig-19R | CCAGAAATGAAAGTAACACCACAC |
| contig-20L | GAGTGAAGTAATGGATGTGGTTAGC |
| contig-20R | GCTATTACGCCCGACACTTTAG |
| contig-21L | GTTTTGGTGATTGTTTCGTGTG |
| contig-21R | GGGAATGTCAGAGGAAGAAACTC |
| contig-22L | CGATTGTTAGTGAAGTTGGCTCT |
| contig-22R | GTTCACGCATTTCTTACTTCTTCG |
| contig-23L | CTTACGACCTACATCCCTTTTCAAT |
| contig-23R | GCCTACTGATAGAACAAGCATAACA |
| contig-24L | GAAAAATAACCCGCCTAAAAGC |
| contig-24R | GGGTCTCTTTTATCCTTGGTTTAGT |
| contig-25L | TGTTGAAAGCCCGCATTGTG |
| contig-25R | TGCAAGAGGAAAAACGCAGC |
| contig-26L | CCTAAAGCTTTGATTTGAAGTTGAT |
| contig-26R | GGCAAGACAAGTCGCTAGCTTGAAT |
| contig-27L | AAGATAACCAACTGCTACCCAACTA |
| contig-27R | ACGCTTGGCATTTTTCATTC |
| contig-175L1 | AGAGCTTGGCTTTCAGTGCT |
| contig-175L2 | AAAGAGCTTGGCTTTCAGTGC |
| contig-175R1 | CGCACCTCGCTTAAGTGCTA |
| contig-175R2 | AGCACTGAAAGCCAAGCTCT |
| YM4_1L | ATTTAAGAGAAGTCCATCACCCTACC |
| YM4_1R | AAGAGATCGCATTTTTACAGCTAGTG |
| YM4_2L | TAAGTCTTGTACCTGAGCATCAGTAC |
| YM4_2R | TTCTTGATGTACCAACAAATGTCAGG |
| YM4_3L | TACCCGATGGTTCTCTTTTAAAAACG |
| YM4_3R | TCGGGTATAACACTACTTCTCTTTCC |
| YM4_4L | AGAATCCATTAGTGAAGGCTTTTTGG |
| YM4_4R | ATGGTTGTTGTGGGATCTAAAAACTC |
| YM4_5L | AAACGATGGCTAATACCGCATAATAG |
| YM4_5R | TTACCACTCCTTTTGAGATTGTTGTG |
| YM4_6L | ATGTAAAAGGCAAACTGTGTAGTGAG |
| YM4_6R | ACGAACTTGCTGTGTAATAAGCTTAG |
| YM4_7L | ACACCACGAGACATATTAAATTGCTC |
| YM4_7R | TGGTAAAGACATTCAACCACGTAAAG |
| YM4_8L | AATGCTTAGCAGAGATAAGAAATGCC |
| YM4_8R | TTGTTTCGGTAATTTTGGTACTACCG |
| YM4_9L | ACAAGGGTTTAAGCGAAGTTGATAAG |
| YM4_9R | TCTCCCCCGAAAATAACATAACCTAG |
| YM4_10L | TATGTCGATGCTGCTATTTGAATTCC |
| YM4_10R | ATTGCCGGAGAAAATCAAAGAGTATC |
| YM4_11L | TGTAGAAATAACGTGCTCTTTTGACC |
| YM4_11R | ACACTGCCATCTTGAATATTAGTTCG |
| YM4_12L | TTAGAGGTAGGAATGCCAATACTCAG |
| YM4_12R | TACTGCATAGACCTGTCCATATTCAG |
| YM4_13L | TACCCCTAGCTAATCTGTCGTAAAAG |
| YM4_13R | TTATGCGAAACTCACTGATAAGTTCG |
| YM4_14L | TCGGAGCACTTAGTTATAACGACTAG |
| YM4_14R | ATGCAAAAGAAGAACTGATCGAGATC |
| YM4_15L | AATTGCTTTTCGGCAGATAAACATTG |
| YM4_15R | AACCAAAGACTCTACGAAAGCATTAC |
| YM4_16L | TCAAGTCCATCGCTTCTATTCAATTG |
| YM4_16R | AAAAGACGAGGCGATGATGTATAAAG |
| YM4_17L | TCGAGCTTTCACTTTCGATTATCTTG |
| YM4_17R | AACTAAGTGACAAAGGTGTTTGGAAG |
| YM4_18L | ATCATGTTCTCATCTGCGACATAAAC |
| YM4_18R | AAAACTGGCACTACAGATACCAAAAG |
| YM4_19L | TTTATCGTTACTCATGTCAGCATTCG |
| YM4_19R | ACTGAACATTGACCCTACATGTGTAG |
| YM19_2R | TAAAGTACTTTCAGTCGTGAGGAAGG |
