# Supplementary material for: Deciphering the genomes of motility-deficient mutants of Vibrio alginolyticus 138-2
Source: PeerJ. 2024 Mar 18;12:e17126. doi: 10.7717/peerj.17126 (PMC10956519; doi:10.7717/peerj.17126)
Supplement: Supplemental Information 3 — The long unique contigs were arranged at the "contig name" in the order in which the linkages were confirmed by PCR for each of two chromosomes (ChrI, ChrII). The size of the PCR amplification was listed in the "PCR fragment" column, and the sequence of the PCR fragment was listed in the "repeat contig" column. The annotations of repeat contigs and fragments constituting rRNA contigs are described in detail in Table S4. [file peerj-12-17126-s003.docx]

| **ChrI** | **contig name** | **size(bp)** | **PCRfragment** | **repeat contigs** |
| --- | --- | --- | --- | --- |
|  | NODE_4 | 510848 |  |  |
|  |  |  | 2.7kbp | NODE_31 |
|  | NODE_20 | 27651 |  |  |
|  |  |  | 8.0kbp | rRNA_contigs |
|  | NODE_8 | 246946 |  |  |
|  |  |  | 2.5kbp | NODE_51 |
|  | NODE_10 | 172834 |  |  |
|  |  |  | 2.5kbp | NODE_31 |
|  | NODE_9 | 191065 |  |  |
|  |  |  | 2.0kbp | NODE_32 |
|  | NODE_11 | 142781 |  |  |
|  |  |  | 2.0kbp | NODE_55 |
|  | NODE_23 | 11221 |  |  |
|  |  |  | 1.2kbp | NODE_73 |
|  | NODE_26 | 4369 |  |  |
|  |  |  | 1.5kbp | NODE_38 |
|  | NODE_27 | 3687 |  |  |
|  |  |  | 3.0kb | NODE_73 |
|  | NODE_25 | 4799 |  |  |
|  |  |  | 3.0kb | NODE_31 |
|  | NODE_18 | 31538 |  |  |
|  |  |  | 3.0kbp | NODE_38, 55 |
|  | NODE_5 | 357453 |  |  |
|  |  |  | 1.5kbp | NODE_31 |
|  | NODE_6 | 306405 |  |  |
|  |  |  | 2.0kbp | NODE_32 |
|  | NODE_7 | 267624 |  |  |
|  |  |  | 2.5kbp | NODE_51 |
|  | NODE_3 | 512880 |  |  |
|  |  |  | 8.0kbp | rRNA_contigs |
|  | NODE_22 | 12852 |  |  |
|  |  |  | 2.5kbp | NODE_42 |
|  | NODE_14 | 1155511 |  |  |
|  |  |  | 15kbp | NODE_31, rRNA x2 |
|  | NODE_21 | 15268 |  |  |
|  |  |  | 2.0kbp | NODE_42 |
|  | NODE_16 | 54024 |  |  |
|  |  |  | 2.5kbp | NODE_33 |
|  | NODE_13 | 116772 |  |  |
|  |  |  | 16kbp | rRNA x2 |
|  | NODE_19 | 31095 |  |  |
|  |  |  | 2.5kbp | NODE_33 |
|  | NODE_24 | 6571 |  |  |
|  |  |  | 15kbp | rRNA_ x2 |
|  | NODE_15 | 54649 |  |  |
|  |  |  | 9.0kbp | rRNA_contigs |
|  | NODE_17 | 36940 |  |  |
|  |  |  | 8.0kbp | rRNA_contigs |
|  | NODE_12 | 121027 |  |  |
|  |  |  | 8.0kbp | rRNA_contigs |
|  | NODE_4 | 510848 |  |  |
| **Chr**II**II** | **contig name** | **size** | **PCR fragment** | **repeat contigs** |
|  | NODE_1 | 1042398 |  |  |
|  |  |  | 2.5kbp | NODE_31 |
|  | NODE_2 | 710788 |  |  |
|  |  |  | 8.0kbp | rRNA_contigs |
|  | NODE_1 | 1042398 |  |  |
