# Supplementary material for: Deciphering the genomes of motility-deficient mutants of Vibrio alginolyticus 138-2
Source: PeerJ. 2024 Mar 18;12:e17126. doi: 10.7717/peerj.17126 (PMC10956519; doi:10.7717/peerj.17126)
Supplement: Supplemental Information 4 [file peerj-12-17126-s004.docx]

(A)

| contig | putativecodinggene(s) | copynumber |
| --- | --- | --- |
| NODE_31 | IS3familytransposase | 6 |
| NODE_32 | IS110familytransposase | 2 |
| NODE_33 | elongationfactoTu | 2 |
| NODE_38 | hypotheticalprotein | 2 |
| NODE_42 | IS5familytransposase | 2 |
| NODE_51 | flagellin_N | 2 |
| NODE_55 | ferrichrome-bindingprotein | 2 |
| NODE_73 | hypotheticalprotein | 2 |

(B)

| rRNA_contigs | annotation |
| --- | --- |
| NODE_28 | 23SrRNA |
| NODE_30 | 16SrRNA |
| NODE_34 | 16SrRNA |
| NODE_35 | 16SrRNA |
| NODE_36 | 23SrRNA, 5SrRNA, tRNA |
| NODE_40 | 16SrRNA |
| NODE_44 | tRNA |
| NODE_45 | tRNA |
| NODE_49 | tRNA, 16SrRNA |
| NODE_68 | 23SrRNA, 16SrRNA |
| NODE_175 | inter RNA operons region |
