# Supplementary material for: Deciphering the genomes of motility-deficient mutants of Vibrio alginolyticus 138-2
Source: PeerJ. 2024 Mar 18;12:e17126. doi: 10.7717/peerj.17126 (PMC10956519; doi:10.7717/peerj.17126)
Supplement: Supplemental Information 5 [file peerj-12-17126-s005.docx]

| Vibrio strains | **138-2** | **VIO5** | **YM4** | **YM19** |
| --- | --- | --- | --- | --- |
| Chromosome size (Mb) |  |  |  |  |
| chromosome I | 3426062 | 3426062 | 3425997 | 3425997 |
| chromosome II | 1759333 | 1759333 | 1759327 | 1759327 |
| Avg. G+C (%) |  |  |  |  |
| chromosome I | 45.0 | 45.0 | 45.0 | 45.0 |
| chromosome II | 44.9 | 44.9 | 44.9 | 44.9 |
| Protein Coding Sequences |  |  |  |  |
| chromosome I | 3052 | 3052 | 3053 | 3053 |
| chromosome II | 1550 | 1549 | 1550 | 1550 |
| Ribosomal RNA operons |  |  |  |  |
| chromosome I | 34 | 34 | 34 | 34 |
| chromosome II | 3 | 3 | 3 | 3 |
| Numbers of tRNA |  |  |  |  |
| chromosome I | 103 | 103 | 103 | 103 |
| chromosome II | 13 | 13 | 13 | 13 |
