# Supplementary material for: Deciphering the genomes of motility-deficient mutants of Vibrio alginolyticus 138-2
Source: PeerJ. 2024 Mar 18;12:e17126. doi: 10.7717/peerj.17126 (PMC10956519; doi:10.7717/peerj.17126)
Supplement: Supplemental Information 7 — Using 104 proteins associated with the polar and lateral flagella as queries, blastp-based search was performed on all CDS proteins of V. alginolyticus strain 138-2, and those detected with an E-value of 10 or less were listed. Those marked in yellow represent query own hits. Those marked in orange represent proteins in a paralogous relationship between the polar and lateral flagella. Those marked in light gray represent seven similar proteins encoding flagellin fibers, six of which correspond to the polar flagellum and one to the lateral flagellum. [file peerj-12-17126-s007.docx]

| **query** | **subject protein and annotation** | **length** | **match** | **% identity** | **E-value** |
| --- | --- | --- | --- | --- | --- |
| FlgT | BCB41585.1 hypothetical protein Vag1382_07110 | 377 | 377 | 100.0 | 0 |
|  | BCB41739.1 2-succinyl-5-enolpyruvyl-6-hydroxy-3-cyclohexene-1-carboxylate synthase | 76 | 21 | 27.6 | 1.2 |
|  | BCB41028.1 hypothetical protein Vag1382_01540 | 29 | 14 | 48.3 | 2.4 |
|  | BCB41569.1 5'-nucleotidase | 30 | 9 | 30.0 | 2.5 |
|  | BCB40893.1 fatty acid oxidation complex subunit alpha | 81 | 25 | 30.9 | 4.4 |
|  | BCB41721.1 C4-dicarboxylate ABC transporter | 42 | 9 | 21.4 | 8.3 |
| FlgO | BCB41586.1 membrane protein | 212 | 212 | 100.0 | 4.88E-160 |
|  | BCB43282.1 histidine kinase | 53 | 19 | 35.8 | 1.3 |
|  | BCB42989.1 ribonuclease HI | 33 | 10 | 30.3 | 2.5 |
|  | BCB42669.1 methyltransferase | 25 | 13 | 52.0 | 4.3 |
|  | BCB42212.1 7-carboxy-7-deazaguanine synthase | 125 | 26 | 20.8 | 5.1 |
|  | BCB43167.1 hypothetical protein Vag1382_22940 | 25 | 10 | 40.0 | 9 |
| FlgP | BCB41587.1 hypothetical protein Vag1382_07130 | 143 | 143 | 100.0 | 9.27E-101 |
|  | BCB43424.1 membrane-bound lytic murein transglycosylase C | 16 | 9 | 56.3 | 0.41 |
|  | BCB43585.1 ribulose-phosphate 3-epimerase | 32 | 12 | 37.5 | 0.87 |
|  | BCB41336.1 peptide chain release factor 2 | 80 | 20 | 25.0 | 2.1 |
|  | BCB42006.1 iron-regulated protein A | 20 | 13 | 65.0 | 2.2 |
|  | BCB41474.1 hypothetical protein Vag1382_06000 | 19 | 10 | 52.6 | 2.8 |
|  | BCB42159.1 EscJ/YscJ/HrcJ family type III secretion inner membrane ring protein | 19 | 11 | 57.9 | 3 |
|  | BCB41881.1 multidrug transporter | 17 | 8 | 47.1 | 3.6 |
|  | BCB45319.1 multidrug resistance protein MdtL | 15 | 9 | 60.0 | 4 |
|  | BCB44610.1 DNA-binding response regulator | 27 | 12 | 44.4 | 4.4 |
|  | BCB43504.1 lactate dehydrogenase | 47 | 16 | 34.0 | 5 |
|  | BCB44685.1 LysR family transcriptional regulator | 14 | 8 | 57.1 | 5 |
|  | BCB43390.1 D-3-phosphoglycerate dehydrogenase | 46 | 14 | 30.4 | 6.5 |
|  | BCB41663.1 succinate--CoA ligase [ADP-forming] subunit alpha | 41 | 11 | 26.8 | 9.3 |
| FlgN1 | BCB41588.1 molecular chaperone | 141 | 141 | 100.0 | 6.11E-102 |
|  | BCB42204.1 citrate synthase | 68 | 18 | 26.5 | 0.92 |
|  | BCB45088.1 GGDEF domain-containing protein | 30 | 12 | 40.0 | 2.2 |
|  | BCB43143.1 hypothetical protein Vag1382_22700 | 51 | 10 | 19.6 | 2.7 |
|  | BCB44166.1 flagellar hook-associated protein 1 FlgK | 62 | 19 | 30.6 | 3.6 |
|  | BCB44154.1 protein FlgN | 20 | 10 | 50.0 | 4 |
|  | BCB44772.1 AraC family transcriptional regulator | 25 | 9 | 36.0 | 4.6 |
|  | BCB42200.1 bordetella uptake gene family protein | 56 | 15 | 26.8 | 5 |
|  | BCB45337.1 D-alanyl-D-alanine carboxypeptidase | 77 | 25 | 32.5 | 6.1 |
| FlgM1 | BCB41589.1 flagellar biosynthesis anti-sigma factor FlgM | 104 | 104 | 100.0 | 5.43E-73 |
|  | BCB42369.1 ATP-dependent RNA helicase HrpA | 69 | 18 | 26.1 | 5.3 |
| FlgA1 | BCB41590.1 flagella basal body P-ring formation protein FlgA | 248 | 248 | 100.0 | 0 |
|  | BCB44156.1 flagella basal body P-ring formation protein FlgA | 217 | 60 | 27.7 | 3.87E-21 |
|  | BCB43543.1 sodium:proton antiporter | 54 | 16 | 29.6 | 0.23 |
|  | BCB42689.1 ABC transporter ATP-binding protein | 29 | 11 | 37.9 | 1.5 |
|  | BCB42454.1 hypothetical protein Vag1382_15800 | 34 | 9 | 26.5 | 2.5 |
|  | BCB44147.1 MFS transporter | 33 | 13 | 39.4 | 4.4 |
|  | BCB41632.1 transmembrane regulatory protein ToxS | 56 | 19 | 33.9 | 5.8 |
|  | BCB43845.1 protein Smg | 22 | 8 | 36.4 | 7.9 |
| CheV | BCB41591.1 chemotaxis protein CheW | 308 | 308 | 100.0 | 0 |
|  | BCB42751.1 chemotaxis protein CheW | 315 | 142 | 45.1 | 8.97E-91 |
|  | BCB45119.1 chemotaxis protein CheW | 283 | 103 | 36.4 | 2.66E-52 |
|  | BCB44611.1 chemotaxis protein CheV | 298 | 99 | 33.2 | 6.06E-44 |
|  | BCB42920.1 chemotaxis protein CheW | 138 | 41 | 29.7 | 3.66E-13 |
|  | BCB43282.1 histidine kinase | 132 | 40 | 30.3 | 2.72E-09 |
|  | BCB44359.1 hybrid sensor histidine kinase/response regulator | 137 | 41 | 29.9 | 1.53E-08 |
|  | BCB45406.1 DNA-binding response regulator | 126 | 34 | 27.0 | 1.54E-07 |
|  | BCB41314.1 DNA-binding response regulator | 127 | 33 | 26.0 | 3.87E-07 |
|  | BCB42342.1 transcriptional regulator | 112 | 38 | 33.9 | 4.21E-07 |
|  | BCB41007.1 DNA-binding response regulator | 127 | 35 | 27.6 | 9.87E-07 |
|  | BCB44424.1 transcriptional regulator | 125 | 40 | 32.0 | 1.38E-06 |
|  | BCB44508.1 histidine kinase | 75 | 27 | 36.0 | 2.69E-06 |
|  | BCB44604.1 hybrid sensor histidine kinase/response regulator | 112 | 32 | 28.6 | 4.49E-06 |
|  | BCB44868.1 hypothetical protein Vag1382_39950 | 115 | 30 | 26.1 | 5.22E-06 |
|  | BCB42926.1 response regulator | 111 | 32 | 28.8 | 1.81E-05 |
|  | BCB42881.1 two-component system response regulator | 127 | 35 | 27.6 | 3.28E-05 |
|  | BCB42502.1 two-component system sensor histidine kinase/response regulator | 75 | 26 | 34.7 | 3.33E-05 |
|  | BCB42502.1 two-component system sensor histidine kinase/response regulator | 101 | 28 | 27.7 | 3.49E-04 |
|  | BCB40971.1 nitrogen regulation protein NR(I) | 129 | 37 | 28.7 | 4.21E-05 |
|  | BCB41843.1 two-component system response regulator TorR | 126 | 33 | 26.2 | 6.93E-05 |
|  | BCB43932.1 hybrid sensor histidine kinase/response regulator | 110 | 34 | 30.9 | 1.92E-04 |
|  | BCB44762.1 diguanylate cyclase response regulator | 74 | 25 | 33.8 | 2.95E-04 |
|  | BCB42923.1 chemotaxis response regulator protein-glutamate methylesterase of group 1 operon CheB | 114 | 35 | 30.7 | 0.001 |
|  | BCB44084.1 two-component system response regulator | 126 | 29 | 23.0 | 0.001 |
|  | BCB41957.1 hybrid sensor histidine kinase/response regulator | 76 | 22 | 28.9 | 0.001 |
|  | BCB44327.1 DNA-binding response regulator | 119 | 28 | 23.5 | 0.001 |
|  | BCB41392.1 DNA-binding response regulator | 128 | 30 | 23.4 | 0.001 |
|  | BCB42946.1 sigma-54-dependent Fis family transcriptional regulator FlaM | 121 | 29 | 24.0 | 0.011 |
|  | BCB43942.1 putative response regulatory protein | 79 | 20 | 25.3 | 0.014 |
|  | BCB44537.1 hybrid sensor histidine kinase/response regulator | 110 | 34 | 30.9 | 0.02 |
|  | BCB42039.1 hybrid sensor histidine kinase/response regulator | 100 | 29 | 29.0 | 0.092 |
|  | BCB43366.1 histidine kinase | 84 | 26 | 31.0 | 0.14 |
|  | BCB43692.1 DNA-binding response regulator | 124 | 26 | 21.0 | 0.14 |
|  | BCB42285.1 hybrid sensor histidine kinase/response regulator | 83 | 24 | 28.9 | 0.35 |
|  | BCB41878.1 sensor histidine kinase | 113 | 32 | 28.3 | 0.44 |
|  | BCB41215.1 DNA-binding response regulator | 114 | 27 | 23.7 | 0.55 |
|  | BCB44754.1 DNA-binding response regulator | 77 | 22 | 28.6 | 0.62 |
|  | BCB45455.1 DNA-binding response regulator | 124 | 31 | 25.0 | 0.66 |
|  | BCB42354.1 sigma-54-dependent Fis family transcriptional regulator | 123 | 31 | 25.2 | 0.69 |
|  | BCB44366.1 autoinducer 2 sensor kinase/phosphatase LuxQ | 114 | 28 | 24.6 | 0.74 |
|  | BCB44049.1 DNA-binding response regulator | 125 | 30 | 24.0 | 1 |
|  | BCB41165.1 DUF490 domain-containing protein | 64 | 20 | 31.3 | 1.2 |
|  | BCB44892.1 DNA-binding response regulator | 75 | 24 | 32.0 | 1.3 |
|  | BCB43169.1 heat-shock protein Hsp20 | 30 | 11 | 36.7 | 1.4 |
|  | BCB41942.1 DNA-binding response regulator | 76 | 16 | 21.1 | 3 |
|  | BCB41312.1 aerobic respiration control sensor protein | 68 | 22 | 32.4 | 3.1 |
|  | BCB44946.1 FAD-binding oxidoreductase | 41 | 11 | 26.8 | 4.9 |
|  | BCB44492.1 3-oxoacyl-ACP reductase | 47 | 16 | 34.0 | 5.2 |
|  | BCB42017.1 DNA-binding response regulator | 136 | 36 | 26.5 | 5.5 |
|  | BCB44340.1 cytosine permease | 65 | 17 | 26.2 | 5.8 |
|  | BCB45130.1 transcriptional regulator | 61 | 18 | 29.5 | 6.5 |
|  | BCB42610.1 thioredoxin reductase | 25 | 11 | 44.0 | 7.4 |
|  | BCB43065.1 cysteine desulfurase | 58 | 14 | 24.1 | 7.6 |
|  | BCB42407.1 sensor histidine kinase | 45 | 16 | 35.6 | 8.6 |
|  | BCB44778.1 acriflavine resistance protein B | 41 | 12 | 29.3 | 9.4 |
|  | BCB42438.1 DNA-binding response regulator | 77 | 19 | 24.7 | 9.8 |
| CheR | BCB41592.1 chemotaxis protein methyltransferase CheR | 275 | 275 | 100.0 | 0 |
|  | BCB41692.1 SAM-dependent methyltransferase | 66 | 17 | 25.8 | 0.17 |
|  | BCB42565.1 thiopurine S-methyltransferase | 42 | 13 | 31.0 | 0.36 |
|  | BCB42707.1 peptidyl-prolyl cis-trans isomerase | 24 | 11 | 45.8 | 1.1 |
|  | BCB44111.1 hypothetical protein Vag1382_32380 | 28 | 10 | 35.7 | 6.7 |
|  | BCB41854.1 carboxy-S-adenosyl-L-methionine synthase | 28 | 10 | 35.7 | 8.8 |
| FlgB1 | BCB41593.1 flagellar basal body rod protein FlgB | 131 | 131 | 100.0 | 4.64E-96 |
|  | BCB44157.1 flagellar basal body rod protein FlgB | 130 | 50 | 38.5 | 8.25E-24 |
|  | BCB42293.1 copper-translocating P-type ATPase | 31 | 12 | 38.7 | 0.18 |
|  | BCB44366.1 autoinducer 2 sensor kinase/phosphatase LuxQ | 69 | 18 | 26.1 | 0.58 |
|  | BCB44410.1 GGDEF-domain containing protein | 42 | 14 | 33.3 | 1.3 |
|  | BCB41598.1 flagellar basal-body rod protein FlgG | 32 | 12 | 37.5 | 2.4 |
|  | BCB42249.1 dihydroorotate dehydrogenase (quinone) | 58 | 16 | 27.6 | 4.8 |
|  | BCB42892.1 beta-ketoacyl-[acyl-carrier-protein] synthase I | 92 | 26 | 28.3 | 5 |
|  | BCB42213.1 7-cyano-7-deazaguanine synthase | 50 | 14 | 28.0 | 5.7 |
|  | BCB44397.1 MexE family multidrug efflux RND transporter periplasmic adaptor subunit | 46 | 16 | 34.8 | 8.7 |
|  | BCB41596.1 flagellar hook protein FlgE | 20 | 11 | 55.0 | 9.2 |
|  | BCB44787.1 oxidoreductase alpha (molybdopterin) subunit | 25 | 12 | 48.0 | 9.2 |
|  | BCB41042.1 phosphopantetheine adenylyltransferase | 24 | 9 | 37.5 | 9.7 |
| FlgC1 | BCB41594.1 flagellar basal-body rod protein FlgC | 137 | 137 | 100.0 | 1.83E-100 |
|  | BCB44158.1 flagellar basal-body rod protein FlgC | 142 | 59 | 41.5 | 1.84E-35 |
|  | BCB44162.1 flagellar basal-body rod protein FlgG | 40 | 16 | 40.0 | 2.13E-04 |
|  | BCB44162.1 flagellar basal-body rod protein FlgG | 41 | 12 | 29.3 | 6.1 |
|  | BCB41598.1 flagellar basal-body rod protein FlgG | 32 | 16 | 50.0 | 6.64E-04 |
|  | BCB41598.1 flagellar basal-body rod protein FlgG | 41 | 15 | 36.6 | 0.7 |
|  | BCB43585.1 ribulose-phosphate 3-epimerase | 64 | 20 | 31.3 | 0.1 |
|  | BCB41596.1 flagellar hook protein FlgE | 44 | 12 | 27.3 | 0.12 |
|  | BCB41596.1 flagellar hook protein FlgE | 49 | 18 | 36.7 | 0.19 |
|  | BCB44166.1 flagellar hook-associated protein 1 FlgK | 37 | 12 | 32.4 | 1.1 |
|  | BCB43647.1 ribonuclease R | 50 | 16 | 32.0 | 1.4 |
|  | BCB42694.1 trimethylamine-N-oxide reductase | 30 | 12 | 40.0 | 1.4 |
|  | BCB44905.1 hypothetical protein Vag1382_40320 | 21 | 8 | 38.1 | 1.6 |
|  | BCB44160.1 flagellar hook protein FlgE | 25 | 12 | 48.0 | 1.9 |
|  | BCB44160.1 flagellar hook protein FlgE | 37 | 10 | 27.0 | 2.2 |
|  | BCB40952.1 DNA recombination protein RmuC | 90 | 24 | 26.7 | 3.8 |
|  | BCB40898.1 ketol-acid reductoisomerase (NADP(+)) | 32 | 9 | 28.1 | 9.1 |
| FlgD1 | BCB41595.1 basal-body rod modification protein FlgD | 236 | 236 | 100.0 | 4.98E-173 |
|  | BCB44159.1 basal-body rod modification protein FlgD | 147 | 40 | 27.2 | 8.39E-08 |
|  | BCB44060.1 hypothetical protein Vag1382_31870 | 35 | 13 | 37.1 | 0.04 |
|  | BCB41032.1 nucleoid occlusion factor SlmA | 39 | 12 | 30.8 | 2.1 |
|  | BCB41397.1 phosphate ABC transporter permease | 58 | 17 | 29.3 | 2.8 |
|  | BCB45211.1 phosphate import ATP-binding protein PstB 2 | 58 | 19 | 32.8 | 2.9 |
|  | BCB43608.1 methylenetetrahydrofolate reductase | 46 | 15 | 32.6 | 5.2 |
|  | BCB40877.1 DNA replication and repair protein RecF | 29 | 12 | 41.4 | 7.7 |
|  | BCB44838.1 hypothetical protein Vag1382_39650 | 72 | 22 | 30.6 | 9.9 |
| FlgE1 | BCB41596.1 flagellar hook protein FlgE | 437 | 437 | 100.0 | 0 |
|  | BCB44160.1 flagellar hook protein FlgE | 440 | 151 | 34.3 | 1.84E-60 |
|  | BCB41598.1 flagellar basal-body rod protein FlgG | 147 | 58 | 39.5 | 8.53E-15 |
|  | BCB41598.1 flagellar basal-body rod protein FlgG | 82 | 28 | 34.1 | 1.26E-07 |
|  | BCB44162.1 flagellar basal-body rod protein FlgG | 134 | 46 | 34.3 | 2.46E-12 |
|  | BCB44162.1 flagellar basal-body rod protein FlgG | 81 | 28 | 34.6 | 1.45E-07 |
|  | BCB41597.1 flagellar basal body protein FlgF | 132 | 34 | 25.8 | 4.47E-05 |
|  | BCB41597.1 flagellar basal body protein FlgF | 44 | 10 | 22.7 | 9.1 |
|  | BCB44161.1 flagellar basal body protein FlgF | 115 | 34 | 29.6 | 0.002 |
|  | BCB44161.1 flagellar basal body protein FlgF | 37 | 12 | 32.4 | 0.79 |
|  | BCB44166.1 flagellar hook-associated protein 1 FlgK | 38 | 16 | 42.1 | 0.007 |
|  | BCB44158.1 flagellar basal-body rod protein FlgC | 44 | 14 | 31.8 | 0.016 |
|  | BCB44158.1 flagellar basal-body rod protein FlgC | 81 | 22 | 27.2 | 2.5 |
|  | BCB42424.1 L-ectoine synthase | 95 | 22 | 23.2 | 0.039 |
|  | BCB41594.1 flagellar basal-body rod protein FlgC | 44 | 12 | 27.3 | 0.37 |
|  | BCB41594.1 flagellar basal-body rod protein FlgC | 49 | 18 | 36.7 | 0.6 |
|  | BCB43274.1 tyrosine--tRNA ligase 2 | 60 | 20 | 33.3 | 1.5 |
|  | BCB41823.1 ribosomal large subunit pseudouridine synthase E | 90 | 22 | 24.4 | 1.9 |
|  | BCB44997.1 alkaline phosphatase | 58 | 18 | 31.0 | 2.6 |
|  | BCB41602.1 flagellar hook protein FlgK | 20 | 12 | 60.0 | 2.9 |
|  | BCB44253.1 hypothetical protein Vag1382_33800 | 72 | 18 | 25.0 | 3.1 |
|  | BCB41298.1 hypothetical protein Vag1382_04240 | 33 | 11 | 33.3 | 7.3 |
|  | BCB43623.1 peptidyl-prolyl cis-trans isomerase | 46 | 19 | 41.3 | 8.1 |
|  | BCB43557.1 sn-glycerol-3-phosphate dehydrogenase subunit C | 78 | 21 | 26.9 | 9.3 |
| FlgF1 | BCB41597.1 flagellar basal body protein FlgF | 249 | 249 | 100.0 | 0 |
|  | BCB44161.1 flagellar basal body protein FlgF | 246 | 103 | 41.9 | 1.43E-55 |
|  | BCB44162.1 flagellar basal-body rod protein FlgG | 263 | 70 | 26.6 | 4.46E-16 |
|  | BCB41598.1 flagellar basal-body rod protein FlgG | 268 | 74 | 27.6 | 1.41E-15 |
|  | BCB44160.1 flagellar hook protein FlgE | 118 | 35 | 29.7 | 2.65E-06 |
|  | BCB44160.1 flagellar hook protein FlgE | 43 | 16 | 37.2 | 3.16E-04 |
|  | BCB41596.1 flagellar hook protein FlgE | 132 | 34 | 25.8 | 2.56E-05 |
|  | BCB41596.1 flagellar hook protein FlgE | 44 | 10 | 22.7 | 5.2 |
|  | BCB44166.1 flagellar hook-associated protein 1 FlgK | 28 | 13 | 46.4 | 0.91 |
|  | BCB41992.1 DNA gyrase subunit A | 52 | 20 | 38.5 | 1.9 |
|  | BCB42569.1 phenylalanine--tRNA ligase beta subunit | 30 | 11 | 36.7 | 7.4 |
| FlgG1 | BCB41598.1 flagellar basal-body rod protein FlgG | 262 | 262 | 100.0 | 0 |
|  | BCB44162.1 flagellar basal-body rod protein FlgG | 262 | 143 | 54.6 | 2.55E-92 |
|  | BCB44160.1 flagellar hook protein FlgE | 111 | 43 | 38.7 | 1.41E-15 |
|  | BCB44160.1 flagellar hook protein FlgE | 132 | 44 | 33.3 | 7.80E-10 |
|  | BCB41597.1 flagellar basal body protein FlgF | 268 | 74 | 27.6 | 1.49E-15 |
|  | BCB41596.1 flagellar hook protein FlgE | 147 | 58 | 39.5 | 5.18E-15 |
|  | BCB41596.1 flagellar hook protein FlgE | 82 | 28 | 34.1 | 7.63E-08 |
|  | BCB44161.1 flagellar basal body protein FlgF | 265 | 69 | 26.0 | 2.86E-11 |
|  | BCB41594.1 flagellar basal-body rod protein FlgC | 32 | 16 | 50.0 | 0.001 |
|  | BCB41594.1 flagellar basal-body rod protein FlgC | 41 | 15 | 36.6 | 1.3 |
|  | BCB44857.1 serine/threonine protein phosphatase | 182 | 43 | 23.6 | 0.73 |
|  | BCB44575.1 histidine kinase | 28 | 12 | 42.9 | 1.5 |
|  | BCB43998.1 hemolysin D | 38 | 12 | 31.6 | 1.7 |
|  | BCB44158.1 flagellar basal-body rod protein FlgC | 45 | 13 | 28.9 | 2.9 |
|  | BCB42753.1 pyruvate kinase | 70 | 22 | 31.4 | 2.9 |
|  | BCB45245.1 methyl-accepting chemotaxis protein | 46 | 15 | 32.6 | 3 |
|  | BCB44166.1 flagellar hook-associated protein 1 FlgK | 35 | 11 | 31.4 | 3.3 |
|  | BCB45143.1 membrane protein | 55 | 17 | 30.9 | 3.3 |
|  | BCB41602.1 flagellar hook protein FlgK | 30 | 11 | 36.7 | 4.6 |
|  | BCB41593.1 flagellar basal body rod protein FlgB | 32 | 12 | 37.5 | 4.8 |
|  | BCB41969.1 phosphatase | 21 | 9 | 42.9 | 5.2 |
| FlgH1 | BCB41599.1 flagellar L-ring protein 1 FlgH | 259 | 259 | 100.0 | 0 |
|  | BCB44163.1 flagellar L-ring protein 2 FlgH | 198 | 73 | 36.9 | 2.08E-39 |
|  | BCB44286.1 hypothetical protein Vag1382_34130 | 37 | 13 | 35.1 | 0.52 |
|  | BCB43466.1 hypothetical protein Vag1382_25930 | 61 | 18 | 29.5 | 1.3 |
|  | BCB44738.1 hypothetical protein Vag1382_38650 | 35 | 10 | 28.6 | 1.9 |
|  | BCB44728.1 hypothetical protein Vag1382_38550 | 35 | 10 | 28.6 | 1.9 |
|  | BCB41049.1 glucose-1-phosphate thymidylyltransferase | 61 | 16 | 26.2 | 2.1 |
|  | BCB41726.1 trigger factor | 111 | 22 | 19.8 | 4.6 |
|  | BCB41812.1 ABC transporter substrate-binding protein | 39 | 14 | 35.9 | 7.7 |
|  | BCB41121.1 30S ribosomal protein S3 | 79 | 22 | 27.8 | 9.5 |
|  | BCB44457.1 type VI secretion lipoprotein | 38 | 12 | 31.6 | 9.7 |
|  | BCB42956.1 deferrochelatase/peroxidase YfeX | 46 | 12 | 26.1 | 9.8 |
| FlgI1 | BCB41600.1 flagellar P-ring protein 1 FlgI | 363 | 363 | 100.0 | 0 |
|  | BCB44164.1 flagellar P-ring protein 2 FlgI | 362 | 178 | 49.2 | 6.67E-120 |
|  | BCB44522.1 GGDEF domain-containing protein | 48 | 15 | 31.3 | 1.6 |
|  | BCB42257.1 ATP-dependent protease | 33 | 12 | 36.4 | 2.5 |
|  | BCB42412.1 cell signaling regulator | 119 | 26 | 21.8 | 2.9 |
|  | BCB42412.1 cell signaling regulator | 31 | 11 | 35.5 | 9.6 |
|  | BCB42169.1 3-oxoacyl-ACP reductase | 55 | 18 | 32.7 | 3.2 |
|  | BCB42019.1 BCCT family transporter | 69 | 22 | 31.9 | 4 |
|  | BCB40977.1 DNA repair ATPase | 99 | 19 | 19.2 | 4.3 |
|  | BCB41530.1 methionine import ATP-binding protein MetN | 152 | 32 | 21.1 | 5.3 |
|  | BCB43176.1 hypothetical protein Vag1382_23030 | 82 | 25 | 30.5 | 6.8 |
|  | BCB40907.1 cytochrome c | 35 | 12 | 34.3 | 8.3 |
|  | BCB44123.1 peptidase S8 | 110 | 22 | 20.0 | 9.9 |
| FlgJ1 | BCB41601.1 peptidoglycan hydrolase FlgJ | 307 | 307 | 100.0 | 0 |
|  | BCB44165.1 flagellar protein FlgJ | 95 | 33 | 34.7 | 8.26E-10 |
|  | BCB41083.1 UDP-N-acetyl-d-glucosamine 6-dehydrogenase WbpA | 67 | 20 | 29.9 | 0.22 |
|  | BCB42919.1 cytochrome c biogenesis ATP-binding export protein CcmA | 52 | 16 | 30.8 | 1.2 |
|  | BCB43534.1 maturase | 46 | 17 | 37.0 | 1.6 |
|  | BCB42066.1 hypothetical protein Vag1382_11920 | 61 | 20 | 32.8 | 1.8 |
|  | BCB42093.1 hypothetical protein Vag1382_12190 | 87 | 21 | 24.1 | 1.8 |
|  | BCB42450.1 threonine transporter RhtB | 26 | 10 | 38.5 | 3.5 |
|  | BCB43251.1 bifunctional diguanylate cyclase/phosphodiesterase | 33 | 12 | 36.4 | 5.7 |
|  | BCB44863.1 FHA domain-containing protein | 66 | 17 | 25.8 | 5.7 |
|  | BCB41052.1 membrane protein | 54 | 18 | 33.3 | 7.2 |
|  | BCB43144.1 hypothetical protein Vag1382_22710 | 23 | 10 | 43.5 | 9.1 |
| FlgK1 | BCB41602.1 flagellar hook protein FlgK | 646 | 646 | 100.0 | 0 |
|  | BCB44166.1 flagellar hook-associated protein 1 FlgK | 325 | 78 | 24.0 | 4.16E-20 |
|  | BCB44166.1 flagellar hook-associated protein 1 FlgK | 40 | 20 | 50.0 | 1.98E-05 |
|  | BCB44447.1 type VI secretion system-associated protein | 61 | 20 | 32.8 | 1.6 |
|  | BCB42407.1 sensor histidine kinase | 43 | 14 | 32.6 | 1.8 |
|  | BCB41551.1 hypothetical protein Vag1382_06770 | 59 | 17 | 28.8 | 3.7 |
|  | BCB41596.1 flagellar hook protein FlgE | 20 | 12 | 60.0 | 4.3 |
|  | BCB45215.1 methyl-accepting chemotaxis protein | 58 | 19 | 32.8 | 8.4 |
|  | BCB41246.1 phosphate transporter | 41 | 11 | 26.8 | 9.3 |
|  | BCB43065.1 cysteine desulfurase | 58 | 22 | 37.9 | 9.9 |
| FlgL1 | BCB41603.1 flagellar hook-associated protein FlgL | 397 | 397 | 100.0 | 0 |
|  | BCB44167.1 flagellar hook-associated protein 3 FlgL | 213 | 53 | 24.9 | 4.03E-15 |
|  | BCB44167.1 flagellar hook-associated protein 3 FlgL | 69 | 23 | 33.3 | 4.32E-05 |
|  | BCB45292.1 lateral flagellin LafA | 143 | 35 | 24.5 | 0.044 |
|  | BCB41834.1 DNA topoisomerase 1 | 61 | 15 | 24.6 | 0.32 |
|  | BCB43919.1 membrane protein insertase YidC | 68 | 20 | 29.4 | 0.79 |
|  | BCB45121.1 heme utilization protein HutZ | 98 | 24 | 24.5 | 1.3 |
|  | BCB41976.1 hypothetical protein Vag1382_11020 | 127 | 25 | 19.7 | 1.6 |
|  | BCB44568.1 type I secretion C-terminal target domain-containing protein | 171 | 41 | 24.0 | 1.6 |
|  | BCB41488.1 phosphoribosylformylglycinamidine synthase | 97 | 22 | 22.7 | 2.9 |
|  | BCB41307.1 glutamate synthase large subunit | 30 | 13 | 43.3 | 3 |
|  | BCB42501.1 GGDEF domain-containing protein | 66 | 22 | 33.3 | 4.3 |
|  | BCB41777.1 zinc/cadmium/mercury/lead-transporting ATPase | 84 | 22 | 26.2 | 6.4 |
|  | BCB41605.1 polar flagellin C | 123 | 30 | 24.4 | 8.5 |
|  | BCB45083.1 hemolysin D | 26 | 10 | 38.5 | 8.8 |
| FlaC | BCB41605.1 polar flagellin C | 384 | 384 | 100.0 | 0 |
|  | BCB42953.1 polar flagellin A | 384 | 249 | 64.8 | 3.73E-173 |
|  | BCB41606.1 flagellin D | 383 | 255 | 66.6 | 3.15E-172 |
|  | BCB42954.1 flagellin B | 383 | 254 | 66.3 | 1.37E-171 |
|  | BCB42955.1 polar flagellin F | 384 | 247 | 64.3 | 1.51E-170 |
|  | BCB41607.1 polar flagellin E | 381 | 173 | 45.4 | 1.01E-108 |
|  | BCB45292.1 lateral flagellin LafA | 384 | 132 | 34.4 | 5.44E-55 |
|  | BCB41437.1 GMP synthase [glutamine-hydrolyzing] | 52 | 20 | 38.5 | 1.7 |
| FlaD | BCB41606.1 flagellin D | 377 | 377 | 100.0 | 0 |
|  | BCB42954.1 flagellin B | 377 | 376 | 99.7 | 0 |
|  | BCB42953.1 polar flagellin A | 377 | 290 | 76.9 | 0 |
|  | BCB42955.1 polar flagellin F | 377 | 263 | 69.8 | 0 |
|  | BCB41605.1 polar flagellin C | 383 | 256 | 66.8 | 1.80E-180 |
|  | BCB41607.1 polar flagellin E | 375 | 200 | 53.3 | 2.29E-134 |
|  | BCB45292.1 lateral flagellin LafA | 171 | 90 | 52.6 | 2.86E-48 |
|  | BCB45292.1 lateral flagellin LafA | 79 | 36 | 45.6 | 6.59E-17 |
|  | BCB45296.1 lateral flagellar hook-associated protein 2 | 125 | 34 | 27.2 | 0.26 |
|  | BCB44167.1 flagellar hook-associated protein 3 FlgL | 53 | 16 | 30.2 | 0.97 |
|  | BCB43233.1 type I restriction-modification system subunit M | 44 | 16 | 36.4 | 2.5 |
|  | BCB40997.1 3'(2') 5'-bisphosphate nucleotidase CysQ | 49 | 16 | 32.7 | 2.9 |
|  | BCB44176.1 60 kDa chaperonin 2 | 28 | 15 | 53.6 | 2.9 |
|  | BCB43229.1 hypothetical protein Vag1382_23560 | 49 | 14 | 28.6 | 4.4 |
|  | BCB44040.1 putative pseudouridine methyltransferase | 32 | 11 | 34.4 | 6.5 |
|  | BCB44049.1 DNA-binding response regulator | 53 | 17 | 32.1 | 7.2 |
|  | BCB41218.1 dihydroxyacetone kinase subunit DhaK | 25 | 12 | 48.0 | 9.1 |
| FlaE | BCB41607.1 polar flagellin E | 374 | 374 | 100.0 | 0 |
|  | BCB42954.1 flagellin B | 375 | 200 | 53.3 | 9.88E-129 |
|  | BCB41606.1 flagellin D | 375 | 200 | 53.3 | 1.60E-128 |
|  | BCB42953.1 polar flagellin A | 374 | 186 | 49.7 | 1.01E-125 |
|  | BCB42955.1 polar flagellin F | 374 | 177 | 47.3 | 7.83E-119 |
|  | BCB41605.1 polar flagellin C | 381 | 173 | 45.4 | 9.85E-109 |
|  | BCB45292.1 lateral flagellin LafA | 153 | 63 | 41.2 | 2.50E-29 |
|  | BCB45292.1 lateral flagellin LafA | 76 | 26 | 34.2 | 2.60E-09 |
|  | BCB41151.1 sulfate adenylyltransferase subunit 1 | 84 | 24 | 28.6 | 0.64 |
|  | BCB42591.1 hypothetical protein Vag1382_17170 | 181 | 42 | 23.2 | 1.7 |
|  | BCB41973.1 ribosomal large subunit pseudouridine synthase B | 93 | 22 | 23.7 | 2.9 |
|  | BCB44131.1 2-aminoethylphosphonate--pyruvate transaminase | 91 | 25 | 27.5 | 3.4 |
|  | BCB41242.1 general secretion pathway protein GspA | 95 | 25 | 26.3 | 4.7 |
|  | BCB41882.1 multidrug efflux RND transporter permease subunit | 73 | 26 | 35.6 | 5.3 |
|  | BCB44029.1 trimethylamine N-oxide reductase I catalytic subunit | 50 | 17 | 34.0 | 5.7 |
|  | BCB44167.1 flagellar hook-associated protein 3 FlgL | 94 | 23 | 24.5 | 5.9 |
|  | BCB41672.1 arginine--tRNA ligase | 47 | 15 | 31.9 | 7.3 |
| CheW | BCB42920.1 chemotaxis protein CheW | 164 | 164 | 100.0 | 3.78E-118 |
|  | BCB41591.1 chemotaxis protein CheW | 138 | 41 | 29.7 | 1.98E-13 |
|  | BCB42751.1 chemotaxis protein CheW | 142 | 42 | 29.6 | 5.32E-11 |
|  | BCB45119.1 chemotaxis protein CheW | 137 | 35 | 25.5 | 3.28E-10 |
|  | BCB44611.1 chemotaxis protein CheV | 146 | 33 | 22.6 | 3.78E-10 |
|  | BCB43629.1 glutathione-regulated potassium-efflux system protein KefB | 98 | 22 | 22.4 | 0.7 |
|  | BCB41743.1 2-succinylbenzoate-CoA ligase | 55 | 19 | 34.5 | 1.6 |
|  | BCB42022.1 cyclic nucleotide-binding protein | 145 | 40 | 27.6 | 2.3 |
|  | BCB42921.1 chemotaxis protein CheW | 80 | 18 | 22.5 | 3.8 |
|  | BCB44685.1 LysR family transcriptional regulator | 30 | 10 | 33.3 | 4.5 |
|  | BCB42686.1 antimicrobial peptide ABC transporter permease SapB | 54 | 15 | 27.8 | 5.2 |
|  | BCB42921.1 chemotaxis protein CheW | 350 | 350 | 100.0 | 0 |
|  | BCB44452.1 transcriptional regulator | 33 | 11 | 33.3 | 0.41 |
|  | BCB41307.1 glutamate synthase large subunit | 114 | 30 | 26.3 | 0.44 |
|  | BCB41309.1 glutamate synthase | 56 | 13 | 23.2 | 0.59 |
|  | BCB42703.1 nucleoside-diphosphate sugar epimerase | 77 | 22 | 28.6 | 0.93 |
|  | BCB42736.1 paraquat-inducible protein B | 62 | 17 | 27.4 | 1.4 |
|  | BCB44231.1 multidrug resistance protein | 175 | 44 | 25.1 | 1.6 |
|  | BCB41712.1 oxidoreductase | 114 | 27 | 23.7 | 2.1 |
|  | BCB41207.1 acetolactate synthase | 68 | 17 | 25.0 | 3.6 |
|  | BCB45431.1 transcriptional regulator | 99 | 31 | 31.3 | 4.4 |
|  | BCB41715.1 RNA helicase | 33 | 15 | 45.5 | 4.8 |
|  | BCB43300.1 serine protease | 40 | 12 | 30.0 | 6.3 |
|  | BCB43907.1 hypothetical protein Vag1382_30340 | 20 | 12 | 60.0 | 7.6 |
|  | BCB42923.1 chemotaxis response regulator protein-glutamate methylesterase of group 1 operon CheB | 370 | 370 | 100.0 | 0 |
| CheB | BCB42017.1 DNA-binding response regulator | 107 | 41 | 38.3 | 3.27E-14 |
|  | BCB42881.1 two-component system response regulator | 131 | 40 | 30.5 | 1.33E-10 |
|  | BCB44892.1 DNA-binding response regulator | 114 | 44 | 38.6 | 2.99E-10 |
|  | BCB43366.1 histidine kinase | 113 | 38 | 33.6 | 6.42E-10 |
|  | BCB43942.1 putative response regulatory protein | 106 | 31 | 29.2 | 1.83E-09 |
|  | BCB41363.1 putative response regulatory protein | 101 | 31 | 30.7 | 5.20E-09 |
|  | BCB45455.1 DNA-binding response regulator | 130 | 43 | 33.1 | 4.70E-08 |
|  | BCB42502.1 two-component system sensor histidine kinase/response regulator | 175 | 52 | 29.7 | 5.77E-08 |
|  | BCB42502.1 two-component system sensor histidine kinase/response regulator | 111 | 35 | 31.5 | 3.92E-04 |
|  | BCB44604.1 hybrid sensor histidine kinase/response regulator | 107 | 36 | 33.6 | 6.38E-08 |
|  | BCB42654.1 DNA-binding response regulator | 108 | 34 | 31.5 | 6.94E-08 |
|  | BCB44762.1 diguanylate cyclase response regulator | 155 | 40 | 25.8 | 1.18E-07 |
|  | BCB44611.1 chemotaxis protein CheV | 118 | 43 | 36.4 | 1.65E-07 |
|  | BCB45406.1 DNA-binding response regulator | 105 | 31 | 29.5 | 2.20E-07 |
|  | BCB42342.1 transcriptional regulator | 151 | 37 | 24.5 | 3.59E-07 |
|  | BCB40971.1 nitrogen regulation protein NR(I) | 106 | 29 | 27.4 | 2.27E-06 |
|  | BCB43692.1 DNA-binding response regulator | 108 | 31 | 28.7 | 2.51E-06 |
|  | BCB44049.1 DNA-binding response regulator | 105 | 27 | 25.7 | 3.00E-06 |
|  | BCB41978.1 DNA-binding response regulator | 104 | 30 | 28.8 | 4.79E-06 |
|  | BCB41942.1 DNA-binding response regulator | 91 | 31 | 34.1 | 5.52E-06 |
|  | BCB41957.1 hybrid sensor histidine kinase/response regulator | 88 | 35 | 39.8 | 7.69E-06 |
|  | BCB42814.1 regulatory protein LuxO | 80 | 23 | 28.8 | 2.44E-05 |
|  | BCB44537.1 hybrid sensor histidine kinase/response regulator | 77 | 30 | 39.0 | 3.27E-05 |
|  | BCB44791.1 DNA-binding response regulator | 105 | 31 | 29.5 | 3.98E-05 |
|  | BCB42285.1 hybrid sensor histidine kinase/response regulator | 110 | 39 | 35.5 | 5.10E-05 |
|  | BCB43282.1 histidine kinase | 118 | 31 | 26.3 | 7.09E-05 |
|  | BCB41843.1 two-component system response regulator TorR | 109 | 31 | 28.4 | 7.11E-05 |
|  | BCB45189.1 DNA-binding response regulator | 120 | 39 | 32.5 | 8.04E-05 |
|  | BCB42039.1 hybrid sensor histidine kinase/response regulator | 108 | 33 | 30.6 | 1.65E-04 |
|  | BCB44084.1 two-component system response regulator | 61 | 20 | 32.8 | 1.65E-04 |
|  | BCB44366.1 autoinducer 2 sensor kinase/phosphatase LuxQ | 129 | 43 | 33.3 | 1.83E-04 |
|  | BCB45119.1 chemotaxis protein CheW | 118 | 33 | 28.0 | 2.34E-04 |
|  | BCB44327.1 DNA-binding response regulator | 103 | 27 | 26.2 | 2.41E-04 |
|  | BCB44359.1 hybrid sensor histidine kinase/response regulator | 39 | 20 | 51.3 | 2.96E-04 |
|  | BCB44508.1 histidine kinase | 111 | 32 | 28.8 | 3.64E-04 |
|  | BCB41007.1 DNA-binding response regulator | 201 | 52 | 25.9 | 4.80E-04 |
|  | BCB44424.1 transcriptional regulator | 102 | 27 | 26.5 | 5.17E-04 |
|  | BCB42354.1 sigma-54-dependent Fis family transcriptional regulator | 107 | 29 | 27.1 | 0.001 |
|  | BCB42136.1 transcriptional regulatory protein | 106 | 26 | 24.5 | 0.001 |
|  | BCB42728.1 DNA-binding response regulator | 102 | 28 | 27.5 | 0.001 |
|  | BCB41591.1 chemotaxis protein CheW | 114 | 35 | 30.7 | 0.001 |
|  | BCB44868.1 hypothetical protein Vag1382_39950 | 106 | 30 | 28.3 | 0.001 |
|  | BCB44392.1 DNA-binding response regulator | 122 | 38 | 31.1 | 0.002 |
|  | BCB44348.1 DNA-binding response regulator | 103 | 27 | 26.2 | 0.003 |
|  | BCB42946.1 sigma-54-dependent Fis family transcriptional regulator FlaM | 106 | 27 | 25.5 | 0.003 |
|  | BCB41215.1 DNA-binding response regulator | 134 | 29 | 21.6 | 0.005 |
|  | BCB41878.1 sensor histidine kinase | 99 | 29 | 29.3 | 0.006 |
|  | BCB42926.1 response regulator | 84 | 26 | 31.0 | 0.007 |
|  | BCB41314.1 DNA-binding response regulator | 105 | 24 | 22.9 | 0.01 |
|  | BCB44610.1 DNA-binding response regulator | 104 | 24 | 23.1 | 0.013 |
|  | BCB43932.1 hybrid sensor histidine kinase/response regulator | 40 | 15 | 37.5 | 0.018 |
|  | BCB44754.1 DNA-binding response regulator | 61 | 17 | 27.9 | 0.031 |
|  | BCB44626.1 transcriptional regulatory protein | 104 | 25 | 24.0 | 0.034 |
|  | BCB42438.1 DNA-binding response regulator | 68 | 20 | 29.4 | 0.036 |
|  | BCB43700.1 DNA-binding response regulator | 121 | 26 | 21.5 | 0.12 |
|  | BCB42751.1 chemotaxis protein CheW | 76 | 24 | 31.6 | 0.12 |
|  | BCB45263.1 DNA-binding response regulator | 76 | 20 | 26.3 | 0.38 |
|  | BCB42325.1 sigma-54-dependent Fis family transcriptional regulator | 148 | 40 | 27.0 | 0.55 |
|  | BCB44575.1 histidine kinase | 113 | 31 | 27.4 | 1.4 |
|  | BCB40962.1 DNA polymerase I | 48 | 11 | 22.9 | 4.6 |
|  | BCB45230.1 GGDEF domain-containing protein | 44 | 13 | 29.5 | 7.7 |
|  | BCB43837.1 gamma carbonic anhydrase family protein | 25 | 11 | 44.0 | 8.5 |
|  | BCB41615.1 DNA ligase | 58 | 18 | 31.0 | 9.9 |
|  | BCB41312.1 aerobic respiration control sensor protein | 79 | 24 | 30.4 | 10 |
| CheA | BCB42924.1 chemotaxis protein CheA | 744 | 744 | 100.0 | 0 |
|  | BCB42324.1 two-component sensor histidine kinase | 72 | 22 | 30.6 | 1.16E-04 |
|  | BCB42622.1 transcriptional regulator | 54 | 17 | 31.5 | 0.002 |
|  | BCB41393.1 PAS domain-containing sensor histidine kinase | 101 | 27 | 26.7 | 0.003 |
|  | BCB43282.1 histidine kinase | 242 | 58 | 24.0 | 0.004 |
|  | BCB44662.1 ATPase | 59 | 20 | 33.9 | 0.005 |
|  | BCB44575.1 histidine kinase | 43 | 14 | 32.6 | 0.014 |
|  | BCB44575.1 histidine kinase | 23 | 10 | 43.5 | 5.7 |
|  | BCB41312.1 aerobic respiration control sensor protein | 55 | 20 | 36.4 | 0.019 |
|  | BCB42039.1 hybrid sensor histidine kinase/response regulator | 38 | 13 | 34.2 | 0.049 |
|  | BCB44366.1 autoinducer 2 sensor kinase/phosphatase LuxQ | 41 | 13 | 31.7 | 0.05 |
|  | BCB42947.1 sensor histidine kinase FlaL | 182 | 40 | 22.0 | 0.051 |
|  | BCB42135.1 histidine kinase | 142 | 32 | 22.5 | 0.053 |
|  | BCB44508.1 histidine kinase | 110 | 25 | 22.7 | 0.076 |
|  | BCB40972.1 two-component system sensor histidine kinase NtrB | 59 | 18 | 30.5 | 0.16 |
|  | BCB42502.1 two-component system sensor histidine kinase/response regulator | 97 | 23 | 23.7 | 0.3 |
|  | BCB44625.1 signal transduction histidine kinase | 56 | 18 | 32.1 | 0.32 |
|  | BCB44604.1 hybrid sensor histidine kinase/response regulator | 95 | 20 | 21.1 | 0.4 |
|  | BCB43932.1 hybrid sensor histidine kinase/response regulator | 40 | 12 | 30.0 | 0.69 |
|  | BCB42285.1 hybrid sensor histidine kinase/response regulator | 110 | 24 | 21.8 | 1.1 |
|  | BCB44050.1 two-component sensor histidine kinase | 93 | 24 | 25.8 | 1.1 |
|  | BCB43718.1 MFS transporter | 27 | 11 | 40.7 | 1.2 |
|  | BCB42655.1 sensor histidine kinase | 117 | 25 | 21.4 | 1.3 |
|  | BCB42016.1 sensor histidine kinase | 40 | 12 | 30.0 | 1.7 |
|  | BCB44537.1 hybrid sensor histidine kinase/response regulator | 38 | 11 | 28.9 | 2.4 |
|  | BCB42200.1 bordetella uptake gene family protein | 139 | 40 | 28.8 | 2.4 |
|  | BCB41878.1 sensor histidine kinase | 39 | 12 | 30.8 | 2.6 |
|  | BCB43397.1 phosphoglycerate kinase | 63 | 19 | 30.2 | 3.4 |
|  | BCB43760.1 LexA repressor | 50 | 16 | 32.0 | 4.6 |
|  | BCB43707.1 sensor histidine kinase | 156 | 34 | 21.8 | 4.6 |
|  | BCB43693.1 two-component sensor histidine kinase | 57 | 15 | 26.3 | 5.6 |
|  | BCB43888.1 FMN-binding protein MioC | 79 | 21 | 26.6 | 6.1 |
|  | BCB44453.1 type VI secretion protein IcmF | 69 | 22 | 31.9 | 8.8 |
| CheZ | BCB42925.1 protein phosphatase CheZ | 246 | 246 | 100.0 | 0 |
|  | BCB43448.1 hypothetical protein Vag1382_25750 | 127 | 31 | 24.4 | 0.38 |
|  | BCB40972.1 two-component system sensor histidine kinase NtrB | 113 | 32 | 28.3 | 3.7 |
|  | BCB41976.1 hypothetical protein Vag1382_11020 | 34 | 9 | 26.5 | 4.9 |
|  | BCB41387.1 penicillin-insensitive murein endopeptidase | 19 | 9 | 47.4 | 7 |
|  | BCB42219.1 outer membrane protein | 70 | 17 | 24.3 | 8.7 |
|  | BCB45414.1 hypothetical protein Vag1382_45410 | 19 | 10 | 52.6 | 9.2 |
|  | BCB41738.1 menaquinone-specific isochorismate synthase | 28 | 10 | 35.7 | 9.6 |
|  | BCB41920.1 tRNA-specific 2-thiouridylase MnmA | 34 | 14 | 41.2 | 10 |
| CheY | BCB42926.1 response regulator | 126 | 126 | 100.0 | 4.70E-91 |
|  | BCB41392.1 DNA-binding response regulator | 123 | 38 | 30.9 | 1.62E-18 |
|  | BCB43932.1 hybrid sensor histidine kinase/response regulator | 121 | 39 | 32.2 | 7.17E-13 |
|  | BCB42946.1 sigma-54-dependent Fis family transcriptional regulator FlaM | 110 | 42 | 38.2 | 7.94E-13 |
|  | BCB41007.1 DNA-binding response regulator | 125 | 35 | 28.0 | 1.19E-12 |
|  | BCB42502.1 two-component system sensor histidine kinase/response regulator | 120 | 40 | 33.3 | 7.76E-12 |
|  | BCB42502.1 two-component system sensor histidine kinase/response regulator | 111 | 33 | 29.7 | 4.82E-09 |
|  | BCB44604.1 hybrid sensor histidine kinase/response regulator | 114 | 33 | 28.9 | 9.44E-12 |
|  | BCB43282.1 histidine kinase | 113 | 30 | 26.5 | 1.41E-11 |
|  | BCB41215.1 DNA-binding response regulator | 119 | 34 | 28.6 | 3.54E-11 |
|  | BCB44049.1 DNA-binding response regulator | 121 | 34 | 28.1 | 3.93E-11 |
|  | BCB44892.1 DNA-binding response regulator | 121 | 38 | 31.4 | 4.35E-11 |
|  | BCB40971.1 nitrogen regulation protein NR(I) | 104 | 34 | 32.7 | 5.15E-11 |
|  | BCB44762.1 diguanylate cyclase response regulator | 116 | 36 | 31.0 | 1.35E-10 |
|  | BCB45406.1 DNA-binding response regulator | 123 | 33 | 26.8 | 2.09E-10 |
|  | BCB42881.1 two-component system response regulator | 119 | 31 | 26.1 | 2.13E-10 |
|  | BCB41978.1 DNA-binding response regulator | 106 | 31 | 29.2 | 2.87E-10 |
|  | BCB44508.1 histidine kinase | 121 | 34 | 28.1 | 3.14E-10 |
|  | BCB43692.1 DNA-binding response regulator | 119 | 36 | 30.3 | 3.37E-10 |
|  | BCB42342.1 transcriptional regulator | 124 | 33 | 26.6 | 7.27E-10 |
|  | BCB45263.1 DNA-binding response regulator | 120 | 31 | 25.8 | 1.26E-09 |
|  | BCB44611.1 chemotaxis protein CheV | 115 | 33 | 28.7 | 1.50E-09 |
|  | BCB44868.1 hypothetical protein Vag1382_39950 | 121 | 29 | 24.0 | 1.63E-09 |
|  | BCB44575.1 histidine kinase | 126 | 33 | 26.2 | 1.77E-09 |
|  | BCB42654.1 DNA-binding response regulator | 106 | 33 | 31.1 | 1.86E-09 |
|  | BCB41724.1 sigma-54-dependent Fis family transcriptional regulator | 91 | 32 | 35.2 | 4.01E-09 |
|  | BCB44366.1 autoinducer 2 sensor kinase/phosphatase LuxQ | 115 | 34 | 29.6 | 5.28E-09 |
|  | BCB41942.1 DNA-binding response regulator | 91 | 29 | 31.9 | 5.55E-09 |
|  | BCB41843.1 two-component system response regulator TorR | 123 | 28 | 22.8 | 6.66E-09 |
|  | BCB42039.1 hybrid sensor histidine kinase/response regulator | 119 | 33 | 27.7 | 7.95E-09 |
|  | BCB44537.1 hybrid sensor histidine kinase/response regulator | 123 | 32 | 26.0 | 9.35E-09 |
|  | BCB42285.1 hybrid sensor histidine kinase/response regulator | 109 | 35 | 32.1 | 1.23E-08 |
|  | BCB43366.1 histidine kinase | 115 | 30 | 26.1 | 1.32E-08 |
|  | BCB42325.1 sigma-54-dependent Fis family transcriptional regulator | 119 | 34 | 28.6 | 2.12E-08 |
|  | BCB41957.1 hybrid sensor histidine kinase/response regulator | 124 | 32 | 25.8 | 2.27E-08 |
|  | BCB42017.1 DNA-binding response regulator | 120 | 38 | 31.7 | 3.08E-08 |
|  | BCB42751.1 chemotaxis protein CheW | 90 | 32 | 35.6 | 3.83E-08 |
|  | BCB41878.1 sensor histidine kinase | 118 | 34 | 28.8 | 3.91E-08 |
|  | BCB44392.1 DNA-binding response regulator | 106 | 35 | 33.0 | 5.38E-08 |
|  | BCB41314.1 DNA-binding response regulator | 120 | 32 | 26.7 | 5.58E-08 |
|  | BCB42406.1 response regulator | 125 | 30 | 24.0 | 3.94E-07 |
|  | BCB45119.1 chemotaxis protein CheW | 122 | 35 | 28.7 | 5.05E-07 |
|  | BCB44359.1 hybrid sensor histidine kinase/response regulator | 125 | 35 | 28.0 | 6.47E-07 |
|  | BCB44084.1 two-component system response regulator | 123 | 31 | 25.2 | 1.72E-06 |
|  | BCB42438.1 DNA-binding response regulator | 122 | 32 | 26.2 | 1.74E-06 |
|  | BCB42814.1 regulatory protein LuxO | 114 | 27 | 23.7 | 2.78E-06 |
|  | BCB44348.1 DNA-binding response regulator | 119 | 30 | 25.2 | 3.68E-06 |
|  | BCB45189.1 DNA-binding response regulator | 112 | 30 | 26.8 | 4.91E-06 |
|  | BCB45455.1 DNA-binding response regulator | 123 | 28 | 22.8 | 6.15E-06 |
|  | BCB41591.1 chemotaxis protein CheW | 111 | 32 | 28.8 | 7.55E-06 |
|  | BCB41312.1 aerobic respiration control sensor protein | 123 | 34 | 27.6 | 1.74E-05 |
|  | BCB43700.1 DNA-binding response regulator | 73 | 21 | 28.8 | 1.14E-04 |
|  | BCB44754.1 DNA-binding response regulator | 73 | 21 | 28.8 | 1.38E-04 |
|  | BCB42354.1 sigma-54-dependent Fis family transcriptional regulator | 113 | 25 | 22.1 | 1.99E-04 |
|  | BCB44327.1 DNA-binding response regulator | 120 | 28 | 23.3 | 3.25E-04 |
|  | BCB44663.1 two-component system response regulator | 63 | 19 | 30.2 | 5.43E-04 |
|  | BCB44424.1 transcriptional regulator | 121 | 29 | 24.0 | 6.46E-04 |
|  | BCB44607.1 diguanylate phosphodiesterase | 126 | 29 | 23.0 | 8.10E-04 |
|  | BCB42728.1 DNA-binding response regulator | 117 | 28 | 23.9 | 0.002 |
|  | BCB42923.1 chemotaxis response regulator protein-glutamate methylesterase of group 1 operon CheB | 84 | 26 | 31.0 | 0.003 |
|  | BCB42136.1 transcriptional regulatory protein | 71 | 20 | 28.2 | 0.007 |
|  | BCB44626.1 transcriptional regulatory protein | 70 | 19 | 27.1 | 0.045 |
|  | BCB43172.1 acetoacetate metabolism regulatory protein AtoC | 120 | 28 | 23.3 | 0.16 |
|  | BCB44058.1 hypothetical protein Vag1382_31850 | 96 | 27 | 28.1 | 0.18 |
|  | BCB44568.1 type I secretion C-terminal target domain-containing protein | 56 | 20 | 35.7 | 0.27 |
|  | BCB41363.1 putative response regulatory protein | 119 | 30 | 25.2 | 0.29 |
|  | BCB44526.1 glycine/betaine ABC transporter substrate-binding protein | 59 | 15 | 25.4 | 0.61 |
|  | BCB41346.1 UPF0721 transmembrane protein | 27 | 11 | 40.7 | 0.86 |
|  | BCB42939.1 flagellar hook-length control protein FliK | 42 | 16 | 38.1 | 1.2 |
|  | BCB44038.1 TldD protein | 35 | 14 | 40.0 | 1.3 |
|  | BCB43867.1 branched chain amino acid aminotransferase | 62 | 18 | 29.0 | 2 |
|  | BCB41487.1 membrane-bound lytic murein transglycosylase F | 17 | 9 | 52.9 | 5.5 |
|  | BCB40962.1 DNA polymerase I | 38 | 10 | 26.3 | 6.1 |
|  | BCB44406.1 transketolase 2 | 28 | 9 | 32.1 | 6.3 |
|  | BCB43319.1 pyruvate dehydrogenase E1 component | 38 | 11 | 28.9 | 7 |
|  | BCB42761.1 deoxyribonuclease | 33 | 11 | 33.3 | 7.1 |
|  | BCB41091.1 glycosyl transferase | 39 | 13 | 33.3 | 7.2 |
|  | BCB44697.1 Zn-dependent protease | 68 | 16 | 23.5 | 8.9 |
| FliA1 | BCB42927.1 RNA polymerase sigma factor FliA | 244 | 244 | 100.0 | 0 |
|  | BCB45301.1 RNA polymerase sigma factor for flagellar operon | 224 | 68 | 30.4 | 3.20E-35 |
|  | BCB41231.1 RNA polymerase sigma factor RpoD | 231 | 61 | 26.4 | 1.09E-12 |
|  | BCB43353.1 RNA polymerase sigma factor RpoS | 239 | 58 | 24.3 | 4.32E-11 |
|  | BCB43767.1 RNA polymerase sigma factor RpoH | 216 | 50 | 23.1 | 0.042 |
|  | BCB43059.1 RNA polymerase sigma factor | 84 | 21 | 25.0 | 0.94 |
|  | BCB43686.1 60 kDa chaperonin 1 | 53 | 14 | 26.4 | 1.6 |
|  | BCB42584.1 imidazolonepropionase | 87 | 23 | 26.4 | 3.2 |
|  | BCB43606.1 phosphoenolpyruvate carboxylase | 34 | 11 | 32.4 | 4.1 |
|  | BCB44037.1 hydroxymethylpyrimidine/phosphomethylpyrimidine kinase | 55 | 16 | 29.1 | 5.4 |
|  | BCB42446.1 hypothetical protein Vag1382_15720 | 64 | 21 | 32.8 | 5.9 |
|  | BCB44715.1 UPF0271 protein | 31 | 12 | 38.7 | 6.9 |
|  | BCB43264.1 preprotein translocase subunit SecG | 31 | 13 | 41.9 | 8.7 |
|  | BCB43000.1 ribonuclease HII | 52 | 16 | 30.8 | 9.3 |
| FlhG | BCB42928.1 site-determining protein FlhG | 295 | 295 | 100.0 | 0 |
|  | BCB41685.1 site-determining protein | 168 | 54 | 32.1 | 1.13E-15 |
|  | BCB42782.1 iron-sulfur cluster carrier protein | 260 | 70 | 26.9 | 9.43E-10 |
|  | BCB43885.1 cobyric acid synthase CobQ | 168 | 42 | 25.0 | 5.60E-06 |
|  | BCB45351.1 tyrosine protein kinase | 71 | 19 | 26.8 | 7.16E-04 |
|  | BCB42922.1 cobyric acid synthase | 225 | 53 | 23.6 | 0.001 |
|  | BCB43113.1 Flp pilus assembly protein | 171 | 36 | 21.1 | 0.007 |
|  | BCB45474.1 chromosome partitioning protein ParA | 173 | 39 | 22.5 | 0.029 |
|  | BCB42658.1 bifunctional metallophosphatase/5'-nucleotidase | 165 | 39 | 23.6 | 0.68 |
|  | BCB44845.1 type VI secretion protein | 39 | 17 | 43.6 | 0.91 |
|  | BCB43770.1 signal recognition particle receptor FtsY | 76 | 24 | 31.6 | 1.3 |
|  | BCB41963.1 tryptophan synthase beta chain 1 | 94 | 23 | 24.5 | 1.3 |
|  | BCB45393.1 ferric citrate ABC transporter ATP-binding protein FecE | 40 | 15 | 37.5 | 4 |
|  | BCB43460.1 hypothetical protein Vag1382_25870 | 12 | 9 | 75.0 | 4.9 |
|  | BCB41444.1 plasmid replication protein | 62 | 17 | 27.4 | 5.2 |
|  | BCB41112.1 primosomal protein N' | 34 | 13 | 38.2 | 6.9 |
|  | BCB44736.1 hypothetical protein Vag1382_38630 | 64 | 15 | 23.4 | 9.4 |
|  | BCB44726.1 hypothetical protein Vag1382_38530 | 64 | 15 | 23.4 | 9.4 |
|  | BCB41756.1 hypothetical protein Vag1382_08820 | 27 | 12 | 44.4 | 9.4 |
| FlhF | BCB42929.1 flagellar biosynthesis regulator FlhF | 495 | 495 | 100.0 | 0 |
|  | BCB43770.1 signal recognition particle receptor FtsY | 191 | 61 | 31.9 | 3.71E-16 |
|  | BCB43334.1 signal recognition particle protein | 184 | 47 | 25.5 | 9.53E-06 |
|  | BCB42458.1 putative ribosome biogenesis GTPase RsgA | 36 | 15 | 41.7 | 0.008 |
|  | BCB43312.1 ABC transporter ATP-binding protein | 44 | 16 | 36.4 | 0.04 |
|  | BCB42663.1 ABC transporter ATP-binding protein | 32 | 11 | 34.4 | 0.052 |
|  | BCB43293.1 iron(III) ABC transporter ATP-binding protein | 43 | 15 | 34.9 | 0.077 |
|  | BCB45076.1 ABC transporter ATP-binding protein | 45 | 15 | 33.3 | 0.13 |
|  | BCB41174.1 thiamine import ATP-binding protein ThiQ | 26 | 11 | 42.3 | 0.17 |
|  | BCB45013.1 ABC transporter ATP-binding protein | 26 | 12 | 46.2 | 0.31 |
|  | BCB45234.1 putative ABC transporter ATP-binding protein | 83 | 23 | 27.7 | 0.31 |
|  | BCB41375.1 energy-dependent translational throttle protein EttA | 63 | 15 | 23.8 | 0.33 |
|  | BCB42253.1 ABC transporter ATPase | 48 | 15 | 31.3 | 0.35 |
|  | BCB42302.1 spermidine/putrescine import ATP-binding protein PotA | 43 | 15 | 34.9 | 0.43 |
|  | BCB41158.1 ABC transporter ATP-binding protein | 57 | 18 | 31.6 | 0.48 |
|  | BCB44848.1 ClpV1 family T6SS ATPase | 43 | 15 | 34.9 | 0.55 |
|  | BCB43490.1 ABC transporter ATP-binding protein | 30 | 12 | 40.0 | 0.56 |
|  | BCB44133.1 ABC transporter ATP-binding protein | 26 | 11 | 42.3 | 0.6 |
|  | BCB45129.1 hemin import ATP-binding protein HmuV | 39 | 18 | 46.2 | 0.62 |
|  | BCB43745.1 pantothenate kinase | 40 | 15 | 37.5 | 0.8 |
|  | BCB43646.1 23S rRNA (guanosine-2'-O-)-methyltransferase RlmB | 88 | 23 | 26.1 | 0.82 |
|  | BCB44438.1 ClpV1 family T6SS ATPase | 59 | 17 | 28.8 | 0.85 |
|  | BCB41560.1 ribose-phosphate pyrophosphokinase | 105 | 28 | 26.7 | 0.9 |
|  | BCB43411.1 twitching motility protein PilT | 21 | 11 | 52.4 | 0.9 |
|  | BCB44954.1 ABC transporter ATP-binding protein | 18 | 11 | 61.1 | 0.95 |
|  | BCB43495.1 ABC transporter ATP-binding protein | 17 | 11 | 64.7 | 0.96 |
|  | BCB42532.1 polyamine-transporting ATPase | 23 | 12 | 52.2 | 1 |
|  | BCB44650.1 ABC transporter ATP-binding protein | 18 | 11 | 61.1 | 1 |
|  | BCB41728.1 ATP-dependent Clp protease ATP-binding subunit ClpX | 50 | 15 | 30.0 | 1 |
|  | BCB44236.1 ABC transporter ATP-binding protein | 33 | 12 | 36.4 | 1.3 |
|  | BCB41729.1 Lon protease | 185 | 43 | 23.2 | 1.4 |
|  | BCB42323.1 ABC-F family ATPase | 27 | 13 | 48.1 | 1.5 |
|  | BCB43162.1 chaperone protein ClpB | 21 | 12 | 57.1 | 1.5 |
|  | BCB45248.1 ABC transporter ATP-binding protein | 45 | 14 | 31.1 | 1.7 |
|  | BCB41385.1 chaperone protein ClpB | 22 | 12 | 54.5 | 1.8 |
|  | BCB41665.1 zinc import ATP-binding protein ZnuC | 21 | 10 | 47.6 | 1.8 |
|  | BCB42386.1 multidrug ABC transporter ATP-binding protein | 44 | 16 | 36.4 | 2 |
|  | BCB43410.1 twitching motility protein PilT | 39 | 14 | 35.9 | 2.1 |
|  | BCB44488.1 ABC transporter ATP-binding protein | 51 | 16 | 31.4 | 2.4 |
|  | BCB41685.1 site-determining protein | 64 | 23 | 35.9 | 2.6 |
|  | BCB41827.1 ATP-dependent Clp protease ATP-binding subunit ClpA | 59 | 18 | 30.5 | 2.6 |
|  | BCB41107.1 ATP-dependent protease ATPase subunit HslU | 22 | 11 | 50.0 | 2.6 |
|  | BCB43876.1 ATP synthase subunit beta | 37 | 12 | 32.4 | 2.6 |
|  | BCB44764.1 molybdenum import ATP-binding protein ModC | 33 | 13 | 39.4 | 2.8 |
|  | BCB42228.1 arginine ABC transporter ATP-binding protein | 21 | 9 | 42.9 | 3 |
|  | BCB41803.1 PrkA family serine protein kinase | 29 | 12 | 41.4 | 3.1 |
|  | BCB43526.1 MSHA biogenesis protein MshE | 17 | 10 | 58.8 | 3.1 |
|  | BCB43589.1 shikimate kinase | 26 | 11 | 42.3 | 3.2 |
|  | BCB40987.1 type II secretion system protein GspE | 17 | 9 | 52.9 | 4.2 |
|  | BCB45393.1 ferric citrate ABC transporter ATP-binding protein FecE | 25 | 10 | 40.0 | 4.2 |
|  | BCB41399.1 phosphate import ATP-binding protein PstB 1 | 19 | 9 | 47.4 | 4.5 |
|  | BCB43187.1 cobalamin synthase | 19 | 9 | 47.4 | 4.7 |
|  | BCB44562.1 iron(III) ABC transporter ATP-binding protein | 27 | 10 | 37.0 | 5.1 |
|  | BCB42815.1 UvrABC system protein B | 21 | 12 | 57.1 | 5.6 |
|  | BCB45369.1 putative ribosome biogenesis GTPase RsgA 2 | 21 | 11 | 52.4 | 5.9 |
|  | BCB40877.1 DNA replication and repair protein RecF | 28 | 12 | 42.9 | 6.9 |
|  | BCB42550.1 vitamin B12 import ATP-binding protein BtuD | 32 | 11 | 34.4 | 7.3 |
|  | BCB45211.1 phosphate import ATP-binding protein PstB 2 | 49 | 16 | 32.7 | 7.9 |
|  | BCB44254.1 phosphoenolpyruvate synthase | 51 | 18 | 35.3 | 8.2 |
|  | BCB44219.1 elongation factor G | 18 | 10 | 55.6 | 8.6 |
| FlhA1 | BCB42930.1 flagellar biosynthesis protein FlhA | 710 | 710 | 100.0 | 0 |
|  | BCB45290.1 flagellar biosynthesis protein FlhA | 697 | 351 | 50.4 | 0 |
|  | BCB42187.1 EscV/YscV/HrcV family type III secretion system export apparatus protein | 709 | 251 | 35.4 | 1.97E-131 |
|  | BCB44678.1 DEAD/DEAH box helicase | 55 | 17 | 30.9 | 0.56 |
|  | BCB43702.1 hypothetical protein Vag1382_28290 | 36 | 12 | 33.3 | 2.3 |
|  | BCB41878.1 sensor histidine kinase | 124 | 38 | 30.6 | 3.1 |
|  | BCB44748.1 LysR family transcriptional regulator | 21 | 9 | 42.9 | 8.7 |
| FlhB1 | BCB42931.1 flagellar biosynthesis protein FlhB | 376 | 376 | 100.0 | 0 |
|  | BCB45289.1 flagellar biosynthesis protein FlhB | 368 | 143 | 38.9 | 2.87E-82 |
|  | BCB42174.1 EscU/YscU/HrcU family type III secretion system export apparatus switch protein | 351 | 113 | 32.2 | 2.92E-54 |
|  | BCB45394.1 Fe3+ dicitrate ABC transporter permease | 63 | 20 | 31.7 | 4.7 |
|  | BCB42295.1 cytochrome biogenesis protein | 83 | 26 | 31.3 | 5.1 |
|  | BCB45084.1 cation transporter | 66 | 20 | 30.3 | 5.2 |
| FliR1 | BCB42932.1 flagellar biosynthetic protein FliR | 260 | 260 | 100.0 | 0 |
|  | BCB45288.1 flagellar biosynthetic protein FliR | 230 | 79 | 34.3 | 4.38E-40 |
|  | BCB42175.1 EscT/YscT/HrcT family type III secretion system export apparatus protein | 197 | 50 | 25.4 | 2.53E-04 |
|  | BCB42384.1 V10 pilin | 61 | 16 | 26.2 | 1.2 |
|  | BCB44806.1 anaerobic C4-dicarboxylate transporter | 76 | 20 | 26.3 | 6.5 |
|  | BCB43085.1 sn-glycerol-3-phosphate transporter | 96 | 30 | 31.3 | 7.2 |
|  | BCB41713.1 hypothetical protein Vag1382_08390 | 11 | 6 | 54.5 | 8 |
| FliQ1 | BCB42933.1 flagellar export apparatus protein FliQ | 89 | 89 | 100.0 | 4.72E-60 |
|  | BCB45287.1 flagellar export apparatus protein FliQ | 85 | 46 | 54.1 | 7.81E-31 |
|  | BCB42176.1 EscS/YscS/HrcS family type III secretion system export apparatus protein | 76 | 23 | 30.3 | 1.58E-07 |
|  | BCB43301.1 penicillin-binding protein 1B | 41 | 15 | 36.6 | 1 |
|  | BCB41249.1 capsular polysaccharide biosynthesis protein | 38 | 14 | 36.8 | 1.2 |
|  | BCB43011.1 ribosome-recycling factor | 44 | 16 | 36.4 | 1.9 |
|  | BCB42359.1 membrane protein | 79 | 23 | 29.1 | 3.2 |
|  | BCB41839.1 TVP38/TMEM64 family protein | 21 | 9 | 42.9 | 3.9 |
|  | BCB43386.1 UPF0149 protein | 24 | 9 | 37.5 | 5 |
|  | BCB41307.1 glutamate synthase large subunit | 20 | 8 | 40.0 | 6.4 |
| FliP1 | BCB42934.1 flagellar biosynthetic protein FliP | 289 | 289 | 100.0 | 0 |
|  | BCB45286.1 flagellar biosynthetic protein FliP | 240 | 136 | 56.7 | 5.53E-89 |
|  | BCB42177.1 EscR/YscR/HrcR family type III secretion system export apparatus protein | 209 | 82 | 39.2 | 7.74E-45 |
|  | BCB41806.1 pyruvate formate-lyase-activating enzyme | 31 | 11 | 35.5 | 3 |
|  | BCB41143.1 peptidyl-prolyl cis-trans isomerase | 61 | 23 | 37.7 | 4.1 |
|  | BCB42487.1 hypothetical protein Vag1382_16130 | 57 | 14 | 24.6 | 6 |
|  | BCB44568.1 type I secretion C-terminal target domain-containing protein | 26 | 12 | 46.2 | 8.9 |
| FliO | BCB42935.1 flagellar protein FliO | 119 | 119 | 100.0 | 1.07E-83 |
|  | BCB42994.1 UPF0294 protein | 45 | 16 | 35.6 | 1.8 |
|  | BCB43229.1 hypothetical protein Vag1382_23560 | 69 | 18 | 26.1 | 2.1 |
|  | BCB43460.1 hypothetical protein Vag1382_25870 | 59 | 17 | 28.8 | 2.2 |
|  | BCB42757.1 transporter | 25 | 11 | 44.0 | 6.5 |
|  | BCB44070.1 Bcr/CflA family drug resistance efflux transporter | 63 | 15 | 23.8 | 6.6 |
|  | BCB43234.1 WYL domain-containing protein | 26 | 10 | 38.5 | 6.9 |
| FliN1 | BCB42936.1 flagellar motor switch protein FliN | 136 | 136 | 100.0 | 3.83E-95 |
|  | BCB45285.1 flagellar motor switch protein FliN | 75 | 38 | 50.7 | 9.31E-23 |
|  | BCB42178.1 type III secretion system protein | 71 | 20 | 28.2 | 1.98E-07 |
|  | BCB42937.1 flagellar motor switch protein FliM | 34 | 11 | 32.4 | 0.31 |
|  | BCB42754.1 patatin family protein | 76 | 20 | 26.3 | 2.1 |
|  | BCB40890.1 LysR family transcriptional regulator | 47 | 12 | 25.5 | 2.4 |
|  | BCB41543.1 hypothetical protein Vag1382_06690 | 35 | 11 | 31.4 | 2.9 |
|  | BCB43212.1 plasmid transfer protein | 56 | 15 | 26.8 | 5.1 |
|  | BCB40987.1 type II secretion system protein GspE | 34 | 14 | 41.2 | 5.9 |
|  | BCB43090.1 magnesium transporter | 72 | 16 | 22.2 | 6.6 |
|  | BCB42035.1 L-serine ammonia-lyase | 27 | 11 | 40.7 | 7.3 |
|  | BCB45181.1 hypothetical protein Vag1382_43080 | 115 | 26 | 22.6 | 8.2 |
|  | BCB43884.1 chromosome partitioning protein ParB | 16 | 8 | 50.0 | 8.8 |
|  | BCB41404.1 LysR family transcriptional regulator | 20 | 8 | 40.0 | 9.2 |
|  | BCB41871.1 tol-pal system protein YbgF | 29 | 10 | 34.5 | 9.7 |
| FliM1 | BCB42937.1 flagellar motor switch protein FliM | 348 | 348 | 100.0 | 0 |
|  | BCB45284.1 flagellar motor switch protein FliM | 204 | 45 | 22.1 | 8.22E-04 |
|  | BCB42133.1 membrane protein | 94 | 28 | 29.8 | 0.011 |
|  | BCB42936.1 flagellar motor switch protein FliN | 34 | 11 | 32.4 | 0.72 |
|  | BCB41910.1 transcriptional regulator | 53 | 17 | 32.1 | 2.8 |
|  | BCB45256.1 L-threonine 3-dehydrogenase | 60 | 16 | 26.7 | 3.3 |
|  | BCB44580.1 LysR family transcriptional regulator | 16 | 6 | 37.5 | 3.5 |
|  | BCB43540.1 alanine--glyoxylate aminotransferase | 46 | 14 | 30.4 | 4.3 |
|  | BCB43394.1 DNA-binding protein | 18 | 8 | 44.4 | 5.5 |
|  | BCB42559.1 L-cystine transporter tcyP | 31 | 13 | 41.9 | 7.5 |
| FliL1 | BCB42938.1 flagellar basal body-associated protein FliL | 167 | 167 | 100.0 | 1.31E-119 |
|  | BCB43764.1 flagellar basal body-associated protein FliL | 114 | 31 | 27.2 | 1.51E-09 |
|  | BCB45300.1 flagellar protein LafL | 129 | 31 | 24.0 | 0.007 |
|  | BCB44712.1 oxidoreductase | 36 | 13 | 36.1 | 0.37 |
|  | BCB44375.1 hypothetical protein Vag1382_35020 | 28 | 14 | 50.0 | 2.2 |
|  | BCB42207.1 propionyl-CoA synthetase | 49 | 11 | 22.4 | 2.9 |
|  | BCB41181.1 malate dehydrogenase | 27 | 11 | 40.7 | 4.4 |
|  | BCB42877.1 DNA polymerase III subunit gamma/tau | 55 | 14 | 25.5 | 5.5 |
|  | BCB41109.1 cell division protein FtsN | 29 | 12 | 41.4 | 5.6 |
|  | BCB45156.1 hypothetical protein Vag1382_42830 | 50 | 19 | 38.0 | 7.5 |
|  | BCB43609.1 bifunctional aspartate kinase/homoserine dehydrogenase II | 32 | 12 | 37.5 | 7.6 |
|  | BCB45216.1 hypothetical protein Vag1382_43430 | 49 | 14 | 28.6 | 8.3 |
|  | BCB43676.1 elongation factor P--(R)-beta-lysine ligase | 60 | 15 | 25.0 | 8.4 |
| FliK1 | BCB42939.1 flagellar hook-length control protein FliK | 627 | 627 | 100.0 | 0 |
|  | BCB45299.1 flagellar hook-length control protein FliK | 109 | 29 | 26.6 | 9.06E-07 |
|  | BCB44219.1 elongation factor G | 66 | 19 | 28.8 | 1 |
|  | BCB44292.1 hypothetical protein Vag1382_34190 | 56 | 17 | 30.4 | 1.2 |
|  | BCB42926.1 response regulator | 41 | 14 | 34.1 | 3.5 |
|  | BCB43689.1 ATP-dependent 6-phosphofructokinase | 99 | 24 | 24.2 | 7.9 |
|  | BCB41962.1 tryptophan synthase alpha chain | 44 | 15 | 34.1 | 8.4 |
| FliJ1 | BCB42940.1 flagellar protein FliJ | 147 | 147 | 100.0 | 6.76E-107 |
|  | BCB44348.1 DNA-binding response regulator | 73 | 20 | 27.4 | 1.3 |
|  | BCB43499.1 nucleotide-binding protein | 45 | 17 | 37.8 | 1.8 |
|  | BCB45140.1 flavodoxin | 40 | 12 | 30.0 | 2.7 |
|  | BCB42538.1 aldehyde dehydrogenase | 40 | 10 | 25.0 | 3.5 |
|  | BCB43629.1 glutathione-regulated potassium-efflux system protein KefB | 113 | 23 | 20.4 | 4.3 |
|  | BCB42636.1 acyl-CoA dehydrogenase | 48 | 18 | 37.5 | 4.9 |
|  | BCB44166.1 flagellar hook-associated protein 1 FlgK | 39 | 14 | 35.9 | 5.9 |
| FliI1 | BCB42941.1 flagellum-specific ATPase FliI | 439 | 439 | 100.0 | 0 |
|  | BCB45277.1 flagellum-specific ATPase FliI | 437 | 234 | 53.5 | 6.72E-166 |
|  | BCB42181.1 EscN/YscN/HrcN family type III secretion system ATPase | 425 | 189 | 44.5 | 5.22E-113 |
|  | BCB43876.1 ATP synthase subunit beta | 401 | 119 | 29.7 | 4.38E-41 |
|  | BCB43878.1 ATP synthase subunit alpha | 354 | 97 | 27.4 | 3.64E-34 |
|  | BCB43808.1 transcription termination factor Rho | 234 | 62 | 26.5 | 4.15E-09 |
|  | BCB41522.1 LOG family protein | 113 | 32 | 28.3 | 0.13 |
|  | BCB43800.1 porphobilinogen deaminase | 102 | 30 | 29.4 | 0.23 |
|  | BCB44308.1 D-alanyl-D-alanine carboxypeptidase | 86 | 25 | 29.1 | 1.6 |
|  | BCB41026.1 peptide ABC transporter ATP-binding protein | 32 | 16 | 50.0 | 2.4 |
|  | BCB41025.1 peptide ABC transporter ATP-binding protein | 85 | 24 | 28.2 | 2.7 |
|  | BCB44246.1 outer membrane protein | 83 | 23 | 27.7 | 3.7 |
|  | BCB43312.1 ABC transporter ATP-binding protein | 46 | 18 | 39.1 | 3.7 |
|  | BCB45191.1 multidrug ABC transporter ATP-binding protein | 70 | 18 | 25.7 | 5.2 |
|  | BCB41616.1 porin | 44 | 15 | 34.1 | 7 |
|  | BCB43719.1 ABC transporter ATP-binding protein | 29 | 10 | 34.5 | 7.6 |
|  | BCB42231.1 amino acid ABC transporter substrate-binding protein | 34 | 11 | 32.4 | 8.2 |
|  | BCB41530.1 methionine import ATP-binding protein MetN | 51 | 17 | 33.3 | 8.3 |
|  | BCB44875.1 nitrate ABC transporter ATP-binding protein | 35 | 12 | 34.3 | 8.4 |
|  | BCB45457.1 sulfate adenylyltransferase | 23 | 11 | 47.8 | 9.3 |
| FliH1 | BCB42942.1 flagellar assembly protein FliH | 266 | 266 | 100.0 | 0 |
|  | BCB45278.1 flagellar assembly protein FliH | 195 | 57 | 29.2 | 2.54E-18 |
|  | BCB40894.1 YigZ family protein | 31 | 12 | 38.7 | 2.6 |
|  | BCB42956.1 deferrochelatase/peroxidase YfeX | 69 | 21 | 30.4 | 4.6 |
|  | BCB44574.1 TMAO reductase system periplasmic protein TorT | 40 | 15 | 37.5 | 6.5 |
|  | BCB42647.1 cytochrome c | 49 | 16 | 32.7 | 6.5 |
|  | BCB41286.1 cell division protein FtsQ | 30 | 11 | 36.7 | 8.3 |
| FliG1 | BCB42943.1 flagellar motor switch protein FliG | 351 | 351 | 100.0 | 0 |
|  | BCB45279.1 flagellar motor switch protein FliG | 321 | 98 | 30.5 | 9.08E-55 |
|  | BCB41583.1 membrane protein | 49 | 16 | 32.7 | 2.2 |
|  | BCB43648.1 HTH-type transcriptional repressor NsrR | 69 | 17 | 24.6 | 2.5 |
|  | BCB42655.1 sensor histidine kinase | 60 | 22 | 36.7 | 3.7 |
|  | BCB42217.1 agglutination protein | 19 | 10 | 52.6 | 4.4 |
|  | BCB42782.1 iron-sulfur cluster carrier protein | 30 | 9 | 30.0 | 4.7 |
|  | BCB43899.1 hypothetical protein Vag1382_30260 | 37 | 12 | 32.4 | 5.4 |
|  | BCB42332.1 N-acetyl-D-glucosamine kinase | 42 | 14 | 33.3 | 7.2 |
|  | BCB43492.1 arabinose 5-phosphate isomerase | 47 | 16 | 34.0 | 7.5 |
|  | BCB42220.1 agglutination protein | 20 | 10 | 50.0 | 8.5 |
|  | BCB43178.1 hypothetical protein Vag1382_23050 | 79 | 23 | 29.1 | 9.1 |
|  | BCB44714.1 allophanate hydrolase | 20 | 11 | 55.0 | 9.9 |
| FliF1 | BCB42944.1 flagellar M-ring protein FliF | 580 | 580 | 100.0 | 0 |
|  | BCB45280.1 flagellar M-ring protein FliF | 535 | 145 | 27.1 | 1.11E-54 |
|  | BCB42850.1 2 4-dienoyl-CoA reductase | 32 | 14 | 43.8 | 2.4 |
|  | BCB41633.1 cholera toxin homolog transcriptional activator | 116 | 30 | 25.9 | 2.5 |
|  | BCB44369.1 1 4-alpha-glucan-branching protein | 43 | 16 | 37.2 | 6.2 |
|  | BCB41355.1 riboflavin biosynthesis protein | 39 | 13 | 33.3 | 8.5 |
| FliE1 | BCB42945.1 flagellar hook-basal body complex protein FliE | 103 | 103 | 100.0 | 2.06E-72 |
|  | BCB45281.1 flagellar hook-basal body complex protein FliE | 71 | 25 | 35.2 | 5.90E-11 |
|  | BCB41006.1 hypothetical protein Vag1382_01320 | 61 | 17 | 27.9 | 0.25 |
|  | BCB43295.1 iron ABC transporter substrate-binding protein | 51 | 18 | 35.3 | 2.1 |
|  | BCB41228.1 lipoprotein | 40 | 17 | 42.5 | 4 |
|  | BCB43877.1 ATP synthase gamma chain | 28 | 11 | 39.3 | 4.4 |
|  | BCB40945.1 hypothetical protein Vag1382_00710 | 66 | 19 | 28.8 | 6.3 |
|  | BCB42190.1 hypothetical protein Vag1382_13160 | 25 | 8 | 32.0 | 7.4 |
|  | BCB44210.1 membrane protein | 19 | 9 | 47.4 | 9.2 |
|  | BCB42111.1 hypothetical protein Vag1382_12370 | 17 | 9 | 52.9 | 9.3 |
|  | BCB45441.1 AsnC family transcriptional regulator | 20 | 7 | 35.0 | 9.9 |
| FlaM | BCB42946.1 sigma-54-dependent Fis family transcriptional regulator FlaM | 469 | 469 | 100.0 | 0 |
|  | BCB45282.1 sigma-54-dependent Fis family transcriptional regulator LafK | 467 | 199 | 42.6 | 3.64E-117 |
|  | BCB41724.1 sigma-54-dependent Fis family transcriptional regulator | 477 | 170 | 35.6 | 2.96E-92 |
|  | BCB40971.1 nitrogen regulation protein NR(I) | 379 | 140 | 36.9 | 3.59E-88 |
|  | BCB42325.1 sigma-54-dependent Fis family transcriptional regulator | 466 | 165 | 35.4 | 1.51E-83 |
|  | BCB42948.1 sigma-54-dependent Fis family transcriptional regulator FlaK | 229 | 122 | 53.3 | 2.08E-78 |
|  | BCB44437.1 sigma-54-dependent Fis family transcriptional regulator | 335 | 140 | 41.8 | 3.22E-78 |
|  | BCB42814.1 regulatory protein LuxO | 467 | 152 | 32.5 | 5.58E-78 |
|  | BCB44084.1 two-component system response regulator | 472 | 155 | 32.8 | 2.06E-77 |
|  | BCB40938.1 sigma-54-dependent Fis family transcriptional regulator | 469 | 163 | 34.8 | 1.94E-75 |
|  | BCB43172.1 acetoacetate metabolism regulatory protein AtoC | 465 | 160 | 34.4 | 3.61E-74 |
|  | BCB42354.1 sigma-54-dependent Fis family transcriptional regulator | 490 | 150 | 30.6 | 2.93E-73 |
|  | BCB41925.1 sigma-54-dependent Fis family transcriptional regulator | 276 | 111 | 40.2 | 1.29E-61 |
|  | BCB42045.1 TyrR family transcriptional regulator | 330 | 116 | 35.2 | 1.04E-60 |
|  | BCB42666.1 anaerobic nitric oxide reductase transcription regulator | 211 | 98 | 46.4 | 2.90E-60 |
|  | BCB42684.1 phage shock protein operon transcriptional activator | 223 | 91 | 40.8 | 2.50E-54 |
|  | BCB42144.1 sigma-54-dependent Fis family transcriptional regulator | 220 | 85 | 38.6 | 3.96E-48 |
|  | BCB41338.1 sigma-54-dependent Fis family transcriptional regulator | 239 | 87 | 36.4 | 2.00E-43 |
|  | BCB41221.1 sigma-54-dependent Fis family transcriptional regulator | 330 | 90 | 27.3 | 8.57E-34 |
|  | BCB44663.1 two-component system response regulator | 316 | 81 | 25.6 | 6.31E-28 |
|  | BCB45455.1 DNA-binding response regulator | 472 | 101 | 21.4 | 5.70E-24 |
|  | BCB41392.1 DNA-binding response regulator | 161 | 51 | 31.7 | 6.34E-17 |
|  | BCB42438.1 DNA-binding response regulator | 110 | 41 | 37.3 | 6.38E-16 |
|  | BCB45406.1 DNA-binding response regulator | 115 | 39 | 33.9 | 3.53E-14 |
|  | BCB44892.1 DNA-binding response regulator | 111 | 38 | 34.2 | 7.42E-14 |
|  | BCB41314.1 DNA-binding response regulator | 121 | 38 | 31.4 | 3.37E-13 |
|  | BCB43692.1 DNA-binding response regulator | 109 | 36 | 33.0 | 2.23E-12 |
|  | BCB41942.1 DNA-binding response regulator | 108 | 36 | 33.3 | 2.43E-12 |
|  | BCB42926.1 response regulator | 110 | 42 | 38.2 | 2.87E-12 |
|  | BCB44754.1 DNA-binding response regulator | 180 | 53 | 29.4 | 6.05E-12 |
|  | BCB42654.1 DNA-binding response regulator | 102 | 35 | 34.3 | 1.08E-11 |
|  | BCB44049.1 DNA-binding response regulator | 103 | 35 | 34.0 | 7.33E-11 |
|  | BCB41007.1 DNA-binding response regulator | 108 | 33 | 30.6 | 3.14E-10 |
|  | BCB41978.1 DNA-binding response regulator | 116 | 36 | 31.0 | 5.98E-10 |
|  | BCB41215.1 DNA-binding response regulator | 135 | 38 | 28.1 | 6.18E-10 |
|  | BCB44327.1 DNA-binding response regulator | 107 | 34 | 31.8 | 4.52E-09 |
|  | BCB41843.1 two-component system response regulator TorR | 122 | 35 | 28.7 | 6.72E-09 |
|  | BCB45189.1 DNA-binding response regulator | 130 | 31 | 23.8 | 1.54E-08 |
|  | BCB42881.1 two-component system response regulator | 103 | 25 | 24.3 | 4.15E-08 |
|  | BCB43282.1 histidine kinase | 109 | 30 | 27.5 | 5.68E-08 |
|  | BCB45263.1 DNA-binding response regulator | 104 | 30 | 28.8 | 7.12E-08 |
|  | BCB44611.1 chemotaxis protein CheV | 131 | 33 | 25.2 | 1.99E-06 |
|  | BCB42136.1 transcriptional regulatory protein | 104 | 32 | 30.8 | 2.07E-06 |
|  | BCB44348.1 DNA-binding response regulator | 112 | 30 | 26.8 | 5.02E-06 |
|  | BCB44508.1 histidine kinase | 119 | 30 | 25.2 | 8.07E-06 |
|  | BCB41363.1 putative response regulatory protein | 115 | 38 | 33.0 | 1.02E-05 |
|  | BCB43942.1 putative response regulatory protein | 118 | 36 | 30.5 | 2.11E-05 |
|  | BCB44604.1 hybrid sensor histidine kinase/response regulator | 103 | 25 | 24.3 | 4.67E-05 |
|  | BCB44575.1 histidine kinase | 173 | 42 | 24.3 | 7.50E-05 |
|  | BCB41957.1 hybrid sensor histidine kinase/response regulator | 115 | 31 | 27.0 | 9.58E-05 |
|  | BCB42017.1 DNA-binding response regulator | 67 | 19 | 28.4 | 1.17E-04 |
|  | BCB43932.1 hybrid sensor histidine kinase/response regulator | 121 | 33 | 27.3 | 1.39E-04 |
|  | BCB44762.1 diguanylate cyclase response regulator | 108 | 27 | 25.0 | 2.81E-04 |
|  | BCB42728.1 DNA-binding response regulator | 103 | 24 | 23.3 | 2.95E-04 |
|  | BCB44607.1 diguanylate phosphodiesterase | 181 | 40 | 22.1 | 0.001 |
|  | BCB43366.1 histidine kinase | 74 | 22 | 29.7 | 0.001 |
|  | BCB41312.1 aerobic respiration control sensor protein | 116 | 25 | 21.6 | 0.002 |
|  | BCB44366.1 autoinducer 2 sensor kinase/phosphatase LuxQ | 111 | 33 | 29.7 | 0.003 |
|  | BCB42502.1 two-component system sensor histidine kinase/response regulator | 118 | 31 | 26.3 | 0.003 |
|  | BCB42502.1 two-component system sensor histidine kinase/response regulator | 139 | 32 | 23.0 | 0.008 |
|  | BCB42923.1 chemotaxis response regulator protein-glutamate methylesterase of group 1 operon CheB | 106 | 27 | 25.5 | 0.005 |
|  | BCB42285.1 hybrid sensor histidine kinase/response regulator | 102 | 26 | 25.5 | 0.009 |
|  | BCB42342.1 transcriptional regulator | 174 | 36 | 20.7 | 0.011 |
|  | BCB43700.1 DNA-binding response regulator | 108 | 30 | 27.8 | 0.011 |
|  | BCB42623.1 response regulator | 83 | 26 | 31.3 | 0.015 |
|  | BCB41591.1 chemotaxis protein CheW | 121 | 29 | 24.0 | 0.016 |
|  | BCB42751.1 chemotaxis protein CheW | 124 | 31 | 25.0 | 0.021 |
|  | BCB44392.1 DNA-binding response regulator | 138 | 35 | 25.4 | 0.026 |
|  | BCB44016.1 CdaR family transcriptional regulator | 34 | 15 | 44.1 | 0.044 |
|  | BCB42039.1 hybrid sensor histidine kinase/response regulator | 72 | 16 | 22.2 | 0.08 |
|  | BCB41728.1 ATP-dependent Clp protease ATP-binding subunit ClpX | 140 | 34 | 24.3 | 0.091 |
|  | BCB44537.1 hybrid sensor histidine kinase/response regulator | 109 | 28 | 25.7 | 0.11 |
|  | BCB44626.1 transcriptional regulatory protein | 121 | 30 | 24.8 | 0.25 |
|  | BCB45119.1 chemotaxis protein CheW | 65 | 21 | 32.3 | 0.34 |
|  | BCB40929.1 DNA-binding transcriptional regulator AsnC | 47 | 14 | 29.8 | 0.49 |
|  | BCB41107.1 ATP-dependent protease ATPase subunit HslU | 43 | 11 | 25.6 | 0.75 |
|  | BCB42369.1 ATP-dependent RNA helicase HrpA | 99 | 24 | 24.2 | 0.93 |
|  | BCB43309.1 pantothenate synthetase | 45 | 15 | 33.3 | 1.3 |
|  | BCB42228.1 arginine ABC transporter ATP-binding protein | 27 | 11 | 40.7 | 1.3 |
|  | BCB44424.1 transcriptional regulator | 107 | 26 | 24.3 | 1.4 |
|  | BCB42168.1 hypothetical protein Vag1382_12940 | 52 | 14 | 26.9 | 2.1 |
|  | BCB44959.1 ATP-dependent RNA helicase DeaD | 50 | 17 | 34.0 | 2.4 |
|  | BCB45343.1 hypothetical protein Vag1382_44700 | 79 | 20 | 25.3 | 2.5 |
|  | BCB44071.1 agmatinase | 80 | 21 | 26.3 | 3.1 |
|  | BCB40964.1 cytochrome c | 66 | 19 | 28.8 | 3.5 |
|  | BCB44630.1 hypothetical protein Vag1382_37570 | 54 | 20 | 37.0 | 3.7 |
|  | BCB43864.1 ATP-dependent protease | 146 | 33 | 22.6 | 4.4 |
|  | BCB41541.1 UPF0250 protein | 18 | 9 | 50.0 | 4.6 |
|  | BCB41692.1 SAM-dependent methyltransferase | 60 | 14 | 23.3 | 5.1 |
|  | BCB44562.1 iron(III) ABC transporter ATP-binding protein | 37 | 12 | 32.4 | 6.4 |
|  | BCB43534.1 maturase | 47 | 19 | 40.4 | 6.9 |
|  | BCB43268.1 ribosomal RNA large subunit methyltransferase E | 36 | 12 | 33.3 | 8.5 |
|  | BCB43078.1 RecBCD enzyme subunit RecC | 64 | 16 | 25.0 | 9.2 |
| FlaL | BCB42947.1 sensor histidine kinase FlaL | 343 | 343 | 100.0 | 0 |
|  | BCB41725.1 ATPase | 238 | 66 | 27.7 | 3.73E-21 |
|  | BCB43693.1 two-component sensor histidine kinase | 240 | 67 | 27.9 | 6.20E-16 |
|  | BCB43932.1 hybrid sensor histidine kinase/response regulator | 225 | 63 | 28.0 | 7.00E-16 |
|  | BCB42324.1 two-component sensor histidine kinase | 232 | 65 | 28.0 | 7.76E-16 |
|  | BCB44575.1 histidine kinase | 227 | 60 | 26.4 | 1.84E-15 |
|  | BCB45405.1 two-component sensor histidine kinase | 215 | 60 | 27.9 | 5.99E-15 |
|  | BCB43366.1 histidine kinase | 248 | 64 | 25.8 | 8.38E-15 |
|  | BCB40972.1 two-component system sensor histidine kinase NtrB | 349 | 81 | 23.2 | 9.19E-15 |
|  | BCB44537.1 hybrid sensor histidine kinase/response regulator | 235 | 63 | 26.8 | 1.09E-14 |
|  | BCB42439.1 two-component sensor histidine kinase | 221 | 54 | 24.4 | 1.18E-14 |
|  | BCB41393.1 PAS domain-containing sensor histidine kinase | 351 | 81 | 23.1 | 1.66E-14 |
|  | BCB44508.1 histidine kinase | 255 | 63 | 24.7 | 1.68E-14 |
|  | BCB41216.1 two-component sensor histidine kinase | 230 | 62 | 27.0 | 2.38E-14 |
|  | BCB41878.1 sensor histidine kinase | 258 | 65 | 25.2 | 4.14E-14 |
|  | BCB45454.1 signal transduction histidine kinase | 242 | 57 | 23.6 | 1.15E-13 |
|  | BCB42039.1 hybrid sensor histidine kinase/response regulator | 227 | 64 | 28.2 | 2.74E-13 |
|  | BCB41008.1 two-component sensor histidine kinase | 221 | 56 | 25.3 | 1.96E-12 |
|  | BCB42502.1 two-component system sensor histidine kinase/response regulator | 218 | 57 | 26.1 | 2.07E-12 |
|  | BCB44326.1 sensor histidine kinase | 232 | 55 | 23.7 | 5.51E-12 |
|  | BCB44366.1 autoinducer 2 sensor kinase/phosphatase LuxQ | 243 | 64 | 26.3 | 7.09E-12 |
|  | BCB41312.1 aerobic respiration control sensor protein | 229 | 56 | 24.5 | 1.84E-11 |
|  | BCB44662.1 ATPase | 379 | 88 | 23.2 | 3.94E-11 |
|  | BCB43707.1 sensor histidine kinase | 235 | 58 | 24.7 | 5.96E-11 |
|  | BCB42285.1 hybrid sensor histidine kinase/response regulator | 270 | 63 | 23.3 | 6.04E-11 |
|  | BCB43282.1 histidine kinase | 244 | 62 | 25.4 | 2.25E-10 |
|  | BCB44359.1 hybrid sensor histidine kinase/response regulator | 249 | 58 | 23.3 | 2.85E-10 |
|  | BCB44349.1 hypothetical protein Vag1382_34760 | 222 | 56 | 25.2 | 1.38E-09 |
|  | BCB44050.1 two-component sensor histidine kinase | 220 | 56 | 25.5 | 3.40E-09 |
|  | BCB44083.1 signal transduction histidine kinase | 235 | 51 | 21.7 | 1.01E-08 |
|  | BCB44083.1 signal transduction histidine kinase | 54 | 16 | 29.6 | 0.24 |
|  | BCB42622.1 transcriptional regulator | 108 | 33 | 30.6 | 7.36E-08 |
|  | BCB42622.1 transcriptional regulator | 31 | 9 | 29.0 | 4.4 |
|  | BCB45190.1 sensor histidine kinase | 203 | 49 | 24.1 | 9.58E-08 |
|  | BCB44604.1 hybrid sensor histidine kinase/response regulator | 111 | 29 | 26.1 | 1.00E-07 |
|  | BCB42729.1 sensor histidine kinase | 237 | 52 | 21.9 | 2.48E-06 |
|  | BCB41957.1 hybrid sensor histidine kinase/response regulator | 97 | 36 | 37.1 | 1.63E-05 |
|  | BCB44755.1 sensor histidine kinase | 222 | 54 | 24.3 | 2.50E-05 |
|  | BCB42655.1 sensor histidine kinase | 126 | 32 | 25.4 | 6.22E-05 |
|  | BCB42407.1 sensor histidine kinase | 124 | 30 | 24.2 | 6.84E-04 |
|  | BCB44625.1 signal transduction histidine kinase | 101 | 31 | 30.7 | 0.002 |
|  | BCB42924.1 chemotaxis protein CheA | 182 | 40 | 22.0 | 0.015 |
|  | BCB41941.1 two-component sensor histidine kinase | 215 | 50 | 23.3 | 0.021 |
|  | BCB42135.1 histidine kinase | 340 | 77 | 22.6 | 0.036 |
|  | BCB45262.1 histidine kinase | 198 | 43 | 21.7 | 0.55 |
|  | BCB42369.1 ATP-dependent RNA helicase HrpA | 41 | 12 | 29.3 | 1.2 |
|  | BCB42405.1 sensor domain-containing phosphodiesterase | 97 | 22 | 22.7 | 5.9 |
|  | BCB45420.1 osmotically inducible protein C | 41 | 13 | 31.7 | 6 |
|  | BCB42242.1 GAF domain protein | 62 | 14 | 22.6 | 8.6 |
|  | BCB43302.1 ATP-dependent helicase HrpB | 40 | 14 | 35.0 | 9.1 |
| FlaK | BCB42948.1 sigma-54-dependent Fis family transcriptional regulator FlaK | 488 | 488 | 100.0 | 0 |
|  | BCB40971.1 nitrogen regulation protein NR(I) | 352 | 150 | 42.6 | 2.81E-81 |
|  | BCB41724.1 sigma-54-dependent Fis family transcriptional regulator | 237 | 118 | 49.8 | 3.03E-79 |
|  | BCB42946.1 sigma-54-dependent Fis family transcriptional regulator FlaM | 229 | 122 | 53.3 | 2.16E-78 |
|  | BCB42325.1 sigma-54-dependent Fis family transcriptional regulator | 320 | 141 | 44.1 | 8.83E-77 |
|  | BCB42666.1 anaerobic nitric oxide reductase transcription regulator | 353 | 134 | 38.0 | 1.13E-74 |
|  | BCB41925.1 sigma-54-dependent Fis family transcriptional regulator | 368 | 138 | 37.5 | 6.30E-74 |
|  | BCB45282.1 sigma-54-dependent Fis family transcriptional regulator LafK | 284 | 122 | 43.0 | 1.29E-73 |
|  | BCB40938.1 sigma-54-dependent Fis family transcriptional regulator | 471 | 162 | 34.4 | 3.28E-72 |
|  | BCB42354.1 sigma-54-dependent Fis family transcriptional regulator | 286 | 122 | 42.7 | 1.02E-71 |
|  | BCB44437.1 sigma-54-dependent Fis family transcriptional regulator | 366 | 138 | 37.7 | 3.30E-71 |
|  | BCB42814.1 regulatory protein LuxO | 398 | 142 | 35.7 | 1.52E-70 |
|  | BCB44084.1 two-component system response regulator | 419 | 154 | 36.8 | 1.16E-67 |
|  | BCB43172.1 acetoacetate metabolism regulatory protein AtoC | 334 | 136 | 40.7 | 1.77E-67 |
|  | BCB42684.1 phage shock protein operon transcriptional activator | 354 | 122 | 34.5 | 9.39E-60 |
|  | BCB42045.1 TyrR family transcriptional regulator | 246 | 95 | 38.6 | 2.09E-52 |
|  | BCB42144.1 sigma-54-dependent Fis family transcriptional regulator | 242 | 96 | 39.7 | 2.15E-52 |
|  | BCB41338.1 sigma-54-dependent Fis family transcriptional regulator | 474 | 131 | 27.6 | 5.92E-45 |
|  | BCB41221.1 sigma-54-dependent Fis family transcriptional regulator | 271 | 80 | 29.5 | 8.32E-37 |
|  | BCB44663.1 two-component system response regulator | 426 | 105 | 24.6 | 5.85E-16 |
|  | BCB43717.1 DNA-binding protein Fis | 52 | 19 | 36.5 | 0.009 |
|  | BCB45455.1 DNA-binding response regulator | 60 | 17 | 28.3 | 0.096 |
|  | BCB43864.1 ATP-dependent protease | 145 | 31 | 21.4 | 0.14 |
|  | BCB42728.1 DNA-binding response regulator | 152 | 35 | 23.0 | 0.18 |
|  | BCB45394.1 Fe3+ dicitrate ABC transporter permease | 32 | 12 | 37.5 | 1.4 |
|  | BCB43207.1 hypothetical protein Vag1382_23340 | 34 | 15 | 44.1 | 2.6 |
|  | BCB41242.1 general secretion pathway protein GspA | 93 | 24 | 25.8 | 3.5 |
|  | BCB44065.1 ABC transporter ATP-binding protein | 125 | 33 | 26.4 | 5.3 |
|  | BCB44285.1 lipoate--protein ligase A | 61 | 16 | 26.2 | 7.9 |
|  | BCB43922.1 arginine ABC transporter ATP-binding protein | 27 | 9 | 33.3 | 8.1 |
|  | BCB44834.1 ATPase AAA | 165 | 39 | 23.6 | 8.5 |
| FlaJ1 | BCB42949.1 flagellar protein FliS | 136 | 136 | 100.0 | 1.25E-98 |
|  | BCB45297.1 flagellar protein FliS | 119 | 36 | 30.3 | 3.89E-19 |
|  | BCB41876.1 hypothetical protein Vag1382_10020 | 18 | 8 | 44.4 | 2 |
|  | BCB42405.1 sensor domain-containing phosphodiesterase | 24 | 11 | 45.8 | 2.7 |
|  | BCB42658.1 bifunctional metallophosphatase/5'-nucleotidase | 33 | 11 | 33.3 | 9.2 |
|  | BCB41932.1 imidazole glycerol phosphate synthase subunit HisF | 34 | 11 | 32.4 | 9.9 |
| FlaI1 | BCB42950.1 flagellar protein FliT | 101 | 101 | 100.0 | 6.90E-70 |
|  | BCB43292.1 phosphoglucomutase/phosphomannomutase | 44 | 14 | 31.8 | 1.5 |
|  | BCB41523.1 GGDEF domain-containing protein | 32 | 14 | 43.8 | 5.5 |
|  | BCB45411.1 hypothetical protein Vag1382_45380 | 58 | 15 | 25.9 | 7 |
|  | BCB44694.1 lipase | 23 | 8 | 34.8 | 7.7 |
|  | BCB45156.1 hypothetical protein Vag1382_42830 | 22 | 9 | 40.9 | 9 |
|  | BCB43177.1 hypothetical protein Vag1382_23040 | 26 | 10 | 38.5 | 9.7 |
| FlaH1 | BCB42951.1 polar flagellar hook-associated protein 2 | 663 | 663 | 100.0 | 0 |
|  | BCB45296.1 lateral flagellar hook-associated protein 2 | 256 | 63 | 24.6 | 5.10E-17 |
|  | BCB45296.1 lateral flagellar hook-associated protein 2 | 204 | 51 | 25.0 | 6.70E-12 |
|  | BCB42670.1 RNA pseudouridine synthase | 55 | 22 | 40.0 | 0.039 |
|  | BCB42775.1 ribosomal large subunit pseudouridine synthase C | 40 | 14 | 35.0 | 1.2 |
|  | BCB43331.1 tRNA (guanine-N(1)-)-methyltransferase | 67 | 20 | 29.9 | 1.5 |
|  | BCB41899.1 serine--tRNA ligase | 30 | 15 | 50.0 | 2.3 |
|  | BCB42180.1 type III secretion protein | 43 | 18 | 41.9 | 3.2 |
|  | BCB41147.1 Crp/Fnr family transcriptional regulator | 62 | 16 | 25.8 | 4.5 |
|  | BCB41807.1 exonuclease | 93 | 24 | 25.8 | 6.5 |
|  | BCB43932.1 hybrid sensor histidine kinase/response regulator | 40 | 13 | 32.5 | 7.6 |
|  | BCB43109.1 hypothetical protein Vag1382_22360 | 40 | 12 | 30.0 | 9.3 |
| FlaG | BCB42952.1 protein FlaG | 144 | 144 | 100.0 | 3.37E-106 |
|  | BCB40968.1 coproporphyrinogen-III oxidase | 48 | 16 | 33.3 | 0.21 |
|  | BCB42685.1 ABC transporter substrate-binding protein | 52 | 15 | 28.8 | 0.34 |
|  | BCB44537.1 hybrid sensor histidine kinase/response regulator | 59 | 18 | 30.5 | 0.57 |
|  | BCB44272.1 GntR family transcriptional regulator | 29 | 11 | 37.9 | 1.2 |
|  | BCB45019.1 cold-shock protein | 30 | 12 | 40.0 | 1.4 |
|  | BCB42283.1 hypothetical protein Vag1382_14090 | 47 | 16 | 34.0 | 1.7 |
|  | BCB42877.1 DNA polymerase III subunit gamma/tau | 86 | 21 | 24.4 | 3.4 |
|  | BCB41371.1 phospho-2-dehydro-3-deoxyheptonate aldolase | 48 | 14 | 29.2 | 4.4 |
|  | BCB41328.1 UPF0246 protein | 20 | 9 | 45.0 | 5.6 |
|  | BCB44498.1 hypothetical protein Vag1382_36250 | 54 | 18 | 33.3 | 5.8 |
|  | BCB41494.1 aminoacyl-histidine dipeptidase | 15 | 8 | 53.3 | 8.8 |
|  | BCB42087.1 CopG family transcriptional regulator | 57 | 18 | 31.6 | 9 |
|  | BCB42648.1 outer membrane protein | 42 | 16 | 38.1 | 9.7 |
|  | BCB41701.1 histidine ammonia-lyase | 40 | 17 | 42.5 | 9.9 |
| FlaA | BCB42953.1 polar flagellin A | 376 | 376 | 100.0 | 0 |
|  | BCB42954.1 flagellin B | 377 | 291 | 77.2 | 0 |
|  | BCB41606.1 flagellin D | 377 | 290 | 76.9 | 0 |
|  | BCB42955.1 polar flagellin F | 377 | 252 | 66.8 | 1.35E-176 |
|  | BCB41605.1 polar flagellin C | 384 | 249 | 64.8 | 3.65E-173 |
|  | BCB41607.1 polar flagellin E | 374 | 186 | 49.7 | 1.01E-125 |
|  | BCB45292.1 lateral flagellin LafA | 161 | 84 | 52.2 | 3.31E-45 |
|  | BCB45292.1 lateral flagellin LafA | 81 | 36 | 44.4 | 1.42E-16 |
|  | BCB44956.1 chemotaxis protein | 68 | 22 | 32.4 | 0.53 |
|  | BCB44167.1 flagellar hook-associated protein 3 FlgL | 179 | 49 | 27.4 | 0.77 |
|  | BCB43088.1 DeoR/GlpR family transcriptional regulator | 30 | 12 | 40.0 | 2.5 |
|  | BCB42221.1 calcium-binding protein | 111 | 32 | 28.8 | 3.9 |
|  | BCB42221.1 calcium-binding protein | 110 | 31 | 28.2 | 9.6 |
|  | BCB42221.1 calcium-binding protein | 110 | 31 | 28.2 | 9.6 |
|  | BCB44483.1 ABC transporter ATP-binding protein | 36 | 18 | 50.0 | 8.8 |
| FlaB | BCB42954.1 flagellin B | 377 | 377 | 100.0 | 0 |
|  | BCB41606.1 flagellin D | 377 | 376 | 99.7 | 0 |
|  | BCB42953.1 polar flagellin A | 377 | 291 | 77.2 | 0 |
|  | BCB42955.1 polar flagellin F | 377 | 263 | 69.8 | 0 |
|  | BCB41605.1 polar flagellin C | 383 | 255 | 66.6 | 5.49E-180 |
|  | BCB41607.1 polar flagellin E | 375 | 200 | 53.3 | 1.14E-134 |
|  | BCB45292.1 lateral flagellin LafA | 171 | 90 | 52.6 | 4.19E-48 |
|  | BCB45292.1 lateral flagellin LafA | 79 | 36 | 45.6 | 7.05E-17 |
|  | BCB45296.1 lateral flagellar hook-associated protein 2 | 125 | 34 | 27.2 | 0.26 |
|  | BCB44167.1 flagellar hook-associated protein 3 FlgL | 53 | 16 | 30.2 | 0.97 |
|  | BCB43233.1 type I restriction-modification system subunit M | 44 | 16 | 36.4 | 2.6 |
|  | BCB44176.1 60 kDa chaperonin 2 | 28 | 15 | 53.6 | 2.9 |
|  | BCB40997.1 3'(2') 5'-bisphosphate nucleotidase CysQ | 49 | 16 | 32.7 | 2.9 |
|  | BCB43229.1 hypothetical protein Vag1382_23560 | 49 | 14 | 28.6 | 4.4 |
|  | BCB44040.1 putative pseudouridine methyltransferase | 32 | 11 | 34.4 | 6.4 |
|  | BCB41218.1 dihydroxyacetone kinase subunit DhaK | 25 | 12 | 48.0 | 8.9 |
| FlaF | BCB42955.1 polar flagellin F | 377 | 377 | 100.0 | 0 |
|  | BCB42954.1 flagellin B | 377 | 263 | 69.8 | 0 |
|  | BCB41606.1 flagellin D | 377 | 263 | 69.8 | 0 |
|  | BCB42953.1 polar flagellin A | 377 | 252 | 66.8 | 1.35E-176 |
|  | BCB41605.1 polar flagellin C | 384 | 247 | 64.3 | 1.49E-170 |
|  | BCB41607.1 polar flagellin E | 374 | 177 | 47.3 | 7.89E-119 |
|  | BCB45292.1 lateral flagellin LafA | 171 | 96 | 56.1 | 1.39E-53 |
|  | BCB45292.1 lateral flagellin LafA | 72 | 31 | 43.1 | 9.49E-15 |
|  | BCB44167.1 flagellar hook-associated protein 3 FlgL | 134 | 31 | 23.1 | 0.023 |
|  | BCB45357.1 membrane protein | 31 | 14 | 45.2 | 1.8 |
|  | BCB43647.1 ribonuclease R | 41 | 16 | 39.0 | 1.9 |
|  | BCB45407.1 PTS glucose transporter subunit IIBC | 37 | 14 | 37.8 | 2.7 |
|  | BCB41055.1 UDP-N-acetyl glucosamine 2-epimerase | 95 | 26 | 27.4 | 2.7 |
|  | BCB45127.1 hemin ABC transporter substrate-binding protein | 51 | 17 | 33.3 | 3.5 |
|  | BCB43076.1 RecBCD enzyme subunit RecD | 91 | 26 | 28.6 | 6.1 |
|  | BCB41882.1 multidrug efflux RND transporter permease subunit | 64 | 17 | 26.6 | 6.3 |
|  | BCB44864.1 serine/threonine protein kinase | 46 | 15 | 32.6 | 7.6 |
|  | BCB41421.1 chaperone protein HscA | 32 | 10 | 31.3 | 8.1 |
|  | BCB42464.1 hypothetical protein Vag1382_15900 | 141 | 36 | 25.5 | 9.2 |
| RpoN | BCB43496.1 RNA polymerase sigma-54 factor | 489 | 489 | 100.0 | 0 |
|  | BCB43637.1 DUF1338 domain-containing protein | 49 | 16 | 32.7 | 0.9 |
|  | BCB44591.1 putative lipid kinase YegS-like protein | 74 | 22 | 29.7 | 4.8 |
|  | BCB41460.1 hydrolase | 55 | 17 | 30.9 | 6.3 |
|  | BCB43007.1 zinc metalloprotease | 77 | 22 | 28.6 | 6.6 |
|  | BCB43780.1 MFS transporter | 109 | 24 | 22.0 | 6.7 |
|  | BCB43657.1 tRNA dimethylallyltransferase | 32 | 10 | 31.3 | 7.4 |
|  | BCB41887.1 phospholipase C | 20 | 10 | 50.0 | 7.9 |
|  | BCB44935.1 enoyl-CoA hydratase | 40 | 14 | 35.0 | 8.2 |
| ZomB | BCB41891.1 hypothetical protein Vag1382_10170 | 254 | 254 | 100.0 | 0 |
|  | BCB41307.1 glutamate synthase large subunit | 66 | 21 | 31.8 | 0.25 |
|  | BCB43069.1 tRNA threonylcarbamoyladenosine dehydratase | 87 | 23 | 26.4 | 3 |
|  | BCB44398.1 transporter | 90 | 24 | 26.7 | 4 |
|  | BCB42214.1 GGDEF domain-containing protein | 33 | 14 | 42.4 | 4.5 |
|  | BCB41285.1 UDP-N-acetylmuramate--L-alanine ligase | 66 | 16 | 24.2 | 4.9 |
|  | BCB42619.1 type-1 fimbrial protein subunit A | 31 | 12 | 38.7 | 7.8 |
| PomA | BCB41513.1 flagellar motor protein PomA | 253 | 253 | 100.0 | 0 |
|  | BCB45125.1 biopolymer transporter ExbB | 109 | 26 | 23.9 | 7.75E-04 |
|  | BCB41019.1 biopolymer transporter ExbB | 60 | 17 | 28.3 | 0.007 |
|  | BCB41018.1 biopolymer transporter ExbB | 99 | 22 | 22.2 | 0.054 |
|  | BCB44053.1 flagellar motor protein MotA | 69 | 19 | 27.5 | 0.095 |
|  | BCB41866.1 Tol-Pal system subunit TolQ | 67 | 18 | 26.9 | 0.2 |
|  | BCB44054.1 hypothetical protein Vag1382_31810 | 78 | 19 | 24.4 | 0.3 |
|  | BCB44756.1 NAD(P) transhydrogenase subunit beta | 18 | 10 | 55.6 | 1 |
|  | BCB41421.1 chaperone protein HscA | 26 | 10 | 38.5 | 1.8 |
|  | BCB44958.1 GGDEF-domain containing protein | 63 | 21 | 33.3 | 2.4 |
|  | BCB42928.1 site-determining protein FlhG | 86 | 23 | 26.7 | 6.8 |
|  | BCB42418.1 anaerobic sulfatase maturase | 56 | 14 | 25.0 | 9 |
| PomB | BCB41514.1 flagellar motor protein PomB | 315 | 315 | 100.0 | 0 |
|  | BCB44860.1 flagellar motor protein | 157 | 52 | 33.1 | 1.88E-23 |
|  | BCB42593.1 lipoprotein | 136 | 41 | 30.1 | 1.98E-09 |
|  | BCB45303.1 chemotaxis protein LafU | 152 | 45 | 29.6 | 1.04E-08 |
|  | BCB45023.1 membrane protein | 201 | 51 | 25.4 | 6.41E-08 |
|  | BCB44142.1 membrane protein | 76 | 25 | 32.9 | 9.29E-07 |
|  | BCB41583.1 membrane protein | 108 | 33 | 30.6 | 1.25E-06 |
|  | BCB42614.1 porin OmpA | 114 | 36 | 31.6 | 7.78E-06 |
|  | BCB42219.1 outer membrane protein | 107 | 30 | 28.0 | 1.89E-05 |
|  | BCB44402.1 membrane protein | 108 | 33 | 30.6 | 2.50E-05 |
|  | BCB42480.1 chemotaxis protein MotB | 202 | 46 | 22.8 | 3.28E-05 |
|  | BCB42216.1 outer membrane protein | 129 | 35 | 27.1 | 6.35E-04 |
|  | BCB44907.1 outer membrane protein | 143 | 35 | 24.5 | 0.004 |
|  | BCB45283.1 sodium-type flagellar protein MotY | 78 | 24 | 30.8 | 0.014 |
|  | BCB41870.1 peptidoglycan-associated lipoprotein | 101 | 24 | 23.8 | 0.2 |
|  | BCB42825.1 sodium-type flagellar protein MotY | 90 | 27 | 30.0 | 0.25 |
|  | BCB45471.1 polymerase | 74 | 26 | 35.1 | 2.2 |
|  | BCB44193.1 alpha-1 2-mannosidase | 41 | 13 | 31.7 | 2.6 |
|  | BCB41039.1 formamidopyrimidine-DNA glycosylase | 35 | 14 | 40.0 | 3.1 |
|  | BCB41912.1 amine oxidase | 34 | 12 | 35.3 | 6.7 |
|  | BCB41242.1 general secretion pathway protein GspA | 60 | 23 | 38.3 | 6.9 |
|  | BCB44778.1 acriflavine resistance protein B | 64 | 16 | 25.0 | 8.5 |
|  | BCB42806.1 peptide ABC transporter substrate-binding protein | 57 | 15 | 26.3 | 9.5 |
| MotX | BCB43650.1 sodium-type polar flagellar protein MotX | 211 | 211 | 100.0 | 8.57E-158 |
|  | BCB40892.1 3-ketoacyl-CoA thiolase | 79 | 26 | 32.9 | 0.11 |
|  | BCB42297.1 universal stress protein E | 77 | 23 | 29.9 | 0.24 |
|  | BCB45376.1 hypothetical protein Vag1382_45030 | 77 | 21 | 27.3 | 1.7 |
|  | BCB43015.1 methionine aminopeptidase | 64 | 17 | 26.6 | 1.9 |
|  | BCB42712.1 5-methyltetrahydropteroyltriglutamate--homocysteine methyltransferase | 38 | 13 | 34.2 | 5.1 |
| MotY | BCB42825.1 sodium-type flagellar protein MotY | 293 | 293 | 100.0 | 0 |
|  | BCB45283.1 sodium-type flagellar protein MotY | 247 | 63 | 25.5 | 8.12E-24 |
|  | BCB44402.1 membrane protein | 100 | 36 | 36.0 | 8.56E-15 |
|  | BCB42593.1 lipoprotein | 96 | 38 | 39.6 | 3.21E-12 |
|  | BCB45023.1 membrane protein | 153 | 40 | 26.1 | 7.36E-11 |
|  | BCB44142.1 membrane protein | 69 | 25 | 36.2 | 5.05E-10 |
|  | BCB41583.1 membrane protein | 111 | 31 | 27.9 | 1.48E-08 |
|  | BCB42614.1 porin OmpA | 94 | 32 | 34.0 | 3.55E-08 |
|  | BCB41870.1 peptidoglycan-associated lipoprotein | 88 | 26 | 29.5 | 5.03E-07 |
|  | BCB42216.1 outer membrane protein | 102 | 22 | 21.6 | 1.53E-04 |
|  | BCB42219.1 outer membrane protein | 91 | 22 | 24.2 | 0.01 |
|  | BCB44860.1 flagellar motor protein | 75 | 22 | 29.3 | 0.036 |
|  | BCB44907.1 outer membrane protein | 54 | 15 | 27.8 | 0.088 |
|  | BCB41514.1 flagellar motor protein PomB | 90 | 27 | 30.0 | 0.24 |
|  | BCB45303.1 chemotaxis protein LafU | 73 | 20 | 27.4 | 0.36 |
|  | BCB44186.1 ethanolamine ammonia-lyase light chain | 30 | 15 | 50.0 | 1.4 |
|  | BCB44897.1 molecular chaperone | 214 | 49 | 22.9 | 1.9 |
|  | BCB41138.1 30S ribosomal protein S11 | 94 | 27 | 28.7 | 2.1 |
|  | BCB43375.1 sigma-E factor regulatory protein RseB | 21 | 10 | 47.6 | 2.3 |
|  | BCB45461.1 mechanosensitive ion channel protein MscS | 82 | 22 | 26.8 | 2.5 |
|  | BCB43736.1 DNA-directed RNA polymerase subunit beta' | 96 | 22 | 22.9 | 3.2 |
|  | BCB42511.1 diguanylate phosphodiesterase | 38 | 14 | 36.8 | 7.1 |
|  | BCB41690.1 nucleoside permease | 24 | 10 | 41.7 | 9 |
| Mtg | BCB44153.1 murein transglycosylase | 248 | 248 | 100.0 | 0 |
|  | BCB42992.1 lytic transglycosylase | 140 | 60 | 42.9 | 1.89E-29 |
|  | BCB43424.1 membrane-bound lytic murein transglycosylase C | 159 | 42 | 26.4 | 2.85E-08 |
|  | BCB41381.1 peptidoglycan lytic exotransglycosylase | 105 | 32 | 30.5 | 3.37E-08 |
|  | BCB41376.1 murein transglycosylase | 134 | 41 | 30.6 | 6.93E-07 |
|  | BCB44811.1 nitrite reductase small subunit | 65 | 16 | 24.6 | 0.7 |
|  | BCB42221.1 calcium-binding protein | 38 | 14 | 36.8 | 7.4 |
|  | BCB44207.1 LysR family transcriptional regulator | 24 | 9 | 37.5 | 8.5 |
| FlgN2 | BCB44154.1 protein FlgN | 145 | 145 | 100.0 | 8.53E-107 |
|  | BCB42223.1 methyl-accepting chemotaxis protein | 79 | 22 | 27.8 | 0.64 |
|  | BCB42720.1 membrane protein | 114 | 25 | 21.9 | 0.98 |
|  | BCB43120.1 hypothetical protein Vag1382_22470 | 57 | 16 | 28.1 | 1.1 |
|  | BCB44406.1 transketolase 2 | 47 | 12 | 25.5 | 1.3 |
|  | BCB41203.1 phosphatase PAP2 family protein | 24 | 9 | 37.5 | 1.5 |
|  | BCB42208.1 N-acetyltransferase | 32 | 14 | 43.8 | 1.7 |
|  | BCB41780.1 methyl-accepting chemotaxis protein | 66 | 16 | 24.2 | 2.1 |
|  | BCB42043.1 hypothetical protein Vag1382_11690 | 32 | 11 | 34.4 | 2.3 |
|  | BCB41804.1 UPF0304 protein | 62 | 18 | 29.0 | 2.5 |
|  | BCB41714.1 hemolysin | 52 | 10 | 19.2 | 3.7 |
|  | BCB43930.1 GTP cyclohydrolase-2 | 21 | 10 | 47.6 | 4.2 |
|  | BCB41588.1 molecular chaperone | 20 | 10 | 50.0 | 4.7 |
|  | BCB43078.1 RecBCD enzyme subunit RecC | 67 | 16 | 23.9 | 9.5 |
|  | BCB45424.1 lactoylglutathione lyase | 22 | 8 | 36.4 | 9.9 |
| FlgM2 | BCB44155.1 flagellar biosynthesis anti-sigma factor FlgM | 93 | 93 | 100.0 | 1.98E-65 |
|  | BCB42587.1 histidine ammonia-lyase | 42 | 14 | 33.3 | 0.11 |
|  | BCB45001.1 methyltransferase | 33 | 12 | 36.4 | 2.1 |
|  | BCB44547.1 hypothetical protein Vag1382_36740 | 44 | 13 | 29.5 | 6.7 |
|  | BCB44059.1 peptidase | 54 | 21 | 38.9 | 9.5 |
|  | BCB44539.1 NADP-specific glutamate dehydrogenase | 44 | 13 | 29.5 | 9.7 |
| FlgA2 | BCB44156.1 flagella basal body P-ring formation protein FlgA | 265 | 265 | 100.0 | 0 |
|  | BCB41590.1 flagella basal body P-ring formation protein FlgA | 217 | 59 | 27.2 | 3.41E-20 |
|  | BCB43119.1 Flp pilus assembly protein CpaB | 64 | 18 | 28.1 | 0.83 |
|  | BCB41031.1 lipid A biosynthesis lauroyltransferase | 71 | 20 | 28.2 | 1.2 |
|  | BCB42400.1 LysR family transcriptional regulator | 41 | 12 | 29.3 | 1.5 |
|  | BCB43004.1 UDP-3-O-acylglucosamine N-acyltransferase | 72 | 20 | 27.8 | 2.1 |
|  | BCB42141.1 6-phosphogluconate dehydrogenase decarboxylating | 66 | 21 | 31.8 | 2.2 |
|  | BCB45358.1 outer membrane protein | 37 | 9 | 24.3 | 6.2 |
|  | BCB43246.1 ribosomal-protein-alanine acetyltransferase | 43 | 15 | 34.9 | 6.8 |
|  | BCB43417.1 non-canonical purine NTP pyrophosphatase | 64 | 17 | 26.6 | 7 |
|  | BCB44297.1 beta-ketoacyl-ACP reductase | 32 | 15 | 46.9 | 7.4 |
|  | BCB41547.1 ribosomal silencing factor RsfS | 36 | 13 | 36.1 | 9.6 |
| FlgB2 | BCB44157.1 flagellar basal body rod protein FlgB | 120 | 120 | 100.0 | 5.03E-87 |
|  | BCB41593.1 flagellar basal body rod protein FlgB | 130 | 50 | 38.5 | 7.56E-24 |
|  | BCB42976.1 uracil permease | 65 | 23 | 35.4 | 0.12 |
|  | BCB43540.1 alanine--glyoxylate aminotransferase | 67 | 18 | 26.9 | 0.89 |
|  | BCB40953.1 ubiquinone/menaquinone biosynthesis C-methyltransferase UbiE | 49 | 14 | 28.6 | 1.6 |
|  | BCB41851.1 gonadoliberin III | 22 | 10 | 45.5 | 3.4 |
|  | BCB42207.1 propionyl-CoA synthetase | 19 | 9 | 47.4 | 3.7 |
|  | BCB45316.1 DNA repair ATPase | 90 | 20 | 22.2 | 4.2 |
|  | BCB44771.1 aromatic amino acid aminotransferase | 30 | 11 | 36.7 | 6.4 |
|  | BCB41643.1 N-acetylglucosamine-6-phosphate deacetylase | 38 | 10 | 26.3 | 8.1 |
|  | BCB43856.1 SpoOM-like protein | 55 | 15 | 27.3 | 8.7 |
|  | BCB41199.1 3-isopropylmalate dehydratase large subunit | 35 | 11 | 31.4 | 9.5 |
|  | BCB41594.1 flagellar basal-body rod protein FlgC | 36 | 13 | 36.1 | 9.8 |
| FlgC2 | BCB44158.1 flagellar basal-body rod protein FlgC | 144 | 144 | 100.0 | 3.77E-105 |
|  | BCB41594.1 flagellar basal-body rod protein FlgC | 142 | 59 | 41.5 | 1.93E-35 |
|  | BCB41596.1 flagellar hook protein FlgE | 44 | 14 | 31.8 | 0.005 |
|  | BCB41596.1 flagellar hook protein FlgE | 81 | 22 | 27.2 | 0.82 |
|  | BCB45030.1 MFS transporter | 79 | 22 | 27.8 | 0.1 |
|  | BCB41843.1 two-component system response regulator TorR | 37 | 13 | 35.1 | 0.25 |
|  | BCB43143.1 hypothetical protein Vag1382_22700 | 76 | 19 | 25.0 | 0.82 |
|  | BCB44160.1 flagellar hook protein FlgE | 51 | 15 | 29.4 | 1.3 |
|  | BCB41598.1 flagellar basal-body rod protein FlgG | 45 | 13 | 28.9 | 1.6 |
|  | BCB45284.1 flagellar motor switch protein FliM | 39 | 14 | 35.9 | 1.9 |
|  | BCB43103.1 beta-galactosidase | 26 | 10 | 38.5 | 3 |
|  | BCB43206.1 hypothetical protein Vag1382_23330 | 74 | 18 | 24.3 | 4.1 |
|  | BCB42238.1 ribosomal RNA small subunit methyltransferase F | 85 | 26 | 30.6 | 4.5 |
|  | BCB40932.1 hypothetical protein Vag1382_00580 | 69 | 16 | 23.2 | 4.7 |
|  | BCB42424.1 L-ectoine synthase | 30 | 13 | 43.3 | 5.4 |
|  | BCB43942.1 putative response regulatory protein | 23 | 8 | 34.8 | 6.7 |
|  | BCB41960.1 long-chain-fatty-acid--CoA ligase | 49 | 15 | 30.6 | 8.6 |
| FlgD2 | BCB44159.1 basal-body rod modification protein FlgD | 227 | 227 | 100.0 | 5.59E-167 |
|  | BCB41595.1 basal-body rod modification protein FlgD | 147 | 40 | 27.2 | 8.08E-08 |
|  | BCB41457.1 sodium:alanine symporter | 20 | 9 | 45.0 | 4.1 |
|  | BCB43384.1 2-octaprenyl-6-methoxyphenol hydroxylase | 51 | 17 | 33.3 | 7.4 |
|  | BCB41973.1 ribosomal large subunit pseudouridine synthase B | 43 | 11 | 25.6 | 9.4 |
| FlgE2 | BCB44160.1 flagellar hook protein FlgE | 398 | 398 | 100.0 | 0 |
|  | BCB41596.1 flagellar hook protein FlgE | 440 | 151 | 34.3 | 1.68E-60 |
|  | BCB41598.1 flagellar basal-body rod protein FlgG | 111 | 43 | 38.7 | 2.12E-15 |
|  | BCB41598.1 flagellar basal-body rod protein FlgG | 132 | 44 | 33.3 | 1.18E-09 |
|  | BCB44162.1 flagellar basal-body rod protein FlgG | 85 | 33 | 38.8 | 1.43E-12 |
|  | BCB44162.1 flagellar basal-body rod protein FlgG | 131 | 37 | 28.2 | 1.81E-11 |
|  | BCB41597.1 flagellar basal body protein FlgF | 118 | 35 | 29.7 | 4.21E-06 |
|  | BCB41597.1 flagellar basal body protein FlgF | 43 | 16 | 37.2 | 5.03E-04 |
|  | BCB44161.1 flagellar basal body protein FlgF | 97 | 35 | 36.1 | 2.39E-05 |
|  | BCB44161.1 flagellar basal body protein FlgF | 43 | 16 | 37.2 | 8.74E-05 |
|  | BCB44166.1 flagellar hook-associated protein 1 FlgK | 64 | 25 | 39.1 | 4.13E-04 |
|  | BCB43575.1 glucose-6-phosphate isomerase | 55 | 16 | 29.1 | 2.7 |
|  | BCB44158.1 flagellar basal-body rod protein FlgC | 51 | 15 | 29.4 | 3.7 |
|  | BCB43484.1 UDP-N-acetylglucosamine 1-carboxyvinyltransferase | 106 | 31 | 29.2 | 4.9 |
|  | BCB41594.1 flagellar basal-body rod protein FlgC | 25 | 12 | 48.0 | 5.5 |
|  | BCB41594.1 flagellar basal-body rod protein FlgC | 37 | 10 | 27.0 | 6.4 |
|  | BCB44850.1 type VI secretion system protein ImpG | 28 | 13 | 46.4 | 5.8 |
|  | BCB42092.1 phospholipase D family protein | 58 | 16 | 27.6 | 8 |
|  | BCB43828.1 aminopeptidase | 32 | 13 | 40.6 | 8.1 |
| FlgF2 | BCB44161.1 flagellar basal body protein FlgF | 243 | 243 | 100.0 | 0 |
|  | BCB41597.1 flagellar basal body protein FlgF | 246 | 103 | 41.9 | 1.39E-55 |
|  | BCB41598.1 flagellar basal-body rod protein FlgG | 265 | 69 | 26.0 | 2.66E-11 |
|  | BCB44162.1 flagellar basal-body rod protein FlgG | 270 | 64 | 23.7 | 2.78E-10 |
|  | BCB44160.1 flagellar hook protein FlgE | 97 | 35 | 36.1 | 1.47E-05 |
|  | BCB44160.1 flagellar hook protein FlgE | 43 | 16 | 37.2 | 5.36E-05 |
|  | BCB41596.1 flagellar hook protein FlgE | 115 | 34 | 29.6 | 0.001 |
|  | BCB41596.1 flagellar hook protein FlgE | 37 | 12 | 32.4 | 0.44 |
|  | BCB41475.1 NAD kinase | 96 | 30 | 31.3 | 0.42 |
|  | BCB44113.1 DNA-binding transcriptional regulator | 22 | 11 | 50.0 | 0.81 |
|  | BCB44410.1 GGDEF-domain containing protein | 46 | 17 | 37.0 | 3.2 |
|  | BCB41545.1 penicillin-binding protein 2 | 28 | 11 | 39.3 | 6.1 |
|  | BCB41679.1 hypothetical protein Vag1382_08050 | 54 | 14 | 25.9 | 8.3 |
| FlgG2 | BCB44162.1 flagellar basal-body rod protein FlgG | 261 | 261 | 100.0 | 0 |
|  | BCB41598.1 flagellar basal-body rod protein FlgG | 262 | 143 | 54.6 | 1.05E-96 |
|  | BCB41597.1 flagellar basal body protein FlgF | 261 | 73 | 28.0 | 1.05E-21 |
|  | BCB44160.1 flagellar hook protein FlgE | 86 | 34 | 39.5 | 1.08E-12 |
|  | BCB44160.1 flagellar hook protein FlgE | 132 | 39 | 29.5 | 1.03E-11 |
|  | BCB41596.1 flagellar hook protein FlgE | 134 | 46 | 34.3 | 1.39E-12 |
|  | BCB41596.1 flagellar hook protein FlgE | 81 | 28 | 34.6 | 6.01E-08 |
|  | BCB44161.1 flagellar basal body protein FlgF | 267 | 64 | 24.0 | 4.03E-11 |
|  | BCB41594.1 flagellar basal-body rod protein FlgC | 40 | 16 | 40.0 | 3.43E-04 |
|  | BCB44166.1 flagellar hook-associated protein 1 FlgK | 110 | 29 | 26.4 | 0.047 |
|  | BCB41318.1 bifunctional aspartate kinase/homoserine dehydrogenase I | 15 | 10 | 66.7 | 1.1 |
|  | BCB41464.1 sodium:proton antiporter | 101 | 28 | 27.7 | 3.1 |
|  | BCB41602.1 flagellar hook protein FlgK | 38 | 13 | 34.2 | 3.9 |
|  | BCB41926.1 ATP phosphoribosyltransferase | 49 | 14 | 28.6 | 6.6 |
|  | BCB42782.1 iron-sulfur cluster carrier protein | 89 | 21 | 23.6 | 6.7 |
|  | BCB42218.1 hypothetical protein Vag1382_13440 | 15 | 8 | 53.3 | 7.1 |
|  | BCB44888.1 peptidase S8 | 56 | 15 | 26.8 | 7.4 |
|  | BCB44038.1 TldD protein | 57 | 19 | 33.3 | 8.4 |
| FlgH2 | BCB44163.1 flagellar L-ring protein 2 FlgH | 223 | 223 | 100.0 | 2.29E-168 |
|  | BCB41599.1 flagellar L-ring protein 1 FlgH | 198 | 73 | 36.9 | 1.79E-39 |
|  | BCB43177.1 hypothetical protein Vag1382_23040 | 31 | 11 | 35.5 | 0.55 |
|  | BCB42398.1 SM-20 | 20 | 10 | 50.0 | 4.9 |
|  | BCB45369.1 putative ribosome biogenesis GTPase RsgA 2 | 35 | 11 | 31.4 | 6.9 |
|  | BCB45350.1 phosphotyrosine protein phosphatase | 32 | 13 | 40.6 | 7.9 |
| FlgI2 | BCB44164.1 flagellar P-ring protein 2 FlgI | 373 | 373 | 100.0 | 0 |
|  | BCB41600.1 flagellar P-ring protein 1 FlgI | 362 | 178 | 49.2 | 6.85E-120 |
|  | BCB43102.1 transcriptional regulator EbgR | 58 | 18 | 31.0 | 2.7 |
|  | BCB44792.1 two-component system sensor histidine kinase UhpB | 74 | 17 | 23.0 | 4.1 |
|  | BCB42019.1 BCCT family transporter | 56 | 17 | 30.4 | 4.7 |
|  | BCB42351.1 sugar ABC transporter substrate-binding protein | 26 | 12 | 46.2 | 5.4 |
|  | BCB44766.1 molybdate ABC transporter substrate-binding protein | 59 | 15 | 25.4 | 6.4 |
|  | BCB43278.1 iron-sulfur cluster insertion protein ErpA | 30 | 10 | 33.3 | 6.6 |
|  | BCB42902.1 peptidase M16 | 37 | 14 | 37.8 | 7.8 |
|  | BCB43694.1 superoxide dismutase | 30 | 12 | 40.0 | 8.9 |
| FlgJ2 | BCB44165.1 flagellar protein FlgJ | 182 | 182 | 100.0 | 1.35E-132 |
|  | BCB41601.1 peptidoglycan hydrolase FlgJ | 95 | 33 | 34.7 | 4.96E-10 |
|  | BCB41677.1 ATP-dependent helicase | 45 | 13 | 28.9 | 4 |
|  | BCB42223.1 methyl-accepting chemotaxis protein | 65 | 24 | 36.9 | 4.6 |
|  | BCB43606.1 phosphoenolpyruvate carboxylase | 58 | 17 | 29.3 | 7.5 |
|  | BCB44629.1 catalase-peroxidase 2 | 59 | 15 | 25.4 | 9 |
|  | BCB40967.1 coproporphyrinogen III oxidase | 30 | 11 | 36.7 | 9.9 |
| FlgK2 | BCB44166.1 flagellar hook-associated protein 1 FlgK | 457 | 457 | 100.0 | 0 |
|  | BCB41602.1 flagellar hook protein FlgK | 325 | 78 | 24.0 | 2.96E-20 |
|  | BCB41602.1 flagellar hook protein FlgK | 40 | 20 | 50.0 | 1.40E-05 |
|  | BCB44160.1 flagellar hook protein FlgE | 64 | 25 | 39.1 | 4.74E-04 |
|  | BCB41596.1 flagellar hook protein FlgE | 38 | 16 | 42.1 | 0.008 |
|  | BCB41473.1 DNA repair protein RecN | 166 | 34 | 20.5 | 0.017 |
|  | BCB44162.1 flagellar basal-body rod protein FlgG | 110 | 29 | 26.4 | 0.093 |
|  | BCB43146.1 hypothetical protein Vag1382_22730 | 61 | 18 | 29.5 | 0.89 |
|  | BCB41597.1 flagellar basal body protein FlgF | 28 | 13 | 46.4 | 1.7 |
|  | BCB42777.1 sodium-independent anion transporter | 41 | 15 | 36.6 | 2.5 |
|  | BCB42904.1 fatty acid oxidation complex subunit alpha | 52 | 17 | 32.7 | 2.8 |
|  | BCB41594.1 flagellar basal-body rod protein FlgC | 37 | 12 | 32.4 | 3.6 |
|  | BCB41598.1 flagellar basal-body rod protein FlgG | 35 | 11 | 31.4 | 5.7 |
|  | BCB41296.1 carbamoyl-phosphate synthase large chain | 43 | 17 | 39.5 | 6.2 |
|  | BCB43501.1 magnesium transporter MgtE | 25 | 14 | 56.0 | 7.4 |
|  | BCB43734.1 NADH pyrophosphatase | 52 | 16 | 30.8 | 9.8 |
| FlgL2 | BCB44167.1 flagellar hook-associated protein 3 FlgL | 299 | 299 | 100.0 | 0 |
|  | BCB41603.1 flagellar hook-associated protein FlgL | 213 | 53 | 24.9 | 1.73E-13 |
|  | BCB41603.1 flagellar hook-associated protein FlgL | 69 | 23 | 33.3 | 3.94E-05 |
|  | BCB42955.1 polar flagellin F | 134 | 31 | 23.1 | 0.018 |
|  | BCB42953.1 polar flagellin A | 179 | 49 | 27.4 | 0.61 |
|  | BCB45292.1 lateral flagellin LafA | 113 | 27 | 23.9 | 0.68 |
|  | BCB41606.1 flagellin D | 53 | 16 | 30.2 | 0.69 |
|  | BCB42954.1 flagellin B | 53 | 16 | 30.2 | 0.71 |
|  | BCB43631.1 ABC transporter ATP-binding protein | 32 | 9 | 28.1 | 1.7 |
|  | BCB44072.1 biosynthetic arginine decarboxylase | 46 | 14 | 30.4 | 1.7 |
|  | BCB45379.1 glycosidase | 61 | 18 | 29.5 | 2.5 |
|  | BCB41666.1 zinc ABC transporter substrate-binding protein | 45 | 18 | 40.0 | 3.1 |
|  | BCB40968.1 coproporphyrinogen-III oxidase | 75 | 20 | 26.7 | 3.8 |
|  | BCB43257.1 polyribonucleotide nucleotidyltransferase | 36 | 13 | 36.1 | 4.4 |
|  | BCB41607.1 polar flagellin E | 94 | 23 | 24.5 | 4.7 |
|  | BCB43667.1 2 3-bisphosphoglycerate-independent phosphoglycerate mutase | 47 | 12 | 25.5 | 5.1 |
|  | BCB42223.1 methyl-accepting chemotaxis protein | 110 | 26 | 23.6 | 5.8 |
|  | BCB44842.1 nuclease SbcCD subunit C | 51 | 14 | 27.5 | 9.9 |
| Putative_fla | BCB44168.1 flagellin | 346 | 346 | 100.0 | 0 |
|  | BCB44952.1 glycine/betaine ABC transporter substrate-binding protein | 33 | 14 | 42.4 | 1.9 |
|  | BCB43633.1 hydrolase | 41 | 15 | 36.6 | 2.1 |
|  | BCB41069.1 hypothetical protein Vag1382_01950 | 41 | 13 | 31.7 | 7 |
|  | BCB43280.1 AI-2E family transporter | 38 | 14 | 36.8 | 8.6 |
|  | BCB43511.1 DUF3971 domain-containing protein | 20 | 9 | 45.0 | 9.4 |
| FliJ2 | BCB45276.1 flagellar export protein FliJ | 146 | 146 | 100.0 | 1.43E-105 |
|  | BCB41501.1 gamma-glutamyl phosphate reductase | 139 | 31 | 22.3 | 0.27 |
|  | BCB44700.1 transcriptional regulator | 95 | 20 | 21.1 | 0.83 |
|  | BCB44667.1 chitinase | 22 | 11 | 50.0 | 3.7 |
|  | BCB43803.1 heme biosynthesis protein HemY | 41 | 12 | 29.3 | 8.4 |
| FliI2 | BCB45277.1 flagellum-specific ATPase FliI | 448 | 448 | 100.0 | 0 |
|  | BCB42941.1 flagellum-specific ATPase FliI | 437 | 234 | 53.5 | 6.85E-166 |
|  | BCB42181.1 EscN/YscN/HrcN family type III secretion system ATPase | 415 | 175 | 42.2 | 1.47E-115 |
|  | BCB43876.1 ATP synthase subunit beta | 327 | 99 | 30.3 | 2.80E-38 |
|  | BCB43878.1 ATP synthase subunit alpha | 458 | 112 | 24.5 | 2.89E-35 |
|  | BCB43808.1 transcription termination factor Rho | 228 | 62 | 27.2 | 6.56E-11 |
|  | BCB42803.1 oligopeptide ABC transporter ATP-binding protein OppD | 97 | 28 | 28.9 | 0.041 |
|  | BCB42663.1 ABC transporter ATP-binding protein | 57 | 20 | 35.1 | 0.055 |
|  | BCB42716.1 macrolide export ATP-binding/permease protein MacB | 34 | 15 | 44.1 | 0.12 |
|  | BCB45281.1 flagellar hook-basal body complex protein FliE | 75 | 21 | 28.0 | 0.26 |
|  | BCB43719.1 ABC transporter ATP-binding protein | 36 | 12 | 33.3 | 0.33 |
|  | BCB41174.1 thiamine import ATP-binding protein ThiQ | 33 | 13 | 39.4 | 0.33 |
|  | BCB44308.1 D-alanyl-D-alanine carboxypeptidase | 86 | 25 | 29.1 | 0.44 |
|  | BCB41026.1 peptide ABC transporter ATP-binding protein | 39 | 16 | 41.0 | 0.77 |
|  | BCB42323.1 ABC-F family ATPase | 31 | 11 | 35.5 | 1.5 |
|  | BCB44431.1 peptidase M23 | 54 | 18 | 33.3 | 1.9 |
|  | BCB42430.1 glycine betaine/L-proline ABC transporter ATP-binding protein | 77 | 23 | 29.9 | 2.4 |
|  | BCB44710.1 ABC transporter ATP-binding protein | 24 | 10 | 41.7 | 2.6 |
|  | BCB43927.1 dipeptide/oligopeptide/nickel ABC transporter ATP-binding protein | 76 | 21 | 27.6 | 2.7 |
|  | BCB45191.1 multidrug ABC transporter ATP-binding protein | 33 | 10 | 30.3 | 2.9 |
|  | BCB41375.1 energy-dependent translational throttle protein EttA | 33 | 10 | 30.3 | 3.7 |
|  | BCB43534.1 maturase | 44 | 15 | 34.1 | 4.3 |
|  | BCB42586.1 urocanate hydratase | 29 | 12 | 41.4 | 4.9 |
|  | BCB43631.1 ABC transporter ATP-binding protein | 22 | 10 | 45.5 | 5.5 |
|  | BCB43631.1 ABC transporter ATP-binding protein | 33 | 10 | 30.3 | 6 |
|  | BCB41530.1 methionine import ATP-binding protein MetN | 42 | 15 | 35.7 | 5.5 |
|  | BCB43928.1 peptide ABC transporter ATP-binding protein | 28 | 11 | 39.3 | 6.5 |
|  | BCB45439.1 ABC transporter ATP-binding protein | 28 | 10 | 35.7 | 6.7 |
|  | BCB44875.1 nitrate ABC transporter ATP-binding protein | 41 | 14 | 34.1 | 7.7 |
|  | BCB42689.1 ABC transporter ATP-binding protein | 43 | 15 | 34.9 | 8.8 |
|  | BCB41750.1 multidrug resistance protein | 86 | 25 | 29.1 | 8.9 |
|  | BCB45076.1 ABC transporter ATP-binding protein | 49 | 20 | 40.8 | 9 |
|  | BCB44966.1 ABC transporter | 24 | 10 | 41.7 | 9.4 |
|  | BCB43312.1 ABC transporter ATP-binding protein | 36 | 14 | 38.9 | 9.5 |
| FliH2 | BCB45278.1 flagellar assembly protein FliH | 251 | 251 | 100.0 | 0 |
|  | BCB42942.1 flagellar assembly protein FliH | 195 | 57 | 29.2 | 2.57E-18 |
|  | BCB42161.1 type III secretion system protein | 179 | 40 | 22.3 | 1.37E-04 |
|  | BCB41442.1 LuxR family transcriptional regulator | 40 | 11 | 27.5 | 0.15 |
|  | BCB43876.1 ATP synthase subunit beta | 28 | 11 | 39.3 | 0.92 |
|  | BCB40909.1 peptide ABC transporter permease | 79 | 23 | 29.1 | 6.2 |
|  | BCB43837.1 gamma carbonic anhydrase family protein | 33 | 10 | 30.3 | 7.3 |
|  | BCB43887.1 tRNA uridine 5-carboxymethylaminomethyl modification enzyme MnmG | 50 | 16 | 32.0 | 9.2 |
|  | BCB42220.1 agglutination protein | 108 | 27 | 25.0 | 9.2 |
| FliG2 | BCB45279.1 flagellar motor switch protein FliG | 337 | 337 | 100.0 | 0 |
|  | BCB42943.1 flagellar motor switch protein FliG | 321 | 98 | 30.5 | 2.34E-53 |
|  | BCB42318.1 (Fe-S)-binding protein | 60 | 13 | 21.7 | 1.7 |
|  | BCB45129.1 hemin import ATP-binding protein HmuV | 34 | 12 | 35.3 | 2.9 |
|  | BCB44714.1 allophanate hydrolase | 122 | 35 | 28.7 | 2.9 |
|  | BCB42333.1 DTW domain-containing protein | 62 | 21 | 33.9 | 4.9 |
|  | BCB42200.1 bordetella uptake gene family protein | 108 | 29 | 26.9 | 5.7 |
|  | BCB45112.1 hypothetical protein Vag1382_42390 | 89 | 28 | 31.5 | 7.2 |
| FliF2 | BCB45280.1 flagellar M-ring protein FliF | 569 | 569 | 100.0 | 0 |
|  | BCB42944.1 flagellar M-ring protein FliF | 556 | 151 | 27.2 | 1.98E-55 |
|  | BCB42159.1 EscJ/YscJ/HrcJ family type III secretion inner membrane ring protein | 167 | 42 | 25.2 | 0.041 |
|  | BCB41095.1 nucleoside-diphosphate sugar epimerase | 49 | 18 | 36.7 | 0.26 |
|  | BCB42647.1 cytochrome c | 77 | 26 | 33.8 | 1.9 |
|  | BCB45313.1 glycyl radical enzyme | 34 | 17 | 50.0 | 3.6 |
|  | BCB42583.1 histidine utilization repressor | 26 | 10 | 38.5 | 3.6 |
|  | BCB41553.1 hemolysin | 37 | 10 | 27.0 | 3.9 |
|  | BCB41309.1 glutamate synthase | 51 | 19 | 37.3 | 5 |
|  | BCB44255.1 putative phosphoenolpyruvate synthase regulatory protein | 37 | 15 | 40.5 | 5.3 |
|  | BCB43723.1 transcriptional regulator CadC | 43 | 14 | 32.6 | 6.3 |
|  | BCB41002.1 hypothetical protein Vag1382_01280 | 31 | 13 | 41.9 | 7.3 |
| FliE2 | BCB45281.1 flagellar hook-basal body complex protein FliE | 118 | 118 | 100.0 | 2.77E-82 |
|  | BCB42945.1 flagellar hook-basal body complex protein FliE | 71 | 25 | 35.2 | 6.74E-11 |
|  | BCB45277.1 flagellum-specific ATPase FliI | 75 | 21 | 28.0 | 0.069 |
|  | BCB43601.1 bifunctional protein ArgH | 33 | 12 | 36.4 | 0.23 |
|  | BCB44437.1 sigma-54-dependent Fis family transcriptional regulator | 48 | 11 | 22.9 | 3.1 |
|  | BCB41939.1 1-aminocyclopropane-1-carboxylate deaminase | 31 | 9 | 29.0 | 4.5 |
|  | BCB45161.1 glycosyl transferase | 49 | 13 | 26.5 | 6.2 |
|  | BCB42941.1 flagellum-specific ATPase FliI | 80 | 24 | 30.0 | 7.8 |
|  | BCB45057.1 methyl-accepting chemotaxis protein | 56 | 17 | 30.4 | 9.4 |
| LafK | BCB45282.1 sigma-54-dependent Fis family transcriptional regulator LafK | 443 | 443 | 100.0 | 0 |
|  | BCB42946.1 sigma-54-dependent Fis family transcriptional regulator FlaM | 467 | 199 | 42.6 | 3.44E-117 |
|  | BCB42814.1 regulatory protein LuxO | 450 | 168 | 37.3 | 2.42E-86 |
|  | BCB43172.1 acetoacetate metabolism regulatory protein AtoC | 452 | 166 | 36.7 | 2.78E-78 |
|  | BCB42325.1 sigma-54-dependent Fis family transcriptional regulator | 325 | 133 | 40.9 | 8.57E-78 |
|  | BCB42354.1 sigma-54-dependent Fis family transcriptional regulator | 384 | 146 | 38.0 | 4.63E-76 |
|  | BCB40971.1 nitrogen regulation protein NR(I) | 390 | 141 | 36.2 | 5.34E-76 |
|  | BCB42948.1 sigma-54-dependent Fis family transcriptional regulator FlaK | 284 | 122 | 43.0 | 1.18E-73 |
|  | BCB41724.1 sigma-54-dependent Fis family transcriptional regulator | 386 | 156 | 40.4 | 2.34E-73 |
|  | BCB44437.1 sigma-54-dependent Fis family transcriptional regulator | 285 | 120 | 42.1 | 6.41E-72 |
|  | BCB41925.1 sigma-54-dependent Fis family transcriptional regulator | 285 | 117 | 41.1 | 8.52E-70 |
|  | BCB42666.1 anaerobic nitric oxide reductase transcription regulator | 224 | 109 | 48.7 | 1.06E-66 |
|  | BCB42045.1 TyrR family transcriptional regulator | 313 | 121 | 38.7 | 1.18E-64 |
|  | BCB40938.1 sigma-54-dependent Fis family transcriptional regulator | 312 | 119 | 38.1 | 1.64E-63 |
|  | BCB42684.1 phage shock protein operon transcriptional activator | 337 | 121 | 35.9 | 3.03E-59 |
|  | BCB44084.1 two-component system response regulator | 368 | 127 | 34.5 | 6.29E-59 |
|  | BCB42144.1 sigma-54-dependent Fis family transcriptional regulator | 296 | 110 | 37.2 | 2.85E-51 |
|  | BCB41338.1 sigma-54-dependent Fis family transcriptional regulator | 304 | 103 | 33.9 | 4.57E-49 |
|  | BCB41221.1 sigma-54-dependent Fis family transcriptional regulator | 299 | 95 | 31.8 | 2.72E-38 |
|  | BCB44663.1 two-component system response regulator | 412 | 93 | 22.6 | 1.83E-19 |
|  | BCB45455.1 DNA-binding response regulator | 329 | 75 | 22.8 | 4.05E-10 |
|  | BCB44348.1 DNA-binding response regulator | 121 | 35 | 28.9 | 1.08E-04 |
|  | BCB42438.1 DNA-binding response regulator | 112 | 32 | 28.6 | 1.23E-04 |
|  | BCB41942.1 DNA-binding response regulator | 134 | 36 | 26.9 | 5.46E-04 |
|  | BCB44754.1 DNA-binding response regulator | 116 | 34 | 29.3 | 6.48E-04 |
|  | BCB42654.1 DNA-binding response regulator | 116 | 27 | 23.3 | 0.001 |
|  | BCB41843.1 two-component system response regulator TorR | 136 | 36 | 26.5 | 0.001 |
|  | BCB41392.1 DNA-binding response regulator | 119 | 32 | 26.9 | 0.035 |
|  | BCB44762.1 diguanylate cyclase response regulator | 143 | 33 | 23.1 | 0.05 |
|  | BCB45263.1 DNA-binding response regulator | 170 | 35 | 20.6 | 0.081 |
|  | BCB45204.1 ATPase AAA | 139 | 36 | 25.9 | 0.11 |
|  | BCB44604.1 hybrid sensor histidine kinase/response regulator | 114 | 28 | 24.6 | 0.13 |
|  | BCB41107.1 ATP-dependent protease ATPase subunit HslU | 58 | 18 | 31.0 | 0.13 |
|  | BCB44892.1 DNA-binding response regulator | 113 | 28 | 24.8 | 0.16 |
|  | BCB44049.1 DNA-binding response regulator | 117 | 29 | 24.8 | 0.19 |
|  | BCB45189.1 DNA-binding response regulator | 68 | 20 | 29.4 | 0.24 |
|  | BCB43692.1 DNA-binding response regulator | 106 | 24 | 22.6 | 0.41 |
|  | BCB41215.1 DNA-binding response regulator | 117 | 29 | 24.8 | 0.71 |
|  | BCB45246.1 ATP-dependent protease | 71 | 22 | 31.0 | 0.97 |
|  | BCB44834.1 ATPase AAA | 151 | 36 | 23.8 | 1.3 |
|  | BCB44524.1 NAD/NADP-dependent betaine aldehyde dehydrogenase | 69 | 25 | 36.2 | 2.3 |
|  | BCB44392.1 DNA-binding response regulator | 41 | 15 | 36.6 | 2.4 |
|  | BCB43113.1 Flp pilus assembly protein | 45 | 15 | 33.3 | 2.6 |
|  | BCB43942.1 putative response regulatory protein | 31 | 12 | 38.7 | 2.9 |
|  | BCB45318.1 N-acetyltransferase | 25 | 9 | 36.0 | 4 |
|  | BCB43609.1 bifunctional aspartate kinase/homoserine dehydrogenase II | 78 | 22 | 28.2 | 4.7 |
|  | BCB43678.1 succinate dehydrogenase iron-sulfur subunit | 61 | 20 | 32.8 | 6.4 |
|  | BCB42732.1 tetrathionate reductase subunit A | 81 | 23 | 28.4 | 7.8 |
|  | BCB42136.1 transcriptional regulatory protein | 59 | 15 | 25.4 | 8.3 |
|  | BCB43717.1 DNA-binding protein Fis | 42 | 13 | 31.0 | 8.5 |
| MotY2 | BCB45283.1 sodium-type flagellar protein MotY | 339 | 339 | 100.0 | 0 |
|  | BCB42825.1 sodium-type flagellar protein MotY | 247 | 63 | 25.5 | 9.36E-24 |
|  | BCB41583.1 membrane protein | 103 | 41 | 39.8 | 5.83E-14 |
|  | BCB45023.1 membrane protein | 107 | 34 | 31.8 | 8.35E-11 |
|  | BCB42593.1 lipoprotein | 74 | 29 | 39.2 | 1.57E-10 |
|  | BCB44402.1 membrane protein | 101 | 33 | 32.7 | 2.57E-10 |
|  | BCB42614.1 porin OmpA | 102 | 36 | 35.3 | 1.27E-09 |
|  | BCB41870.1 peptidoglycan-associated lipoprotein | 100 | 31 | 31.0 | 1.32E-08 |
|  | BCB44142.1 membrane protein | 70 | 23 | 32.9 | 4.35E-07 |
|  | BCB42350.1 membrane protein | 86 | 24 | 27.9 | 9.77E-06 |
|  | BCB44907.1 outer membrane protein | 74 | 28 | 37.8 | 4.57E-05 |
|  | BCB44860.1 flagellar motor protein | 84 | 27 | 32.1 | 0.005 |
|  | BCB41514.1 flagellar motor protein PomB | 78 | 24 | 30.8 | 0.015 |
|  | BCB42480.1 chemotaxis protein MotB | 90 | 25 | 27.8 | 0.046 |
|  | BCB43507.1 RNA polymerase-associated protein RapA | 66 | 21 | 31.8 | 0.061 |
|  | BCB44436.1 hypothetical protein Vag1382_35630 | 88 | 26 | 29.5 | 0.59 |
|  | BCB41706.1 acyl carrier protein | 23 | 13 | 56.5 | 7.2 |
|  | BCB43990.1 peptidase | 52 | 15 | 28.8 | 7.8 |
| FliM2 | BCB45284.1 flagellar motor switch protein FliM | 272 | 272 | 100.0 | 0 |
|  | BCB42937.1 flagellar motor switch protein FliM | 204 | 45 | 22.1 | 4.92E-04 |
|  | BCB43958.1 hypothetical protein Vag1382_30850 | 71 | 18 | 25.4 | 0.81 |
|  | BCB44438.1 ClpV1 family T6SS ATPase | 51 | 16 | 31.4 | 1.7 |
|  | BCB41165.1 DUF490 domain-containing protein | 33 | 13 | 39.4 | 2.7 |
|  | BCB44158.1 flagellar basal-body rod protein FlgC | 39 | 14 | 35.9 | 3.6 |
| FliN2 | BCB45285.1 flagellar motor switch protein FliN | 123 | 123 | 100.0 | 4.01E-87 |
|  | BCB42936.1 flagellar motor switch protein FliN | 75 | 38 | 50.7 | 8.44E-23 |
|  | BCB42178.1 type III secretion system protein | 68 | 20 | 29.4 | 1.75E-07 |
|  | BCB40987.1 type II secretion system protein GspE | 112 | 29 | 25.9 | 0.23 |
|  | BCB44975.1 pyrrolidone-carboxylate peptidase | 46 | 11 | 23.9 | 0.38 |
|  | BCB42246.1 aminopeptidase | 72 | 24 | 33.3 | 1.8 |
|  | BCB42876.1 nucleoid-associated protein | 56 | 13 | 23.2 | 2.9 |
|  | BCB41208.1 acetolactate synthase small subunit | 61 | 21 | 34.4 | 3 |
|  | BCB45311.1 NAD-dependent dehydratase | 33 | 13 | 39.4 | 3.8 |
|  | BCB45463.1 peptide ABC transporter substrate-binding protein | 96 | 25 | 26.0 | 4.3 |
|  | BCB44347.1 hypothetical protein Vag1382_34740 | 34 | 9 | 26.5 | 4.8 |
|  | BCB42459.1 hypothetical protein Vag1382_15850 | 58 | 17 | 29.3 | 4.9 |
|  | BCB45388.1 hypothetical protein Vag1382_45150 | 54 | 13 | 24.1 | 5 |
|  | BCB42617.1 outer membrane usher protein | 72 | 18 | 25.0 | 6 |
|  | BCB42937.1 flagellar motor switch protein FliM | 30 | 11 | 36.7 | 6.5 |
|  | BCB43692.1 DNA-binding response regulator | 27 | 12 | 44.4 | 7.1 |
|  | BCB43837.1 gamma carbonic anhydrase family protein | 68 | 20 | 29.4 | 8.3 |
|  | BCB42266.1 hypothetical protein Vag1382_13920 | 31 | 10 | 32.3 | 8.4 |
|  | BCB41217.1 glycerol dehydrogenase | 27 | 9 | 33.3 | 8.6 |
|  | BCB45068.1 transporter | 33 | 13 | 39.4 | 8.8 |
|  | BCB43609.1 bifunctional aspartate kinase/homoserine dehydrogenase II | 87 | 21 | 24.1 | 9.4 |
|  | BCB41165.1 DUF490 domain-containing protein | 49 | 15 | 30.6 | 9.9 |
| FliP2 | BCB45286.1 flagellar biosynthetic protein FliP | 252 | 252 | 100.0 | 0 |
|  | BCB42934.1 flagellar biosynthetic protein FliP | 239 | 136 | 56.9 | 4.77E-89 |
|  | BCB42177.1 EscR/YscR/HrcR family type III secretion system export apparatus protein | 217 | 84 | 38.7 | 4.34E-44 |
|  | BCB44088.1 C4-dicarboxylate ABC transporter permease | 36 | 20 | 55.6 | 0.28 |
|  | BCB41462.1 aspartate aminotransferase family protein | 24 | 10 | 41.7 | 1.5 |
|  | BCB43060.1 hypothetical protein Vag1382_21870 | 53 | 19 | 35.8 | 1.8 |
|  | BCB41977.1 DNA polymerase | 36 | 13 | 36.1 | 3.3 |
| FliQ2 | BCB45287.1 flagellar export apparatus protein FliQ | 89 | 89 | 100.0 | 2.91E-60 |
|  | BCB42933.1 flagellar export apparatus protein FliQ | 85 | 46 | 54.1 | 7.81E-31 |
|  | BCB42176.1 EscS/YscS/HrcS family type III secretion system export apparatus protein | 56 | 15 | 26.8 | 6.35E-04 |
|  | BCB41260.1 outer membrane-stress sensor serine endopeptidase DegS | 41 | 14 | 34.1 | 2.6 |
|  | BCB43011.1 ribosome-recycling factor | 38 | 11 | 28.9 | 7.7 |
|  | BCB41948.1 hypothetical protein Vag1382_10740 | 44 | 18 | 40.9 | 7.7 |
|  | BCB44366.1 autoinducer 2 sensor kinase/phosphatase LuxQ | 52 | 12 | 23.1 | 8.5 |
| FliR2 | BCB45288.1 flagellar biosynthetic protein FliR | 258 | 258 | 100.0 | 0 |
|  | BCB42932.1 flagellar biosynthetic protein FliR | 230 | 79 | 34.3 | 9.81E-44 |
|  | BCB42175.1 EscT/YscT/HrcT family type III secretion system export apparatus protein | 149 | 36 | 24.2 | 3.35E-04 |
|  | BCB42117.1 hypothetical protein Vag1382_12430 | 35 | 15 | 42.9 | 3.6 |
|  | BCB44947.1 GntR family transcriptional regulator | 44 | 14 | 31.8 | 4.3 |
|  | BCB41259.1 DNA topoisomerase 4 subunit A | 27 | 9 | 33.3 | 9.2 |
| FlhB2 | BCB45289.1 flagellar biosynthesis protein FlhB | 375 | 375 | 100.0 | 0 |
|  | BCB42931.1 flagellar biosynthesis protein FlhB | 368 | 143 | 38.9 | 5.20E-91 |
|  | BCB42174.1 EscU/YscU/HrcU family type III secretion system export apparatus switch protein | 352 | 103 | 29.3 | 9.03E-52 |
|  | BCB41500.1 glutamate 5-kinase | 60 | 18 | 30.0 | 0.076 |
|  | BCB44522.1 GGDEF domain-containing protein | 72 | 19 | 26.4 | 0.23 |
|  | BCB43591.1 fimbrial protein | 65 | 20 | 30.8 | 0.56 |
|  | BCB44919.1 glycine/betaine ABC transporter | 137 | 34 | 24.8 | 3 |
|  | BCB45230.1 GGDEF domain-containing protein | 34 | 10 | 29.4 | 3.2 |
|  | BCB43300.1 serine protease | 70 | 19 | 27.1 | 5 |
|  | BCB41544.1 rod shape-determining protein RodA | 39 | 13 | 33.3 | 5.3 |
| FlhA2 | BCB45290.1 flagellar biosynthesis protein FlhA | 696 | 696 | 100.0 | 0 |
|  | BCB42930.1 flagellar biosynthesis protein FlhA | 697 | 351 | 50.4 | 0 |
|  | BCB42187.1 EscV/YscV/HrcV family type III secretion system export apparatus protein | 730 | 226 | 31.0 | 1.49E-111 |
|  | BCB43107.1 chorismate-binding protein | 139 | 36 | 25.9 | 0.19 |
|  | BCB42709.1 histidine kinase | 65 | 18 | 27.7 | 1.3 |
|  | BCB44631.1 hypothetical protein Vag1382_37580 | 25 | 11 | 44.0 | 2.2 |
|  | BCB42622.1 transcriptional regulator | 110 | 33 | 30.0 | 2.8 |
|  | BCB44980.1 membrane protein | 43 | 17 | 39.5 | 4.9 |
|  | BCB41220.1 hypothetical protein Vag1382_03460 | 50 | 18 | 36.0 | 6.4 |
| Dgc | BCB45291.1 diguanylate phosphodiesterase | 265 | 265 | 100.0 | 0 |
|  | BCB44679.1 diguanylate phosphodiesterase | 77 | 22 | 28.6 | 0.013 |
|  | BCB44255.1 putative phosphoenolpyruvate synthase regulatory protein | 161 | 36 | 22.4 | 0.071 |
|  | BCB43987.1 N-hydroxyarylamine O-acetyltransferase | 72 | 23 | 31.9 | 1.1 |
|  | BCB42864.1 lactoylglutathione lyase | 31 | 16 | 51.6 | 1.7 |
|  | BCB44912.1 arginine ABC transporter substrate-binding protein | 65 | 19 | 29.2 | 2.3 |
|  | BCB41461.1 O-acetylhomoserine aminocarboxypropyltransferase | 56 | 15 | 26.8 | 3.7 |
|  | BCB43072.1 amino-acid acetyltransferase | 52 | 13 | 25.0 | 4.8 |
|  | BCB40891.1 quinone oxidoreductase | 51 | 15 | 29.4 | 7.1 |
|  | BCB42858.1 chemotaxis protein | 118 | 27 | 22.9 | 8.7 |
| LafA | BCB45292.1 lateral flagellin LafA | 281 | 281 | 100.0 | 0 |
|  | BCB42955.1 polar flagellin F | 378 | 138 | 36.5 | 2.68E-59 |
|  | BCB41605.1 polar flagellin C | 384 | 133 | 34.6 | 1.51E-56 |
|  | BCB41606.1 flagellin D | 171 | 90 | 52.6 | 9.57E-49 |
|  | BCB41606.1 flagellin D | 79 | 36 | 45.6 | 3.83E-17 |
|  | BCB42954.1 flagellin B | 171 | 90 | 52.6 | 1.36E-48 |
|  | BCB42954.1 flagellin B | 79 | 36 | 45.6 | 4.17E-17 |
|  | BCB42953.1 polar flagellin A | 159 | 84 | 52.8 | 1.97E-45 |
|  | BCB42953.1 polar flagellin A | 99 | 40 | 40.4 | 4.07E-17 |
|  | BCB41607.1 polar flagellin E | 153 | 63 | 41.2 | 2.05E-29 |
|  | BCB41607.1 polar flagellin E | 76 | 26 | 34.2 | 1.79E-09 |
|  | BCB41918.1 adenylosuccinate lyase | 41 | 15 | 36.6 | 0.52 |
|  | BCB41603.1 flagellar hook-associated protein FlgL | 143 | 35 | 24.5 | 0.52 |
|  | BCB44167.1 flagellar hook-associated protein 3 FlgL | 113 | 27 | 23.9 | 0.63 |
|  | BCB44678.1 DEAD/DEAH box helicase | 54 | 20 | 37.0 | 3.3 |
|  | BCB43148.1 conjugal transfer protein TraG | 87 | 26 | 29.9 | 3.9 |
|  | BCB41170.1 UDP-N-acetylmuramate--L-alanyl-gamma-D-glutamyl-meso-2 6-diaminoheptandioate ligase | 59 | 17 | 28.8 | 4 |
|  | BCB44940.1 acyl-CoA dehydrogenase | 47 | 13 | 27.7 | 4 |
|  | BCB42572.1 membrane protein | 35 | 14 | 40.0 | 4.2 |
|  | BCB44399.1 methyl-accepting chemotaxis protein | 49 | 17 | 34.7 | 6.2 |
|  | BCB42369.1 ATP-dependent RNA helicase HrpA | 30 | 12 | 40.0 | 7.7 |
|  | BCB41988.1 ribonucleotide-diphosphate reductase subunit beta | 24 | 11 | 45.8 | 8.7 |
| Maf | BCB45293.1 hypothetical protein Vag1382_44200 | 440 | 440 | 100.0 | 0 |
|  | BCB43509.1 TldD protein | 90 | 23 | 25.6 | 0.69 |
|  | BCB42624.1 hypothetical protein Vag1382_17500 | 47 | 17 | 36.2 | 1.6 |
|  | BCB44323.1 phosphoethanolamine transferase | 66 | 20 | 30.3 | 3.1 |
|  | BCB41093.1 acetyltransferase | 30 | 8 | 26.7 | 5.2 |
|  | BCB43124.1 AMP-dependent synthetase | 58 | 18 | 31.0 | 5.3 |
|  | BCB43057.1 MFS transporter | 32 | 11 | 34.4 | 5.7 |
|  | BCB41371.1 phospho-2-dehydro-3-deoxyheptonate aldolase | 28 | 12 | 42.9 | 6 |
|  | BCB43925.1 translation elongation factor | 23 | 10 | 43.5 | 6.7 |
|  | BCB42486.1 hypothetical protein Vag1382_16120 | 26 | 8 | 30.8 | 6.8 |
| FliD2 | BCB45296.1 lateral flagellar hook-associated protein 2 | 445 | 445 | 100.0 | 0 |
|  | BCB42951.1 polar flagellar hook-associated protein 2 | 256 | 63 | 24.6 | 3.63E-17 |
|  | BCB42951.1 polar flagellar hook-associated protein 2 | 204 | 51 | 25.0 | 3.75E-12 |
|  | BCB42574.1 transcriptional regulator | 48 | 16 | 33.3 | 0.88 |
|  | BCB43694.1 superoxide dismutase | 141 | 32 | 22.7 | 1.6 |
|  | BCB41606.1 flagellin D | 59 | 19 | 32.2 | 3.1 |
|  | BCB42954.1 flagellin B | 59 | 19 | 32.2 | 3.1 |
|  | BCB44188.1 2-nitropropane dioxygenase | 31 | 9 | 29.0 | 4 |
|  | BCB45151.1 maltose ABC transporter substrate-binding protein MalE | 32 | 12 | 37.5 | 6.7 |
|  | BCB41296.1 carbamoyl-phosphate synthase large chain | 40 | 14 | 35.0 | 9.3 |
| FliS2 | BCB45297.1 flagellar protein FliS | 128 | 128 | 100.0 | 2.70E-92 |
|  | BCB42949.1 flagellar protein FliS | 119 | 36 | 30.3 | 3.66E-19 |
|  | BCB44152.1 DNA mismatch repair protein MutT | 52 | 14 | 26.9 | 1.4 |
|  | BCB45172.1 PTS fructose transporter subunit IIA | 38 | 9 | 23.7 | 1.8 |
|  | BCB42473.1 hypothetical protein Vag1382_15990 | 32 | 13 | 40.6 | 2.7 |
|  | BCB41827.1 ATP-dependent Clp protease ATP-binding subunit ClpA | 36 | 14 | 38.9 | 3.6 |
| FliT2 | BCB45298.1 hypothetical protein Vag1382_44250 | 106 | 106 | 100.0 | 1.90E-76 |
|  | BCB44289.1 transcription regulator | 41 | 15 | 36.6 | 0.1 |
|  | BCB42999.1 DNA-directed DNA polymerase | 41 | 14 | 34.1 | 0.61 |
|  | BCB42646.1 cytochrome c | 41 | 14 | 34.1 | 1.3 |
|  | BCB43382.1 hypothetical protein Vag1382_25090 | 80 | 23 | 28.8 | 1.7 |
|  | BCB44786.1 hypothetical protein Vag1382_39130 | 20 | 8 | 40.0 | 6.1 |
| FliK2 | BCB45299.1 flagellar hook-length control protein FliK | 357 | 357 | 100.0 | 0 |
|  | BCB42939.1 flagellar hook-length control protein FliK | 72 | 22 | 30.6 | 3.39E-06 |
|  | BCB41265.1 30S ribosomal protein S9 | 48 | 18 | 37.5 | 0.44 |
|  | BCB42740.1 orotidine 5'-phosphate decarboxylase | 50 | 14 | 28.0 | 6.6 |
|  | BCB45320.1 DNA-binding transcriptional regulator | 35 | 12 | 34.3 | 7.4 |
|  | BCB44534.1 sensor domain-containing phosphodiesterase | 89 | 25 | 28.1 | 7.5 |
| FliL2 | BCB45300.1 flagellar protein LafL | 166 | 166 | 100.0 | 6.69E-122 |
|  | BCB42938.1 flagellar basal body-associated protein FliL | 129 | 31 | 24.0 | 0.007 |
|  | BCB41444.1 plasmid replication protein | 47 | 18 | 38.3 | 0.18 |
|  | BCB43778.1 RNA polymerase subunit sigma | 67 | 19 | 28.4 | 1.4 |
|  | BCB42770.1 3-oxoacyl-[acyl-carrier-protein] synthase 3 protein 1 | 21 | 8 | 38.1 | 4 |
|  | BCB41373.1 peptidase M23 | 41 | 11 | 26.8 | 4.2 |
|  | BCB40890.1 LysR family transcriptional regulator | 58 | 17 | 29.3 | 4.7 |
|  | BCB44629.1 catalase-peroxidase 2 | 72 | 16 | 22.2 | 5.3 |
|  | BCB43640.1 acetylornithine aminotransferase | 86 | 21 | 24.4 | 5.3 |
|  | BCB42640.1 ABC transporter permease | 53 | 12 | 22.6 | 5.6 |
|  | BCB42390.1 Fe3+-hydroxamate ABC transporter permease FhuB | 29 | 12 | 41.4 | 7.1 |
|  | BCB44480.1 electron transfer flavoprotein subunit beta | 107 | 28 | 26.2 | 8.3 |
| FliA2 | BCB45301.1 RNA polymerase sigma factor for flagellar operon | 242 | 242 | 100.0 | 0 |
|  | BCB42927.1 RNA polymerase sigma factor FliA | 224 | 68 | 30.4 | 2.65E-35 |
|  | BCB41231.1 RNA polymerase sigma factor RpoD | 204 | 51 | 25.0 | 5.03E-05 |
|  | BCB42347.1 hypothetical protein Vag1382_14730 | 96 | 27 | 28.1 | 0.054 |
|  | BCB43298.1 UPF0231 protein | 34 | 11 | 32.4 | 3.1 |
|  | BCB41405.1 malate synthase | 36 | 14 | 38.9 | 9.5 |
| MotA2 | BCB45302.1 chemotaxis protein LafT | 285 | 285 | 100.0 | 0 |
|  | BCB41790.1 transcription-repair-coupling factor | 88 | 22 | 25.0 | 2.1 |
|  | BCB41019.1 biopolymer transporter ExbB | 77 | 24 | 31.2 | 2.8 |
|  | BCB43631.1 ABC transporter ATP-binding protein | 32 | 12 | 37.5 | 2.8 |
|  | BCB44053.1 flagellar motor protein MotA | 94 | 28 | 29.8 | 3.2 |
|  | BCB42960.1 hypothetical protein Vag1382_20870 | 17 | 9 | 52.9 | 4.3 |
|  | BCB42045.1 TyrR family transcriptional regulator | 107 | 28 | 26.2 | 7 |
| MotB2 | BCB45303.1 chemotaxis protein LafU | 330 | 330 | 100.0 | 0 |
|  | BCB41514.1 flagellar motor protein PomB | 152 | 45 | 29.6 | 1.08E-08 |
|  | BCB44907.1 outer membrane protein | 68 | 23 | 33.8 | 0.004 |
|  | BCB42480.1 chemotaxis protein MotB | 96 | 32 | 33.3 | 0.005 |
|  | BCB42825.1 sodium-type flagellar protein MotY | 73 | 20 | 27.4 | 0.4 |
|  | BCB42219.1 outer membrane protein | 110 | 29 | 26.4 | 0.93 |
|  | BCB41790.1 transcription-repair-coupling factor | 44 | 15 | 34.1 | 1.7 |
|  | BCB41583.1 membrane protein | 56 | 17 | 30.4 | 2.2 |
|  | BCB41111.1 DNA-binding transcriptional regulator CytR | 35 | 15 | 42.9 | 2.5 |
|  | BCB41022.1 microcin C ABC transporter permease YejB | 30 | 11 | 36.7 | 7.3 |
|  | BCB45451.1 AraC family transcriptional regulator | 45 | 11 | 24.4 | 8.3 |
