# Supplementary material for: Deciphering the genomes of motility-deficient mutants of Vibrio alginolyticus 138-2
Source: PeerJ. 2024 Mar 18;12:e17126. doi: 10.7717/peerj.17126 (PMC10956519; doi:10.7717/peerj.17126)
Supplement: Supplemental Information 8 — Six polar flagellin (flaA, flaB, mflaC, flaD, flaE, flaF) and one lateral flagellin (lafA) are listed and the degree of amino acid identities of the protein in each row to the protein in each column (the number in parentheses indicates the number of amino acids) is expressed as 100% of the number of amino acids in the column. [file peerj-12-17126-s008.docx]

|  | **flaA** | **flaB** | **flaC** | **flaD** | **flaE** | **flaF** | **lafA** |
| --- | --- | --- | --- | --- | --- | --- | --- |
| **flaA (376)** | **100** | **77.2** | **64.8** | **76.9** | **49.7** | **66.8** | **49.6** |
| **flaB (377)** | **77.2** | **100** | **66.6** | **99.7** | **53.3** | **69.8** | **50.4** |
| **flaC (384)** | **64.8** | **66.3** | **100** | **66.6** | **45.4** | **64.3** | **34.3** |
| **flaD (377)** | **76.9** | **99.7** | **66.8** | **100** | **53.3** | **69.8** | **50.4** |
| **flaE (374)** | **49.7** | **53.3** | **45.4** | **53.3** | **100** | **47.3** | **38.9** |
| **flaF (377)** | **66.8** | **69.8** | **64.3** | **69.8** | **47.3** | **100** | **52.3** |
| **lafA (281)** | **48.1** | **50.4** | **34.6** | **50.4** | **38.9** | **36.5** | **100** |

% identities
