# Supplementary figures and images for: Deciphering the genomes of motility-deficient mutants of Vibrio alginolyticus 138-2
Source: PeerJ. 2024 Mar 18;12:e17126. doi: 10.7717/peerj.17126 (PMC10956519; doi:10.7717/peerj.17126)

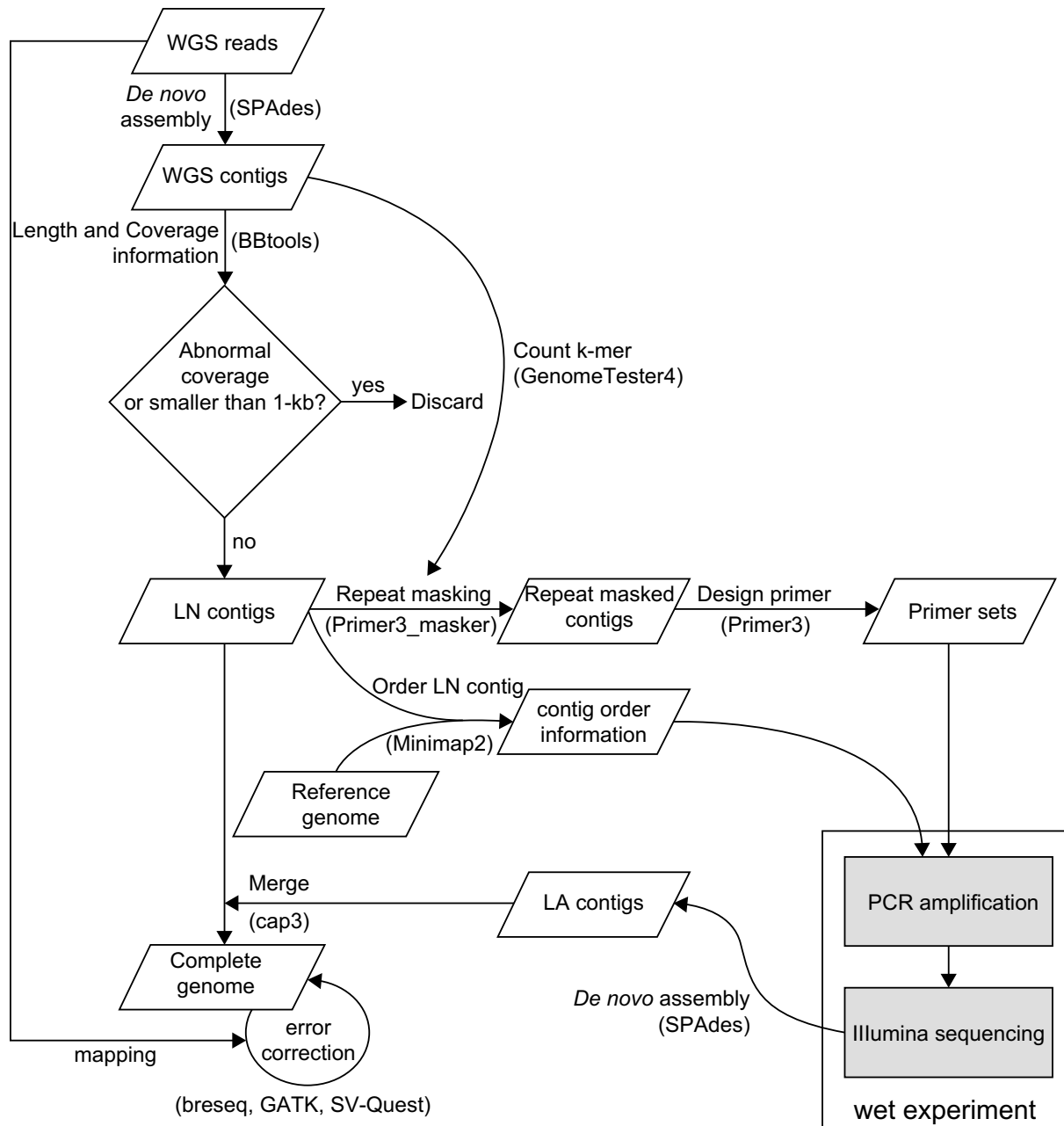

Supplement: Supplemental Information 9 — Quality-filtered whole genome shotgun sequencing reads (WGS Reads) are used as input data for this workflow. WGS reads are de novo assembled and filtered based on length and average coverage. The remaining long and non-repetitive contigs are called LN contigs (Long and Normal coverage contigs). The LN contigs are mapped to a very closely related genome to determine the order and orientation of each contig. Fragments between LN contigs are amplified by PCR using primers designed at both ends of the LN contigs. Amplified fragments (2kbp~10kbp) are multiplex-sequenced with MiSeq sequencer. Paired-end sequencing reads in each sample were locally assembled. These LA (Locally Assembled contig of PCR fragment) contigs were connected with LN contigs to produce two closed genome structures. White and grey boxes represent computational experiments and wet experiments, respectively. [file peerj-12-17126-s009.pdf]
